# Eligibility and subsequent burden of cardiovascular disease of four strategies for blood pressure-lowering treatment: a retrospective cohort study

**DOI:** 10.1016/S0140-6736(19)31359-5

**Published:** 2019-08-24

**Authors:** Emily Herrett, Sarah Gadd, Rod Jackson, Krishnan Bhaskaran, Elizabeth Williamson, Tjeerd van Staa, Reecha Sofat, Adam Timmis, Liam Smeeth

**Affiliations:** a Department of Non-Communicable Diseases Epidemiology, London School of Hygiene & Tropical Medicine, London, UK; b Department of Medical Statistics, London School of Hygiene & Tropical Medicine, London, UK; c Section of Epidemiology and Biostatistics, School of Population Health, University of Auckland, Auckland, New Zealand; d Health Data Research UK, London, UK; e Centre for Health Informatics, Division of Informatics, Imaging and Data Science, School of Health Sciences, Faculty of Biology, Medicine and Health, University of Manchester, Manchester Academic Health Science Centre, Manchester, UK; f Division of Pharmacoepidemiology and Clinical Pharmacology, Utrecht Institute of Pharmaceutical Sciences, Utrecht, Netherlands; g Institute of Health Informatics University College London, London, UK; h Barts Heart Centre, Queen Mary University London, London, UK

## Abstract

**Background:**

Worldwide treatment recommendations for lowering blood pressure continue to be guided predominantly by blood pressure thresholds, despite strong evidence that the benefits of blood pressure reduction are observed in patients across the blood pressure spectrum. In this study, we aimed to investigate the implications of alternative strategies for offering blood pressure treatment, using the UK as an illustrative example.

**Methods:**

We did a retrospective cohort study in primary care patients aged 30–79 years without cardiovascular disease, using data from the UK's Clinical Practice Research Datalink linked to Hospital Episode Statistics and Office for National Statistics mortality. We assessed and compared four different strategies to determine eligibility for treatment: using 2011 UK National Institute for Health and Care Excellence (NICE) guideline, or proposed 2019 NICE guideline, or blood pressure alone (threshold ≥140/90 mm Hg), or predicted 10-year cardiovascular risk alone (QRISK2 score ≥10%). Patients were followed up until the earliest occurrence of a cardiovascular disease diagnosis, death, or end of follow-up period (March 31, 2016). For each strategy, we estimated the proportion of patients eligible for treatment and number of cardiovascular events that could be prevented with treatment. We then estimated eligibility and number of events that would occur during 10 years in the UK general population.

**Findings:**

Between Jan 1, 2011, and March 31, 2016, 1 222 670 patients in the cohort were followed up for a median of 4·3 years (IQR 2·5–5·2). 271 963 (22·2%) patients were eligible for treatment under the 2011 NICE guideline, 327 429 (26·8%) under the proposed 2019 NICE guideline, 481 859 (39·4%) on the basis of a blood pressure threshold of 140/90 mm Hg or higher, and 357 840 (29·3%) on the basis of a QRISK2 threshold of 10% or higher. During follow-up, 32 183 patients were diagnosed with cardiovascular disease (overall rate 7·1 per 1000 person-years, 95% CI 7·0–7·2). Cardiovascular event rates in patients eligible for each strategy were 15·2 per 1000 person-years (95% CI 15·0–15·5) under the 2011 NICE guideline, 14·9 (14·7–15·1) under the proposed 2019 NICE guideline, 11·4 (11·3–11·6) with blood pressure threshold alone, and 16·9 (16·7–17·1) with QRISK2 threshold alone. Scaled to the UK population, we estimated that 233 152 events would be avoided under the 2011 NICE guideline (28 patients needed to treat for 10 years to avoid one event), 270 233 under the 2019 NICE guideline (29 patients), 301 523 using a blood pressure threshold (38 patients), and 322 921 using QRISK2 threshold (27 patients).

**Interpretation:**

A cardiovascular risk-based strategy (QRISK2 ≥10%) could prevent over a third more cardiovascular disease events than the 2011 NICE guideline and a fifth more than the 2019 NICE guideline, with similar efficiency regarding number treated per event avoided.

**Funding:**

National Institute for Health Research.

## Introduction

Randomised trials^1^, ^2^ have shown that blood pressure reductions, whether by diet, lifestyle, or drug therapy, can reduce the risk of cardiovascular disease. A reduction in systolic blood pressure of 10 mm Hg can reduce the incidence of stroke by roughly 40% and myocardial infarction by roughly 20%.^1^ Globally, most blood pressure treatment recommendations are based on blood pressure levels.^3^, ^4^, ^5^

In the UK, guidance^4^ from the National Institute for Health and Care Excellence (NICE) recommends that clinicians consider pharmacological intervention for primary prevention of cardiovascular disease when a patient's clinic blood pressure is between 140/90 and 159/99 mm Hg and they have one or more of the following: an absolute 10-year cardiovascular risk of 20% or higher (proposed 2019 NICE guideline^6^ lowers this to ≥10%), diabetes, kidney disease, or target organ damage. Blood pressure lowering with pharmacological intervention is not recommended for patients with blood pressure lower than 140/90 mm Hg, even if their cardiovascular risk is high, whereas patients with blood pressure of 160/100 mm Hg or higher are recommended for treatment even when cardiovascular disease risk is low. Similarly, the 2018 European guidelines^5^ suggest treatment when a patient has stage 1 hypertension (140–159/90–99 mm Hg) and has one of more of the following: high 10-year risk of cardiovascular disease mortality (on the basis of the Systematic Coronary Risk Evaluation system [SCORE]), renal disease, target organ damage, or blood pressure that is not controlled after 3–6 months of lifestyle intervention. The guidelines^3^ of the American College of Cardiology and American Heart Association (ACC/AHA) have a blood pressure threshold for treatment of 130–139/80–89 mm Hg if the estimated 10-year cardiovascular risk (based on the ACC/AHA pooled cohort equations) is 10% or higher. All patients with blood pressure 140–90 mm Hg or higher are eligible for blood pressure-lowering medication, with a target blood pressure of 130/80 mm Hg.

**Research in context**

**Evidence before this study**

Strong evidence has shown that the benefits of blood pressure reduction are observed in patients across the blood pressure spectrum and that the absolute benefits of treatment are proportional to a patient's pretreament absolute cardiovascular risk. However, blood pressure treatment guidelines worldwide continue to be guided predominantly by blood pressure thresholds and the concept of hypertension. We searched PubMed for studies published in English from Feb 11, 2009, to Feb 11, 2019, that compared a risk-based approach to blood pressure treatment with either a blood pressure-only approach or a combination approach (using both blood pressure and risk to determine treatment, as set out in US, UK, and European guidelines). For this search, we used the terms “blood pressure lowering OR blood pressure treatment OR antihypertensive*” AND “strateg* OR approach OR policy” AND “compare OR contrast” AND “cardiovascular risk OR cardiovascular disease risk OR absolute risk OR QRISK2”. The search generated 228 studies, of which five were relevant; three showed the superiority of a risk-based approach over an entirely blood pressure-based approach. Two studies from low-income and middle-income countries additionally investigated combination approaches: both showed that a risk-based approach was superior to both a blood pressure only and a combination approach. All studies used trial populations or simulated data to compare strategies. Therefore, the potential effect of using a risk-based approach to determine treatment eligibility and subsequent cardiovascular disease burden in a real-life population was unclear.

**Added value of this study**

Using the UK as an illustrative population, our study showed that determining eligibility for blood pressure treatment with an absolute cardiovascular risk cutoff of 10% or higher (based on QRISK2) would increase the proportion of people who were eligible for treatment by 32%, compared with that of the 2011 National Institute for Health and Care Excellence (NICE) UK guideline. This QRISK2-based strategy would prevent over a third more cardiovascular disease events than would the 2011 NICE guideline, with the same efficiency, and would also prevent a fifth more events than the proposed 2019 NICE guideline, with similar or better efficiency. Over 10 years, we estimated that an absolute risk-based guideline could prevent nearly 90 000 more cardiovascular events in the UK than the 2011 NICE guideline that is currently in use.

**Implications of all the available evidence**

Our analyses and the evidence to date illustrate that the potential benefits of an absolute risk-based approach to assess eligibility for blood pressure-lowering treatment surpass those of approaches recommended by international guidelines. These findings show the need to re-assess blood pressure treatment guidelines that are based on blood pressure cutoffs.

However, strong evidence suggests that using a blood pressure threshold to make treatment decisions might not be the optimal strategy. Meta-analyses of randomised and non-randomised studies^1^, ^7^ have shown that treating patients with blood pressures as low as 110/70 mm Hg (lower than the NICE and European Society of Cardiology guideline threshold of 140/90 mm Hg) can reduce cardiovascular risk. Additionally, the absolute treatment benefit of blood pressure-lowering drugs is determined by a patient's absolute cardiovascular risk, with more benefit achieved in patients with the highest risk, even when blood pressure is lower than treatment thresholds.^8^ Blood pressure is just one component of absolute cardiovascular risk, meaning that patients with blood pressure lower than guideline treatment thresholds could have very high risk, and patients with blood pressure higher than those thresholds could be at low risk.^9^ This suggests that many patients who might benefit from blood pressure-lowering treatment are missing out, whereas others might be overtreated.

Two key considerations in determining the optimal strategy for blood pressure-lowering treatment are the number of patients who would be eligible for treatment and the degree to which treatment is prioritised to those people most likely to have future cardiovascular disease. The 2011 NICE guidelines^4^ and their proposed 2019 update^6^ are positioned between two strategies: blood pressure-only treatment and risk-based treatment.

Before the 2011 NICE guidance, treatment recommendations were based solely on blood pressure thresholds, treating patients with hypertension. Some evidence exists that general practitioners continue to use a blood pressure threshold of 140/90 mm Hg to guide treatment decisions.^10^ However, the aim of lowering blood pressure is to reduce cardiovascular risk. A rational strategy to determine who to treat would be to target patients at high cardiovascular risk rather than only those with hypertension. Such a strategy would involve the use of a validated cardiovascular risk score (such as QRISK2,^11^ SCORE,^12^ or the ACC/AHA pooled equations,^13^ calculated on the basis of blood pressure and other cardiovascular risk factors) to determine treatment eligibility. This approach has been adopted for lipid lowering in the UK: patients whose absolute QRISK2 10-year cardiovascular risk is 10% or higher are recommended for treatment.^14^

In our study, we investigated how these alternative strategies would affect eligibility for blood pressure-lowering treatment in the UK. Our aim was to describe and compare eligibility for blood pressure-lowering treatment of four different strategies, relate this to subsequent cardiovascular disease burden, and estimate the efficiency of each strategy on the basis of the number treated over 10 years to avoid a cardiovascular disease diagnosis.

## Methods

### Study design and participants

We did a retrospective cohort study using data from the UK's Clinical Practice Research Datalink (CPRD) linked to Hospital Episode Statistics (HES) and mortality data from the Office for National Statistics (ONS). The CPRD is a primary care database containing anonymised data from approximately 6·9% of the UK population.^15^, ^16^ Data from CPRD include diagnoses, tests, clinical measurements, prescriptions, and specialist referrals. EH had full access to the CPRD database to create the study population. Approval was given from the CPRD's Independent Scientific Advisory Committee (17_146). The database is broadly representative of the UK population in terms of age and sex, with a slightly lower proportion of patients from younger age groups and from the north of England than that of the whole UK population.^15^ HES holds data on diagnoses made and procedures done in English hospitals, and the ONS mortality data hold the date and cause of deaths registered in England and Wales.

Participants were aged 30–79 years and registered with a participating CPRD practice (appendix p 11) on Jan 1, 2011, chosen as a start date to allow follow-up for up to 5 years. Few patients younger than 30 years would meet treatment eligibility criteria for any strategy, and those aged 80 years or older required careful consideration and are treated differently than younger patients in the 2011 guidance.^4^ Patients were excluded if, at enrolment in the cohort, they had less than 1 year of follow-up, were pregnant, had an existing diagnosis of cardiovascular disease (recorded in CPRD or HES; appendix p 19), or had a last blood pressure reading taken more than 5 years before enrolment. We did not exclude patients with contraindications to any specific blood pressure-lowering drug. Cohort size was determined by the number of patients in the CPRD who met the inclusion criteria. The study protocol was approved by the London School of Hygiene & Tropical Medicine (14334).

### Procedures

We investigated four strategies to define treatment (panel), with eligibility based on the following: first, blood pressure of 140/90 mm Hg or higher alone; second, 2011 NICE guidelines;^4^ third, proposed 2019 NICE guidelines;^6^ and fourth, absolute QRISK2 10-year cardiovascular risk score of 10% or higher alone. QRISK2 was calculated with the algorithms supplied online and Read codes from the Quality and Outcomes Framework.^19^ Mirroring the QRISK2 algorithm in clinical practice, our algorithm used a population-average imputation approach to account for missing data. Target organ damage was defined in the CPRD with Read codes recorded before the cohort start date.

Panel **Four strategies to assess eligibility for blood pressure-lowering treatment**

**Blood pressure threshold alone**

Patients were eligible if they either had a blood pressure of 140/90 mm Hg or higher in the last measurement before enrolment or were using blood pressure-lowering medication at enrolment and had one of the following: two previous blood pressure measures of 140/90 mm Hg or higher within a 3-month period,^17^, ^18^ a hypertension diagnosis, or a flag on the general practice hypertension register.

**2011 National Institute for Health and Care Excellence (NICE) guideline**^4^

Patients were eligible if they either met the criteria for the blood pressure strategy plus one or more of target organ damage,* QRISK2 score of 20% or higher, renal disease, or diabetes; were using blood pressure-lowering medication at enrolment and had two previous blood pressure measures of 160/100 mm Hg or higher within a 3-month period, a diagnostic code for stage 2 hypertension, or severe hypertension; or had blood pressure of 160/100 mm Hg or higher in the last measurement before enrolment.

**Proposed 2019 NICE guideline**

Patients were eligible if they either met the criteria for the blood pressure strategy plus one or more of target organ damage,* QRISK2 score of 10% or higher, renal disease, or diabetes; were using blood pressure-lowering medication at enrolment and had two previous blood pressure measures of 160/100 mm Hg or higher within a 3-month period, a diagnostic code for stage 2 hypertension, or severe hypertension; or had blood pressure of 160/100 mm Hg or higher in the last measurement before enrolment.

**QRISK2 threshold alone**

Patients were eligible if they had a QRISK2† score of 10% or higher at enrolment.

Patients were followed up from Jan 1, 2011, and were categorised as eligible or ineligible for blood pressure-lowering treatment under the four different strategies (panel). Patients were followed up until the earliest occurrence of a cardiovascular disease diagnosis (primary outcome), death, or end of CPRD follow-up or until March 31, 2016 (the last available date when linked data from HES were accessible).

A cardiovascular disease diagnosis was defined as the following: any record of coronary heart disease (myocardial infarction, angina, revascularisation procedures, or coronary heart disease not otherwise specified) or cerebrovascular disease (stroke [not including haemorrhagic stroke because this is not included in QRISK2 outcomes],^11^ transient ischaemic attack, or non-stroke cerebrovascular disease). These outcomes included any record from CPRD, HES, or ONS, and were defined according to Read codes and the codes of the International Classification of Diseases, tenth edition (appendix p 19). The primary analysis estimated the proportion of patients who would be eligible for treatment under each strategy, as well as the rate and proportion of cardiovascular disease events that occurred during follow-up, stratified by eligibility.

### Statistical analysis

For each strategy, we assessed eligibility for treatment at enrolment and compared the number, demographic characteristics, and risk factor profiles of eligible patients. These numbers were then used to estimate eligibility in the 2011 UK population (on the basis of the national census).^20^ Under each strategy, we calculated the total number of patients diagnosed with cardiovascular disease and the overall crude rate of cardiovascular disease among patients eligible and ineligible for treatment. This was extrapolated to calculate the number of events that would occur during 10 years of follow-up in the UK population.

We estimated the number of patients needed to treat (NNT) to avoid an event to compare the efficiency of each strategy. For each strategy, NNT was calculated as the number of patients eligible for treatment divided by the number of events avoided if all eligible patients were treated for 10 years, assuming a 20% reduction in risk in treated patients (appendix p 2).

We did secondary analysis to further investigate the predictive ability of blood pressure and QRISK2 for subsequent cardiovascular disease. Each category was split into the following standard groups: systolic blood pressure at enrolment (<110, 110–119, 120–139, 140–159, 160–179, or ≥180 mm Hg), diastolic blood pressure at enrolment (<70, 70–79, 80–89, 90–99, 100–109, or ≥110 mm Hg), and QRISK2 at enrolment (<10%, 10–19%, 20–29%, or ≥30%). Systolic blood pressure, diastolic blood pressure, and QRISK2 10-year risk score were also split into quintile groups. In each group, the rate and proportion of cardiovascular disease events were calculated and compared across treatment strategies.

To maximise comparability between strategies, we stratified rates of cardiovascular disease according to use of blood pressure-lowering treatment at enrolment (on the basis of an issued prescription at enrolment) and whether their blood pressures were higher or lower than 140/90 mm Hg. Treated systolic blood pressures at enrolment were also plotted.

We did three post-hoc sensitivity analyses. We calculated NNT for 5 years to prevent one cardiovascular event to assess whether extrapolation of our rates to 10 years was an acceptable approach. We assessed the primary outcome restricted to acute events (myocardial infarction and stroke). Lastly, we assessed the rate of haemorrhagic stroke by eligibility for each strategy. QRISK2 does not include haemorrhagic stroke, an important outcome when considering blood pressure treatment.

All analyses were done with Stata (version 14).

### Role of the funding source

The funders of the study had no role in study design, data collection, data analysis, data interpretation, or writing of the report. The corresponding author had full access to all the data in the study and had final responsibility for the decision to submit for publication.

## Results

Between Jan 1, 2011, and March 31, 2016, 1 222 670 patients in the cohort were followed up for a median of 4·3 years (IQR 2·5–5·2; total follow-up 4·5 million person-years). During follow-up, 32 183 patients were diagnosed with cardiovascular disease (overall rate 7·1 per 1000 person-years, 95% CI 7·0–7·2). Median age at entry was 51 years (IQR 41–62). Cohort demographic and risk factor characteristics are shown for the total cohort and by eligibility for each strategy in table 1 and the appendix (pp 4–5).

**Table 1:** Demographic and risk factor characteristics of patients at enrolment

		**Whole cohort**	**Blood pressure threshold**	**2011 NICE guideline**	**Proposed 2019 NICE guideline**	**QRISK2 10-year risk threshold**
Number of patients eligible		1 222 670 (100·0%)	481 859 (39·4%)	271 963 (22·2%)	327 429 (26·8%)	357 840 (29·3%)
Follow-up time, median (IQR; years)		4·3 (2·5–5·2)	4·3 (2·5–5·2)	4·2 (2·5–5·2)	4·2 (2·5–5·2)	3·9 (2·4–5·2)
Sex						
	Men	530 618 (43·4%)	242 423 (50·3%)	134 958 (49·6%)	168 143 (51·4%)	209 882 (58·7%)
	Women	692 052 (56·6%)	239 436 (49·7%)	137 005 (50·4%)	159 286 (48·6%)	147 958 (41·3%)
Age (years)*						
	30–39	251 294 (20·6%)	38 399 (8·0%)	9270 (3·4%)	9293 (2·8%)	709 (0·2%)
	40–49	317 280 (25·9%)	86 909 (18·0%)	31 623 (11·6%)	32 737 (10·0%)	9397 (2·6%)
	50–59	281 786 (23·0%)	121 185 (25·1%)	58 146 (21·4%)	69 414 (21·2%)	55 263 (15·4%)
	60–69	238 774 (19·5%)	139 937 (29·0%)	87 968 (32·3%)	120 577 (36·8%)	159 184 (44·5%)
	70–79	133 536 (10·9%)	95 429 (19·8%)	84 956 (31·2%)	95 408 (29·1%)	133 287 (37·2%)
QRISK2 risk score						
	<10%	864 830 (70·7%)	235 788 (48·9%)	81 358 (29·9%)	81 358 (24·8%)	0
	10–19%	216 349 (17·7%)	136 578 (28·3%)	81 112 (29·8%)	136 578 (41·7%)	216 349 (60·5%)
	20–29%	95 167 (7·8%)	71 019 (14·7%)	71 019 (26·1%)	71 019 (21·7%)	95 167 (26·6%)
	≥30%	46 324 (3·8%)	38 474 (8·0%)	38 474 (14·1%)	38 474 (11·8%)	46 324 (12·9%)
Systolic blood pressure (mm Hg)*						
	<110	100 345 (8·2%)	2876 (0·6%)	2348 (0·9%)	2489 (0·8%)	6660 (1·9%)
	110–119	187 402 (15·3%)	13 123 (2·7%)	9929 (3·7%)	10 610 (3·2%)	21 811 (6·1%)
	120–139	603 024 (49·3%)	133 961 (27·8%)	92 186 (33·9%)	100 308 (30·6%)	162 640 (45·5%)
	140–159	289 745 (23·7%)	289 745 (60·1%)	125 346 (46·1%)	171 868 (52·5%)	139 895 (39·1%)
	160–179	35 618 (2·9%)	35 618 (7·4%)	35 618 (13·1%)	35 618 (10·9%)	21 939 (6·1%)
	≥180	6536 (0·5%)	6536 (1·4%)	6536 (2·4%)	6536 (2·0%)	4895 (1·4%)
Systolic blood pressure (mm Hg)*		129·2 (15·6)	141·9 (12·9)	142·4 (15·6)	142·5 (14·6)	137·5 (14·8)
Diastolic blood pressure (mm Hg)*		78·2 (9·5)	83·4 (9·5)	82·5 (10·7)	82·5 (10·2)	79·3 (9·3)
Patients using blood pressure-lowering drugs		223 465 (18·3%)	213 046 (44·2%)	190 401 (70·0%)	200 214 (61·1%)	153 586 (42·9%)

Data are n (%) or mean (SD), unless otherwise specified. Characteristics of patients of the whole cohort and patients eligible for treatment under each strategy: blood pressure threshold of 140/90 mm Hg or higher, 2011 NICE guideline, proposed 2019 NICE guideline, and QRISK2 10-year risk of 10% or higher. NICE=National Institute for Health and Care Excellence.

* Closest measure before enrolment on Jan 1, 2011.

**Figure 1:**
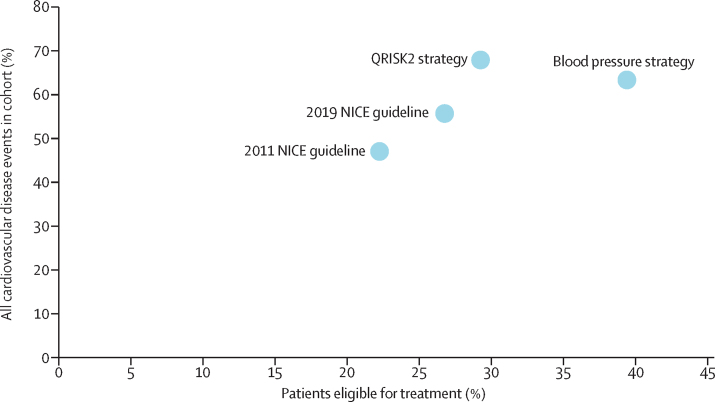
Proportion of patients who were eligible for blood pressure-lowering treatment under four strategies versus the proportion of all subsequent cardiovascular disease events that occurred among eligible patients in the cohort The four strategies are the following: blood pressure threshold of 140/90 mm Hg or higher, 2011 NICE guideline, proposed 2019 NICE guideline, and QRISK2 10-year risk of 10% or higher. NICE=National Institute for Health and Care Excellence.

**Figure 2:**
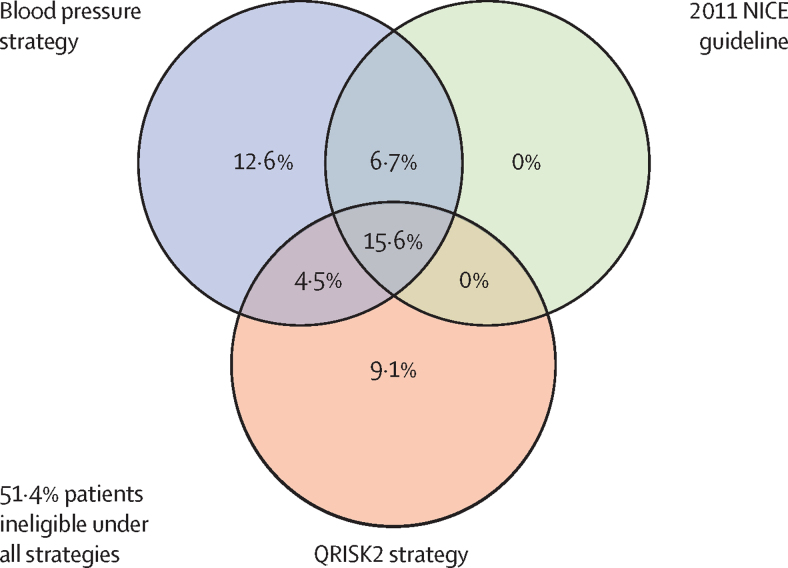
Eligibility of patients for blood pressure-lowering treatment based on three strategies The three strategies are the following: blood pressure threshold of 140/90 mm Hg or higher, 2011 NICE guideline, and QRISK2 10-year risk of 10% or higher. NICE=National Institute for Health and Care Excellence.

Of the 1 222 670 patients in the cohort, 271 963 (22·2%) were eligible for treatment at cohort entry under the 2011 NICE guideline, 327 429 (26·8%) were eligible under the proposed 2019 NICE guideline, 481 859 (39·4%) were eligible on the basis of blood pressure alone (treating those with hypertension), and 357 840 (29·3%) were eligible on the basis of a QRISK2 10-year risk of 10% or higher (figure 1, table 1). For the strategy based on blood pressure alone, we found a 17·2% increase in eligibility compared with that for the strategy based on the 2011 NICE guideline (appendix p 6); for the QRISK2 strategy, we found a 7·0% net increase in eligibility, with some patients gaining (13·7% of the cohort) and others losing eligibility (6·7%) compared with that of the 2011 NICE guideline. The proposed 2019 NICE guideline resulted in 4·5% additional eligible patients compared with the 2011 NICE guideline, with no patients losing eligibility under this strategy. The overlap in eligibility between the 2011 NICE guideline, blood pressure alone, and QRISK2 strategies are shown in figure 2. A comparison between blood pressure alone, QRISK2, and the 2019 NICE guideline and a breakdown of treatment according to eligibility are shown in the appendix (p 12).

We assessed how eligibility for treatment changed from the 2011 NICE guideline to the QRISK2 strategy: patients who lost eligibility with QRISK2 tended to be younger and had fewer cardiovascular risk factors (were more often non-smokers and had lower systolic blood pressures, and fewer patients had diabetes; appendix p 8). 61·4% of patients who lost eligibility were using blood pressure-lowering medication at enrolment.

Of the 32 183 cardiovascular events that occurred during follow-up, 15 136 (47·0%) were in patients eligible under the 2011 NICE guideline, 17 899 (55·6%) under the proposed 2019 NICE guideline, 20 388 (63·4%) under a blood pressure alone strategy, and 21 851 (67·9%) under the QRISK2 strategy (figure 1, appendix p 12). Rates of cardiovascular disease were highest in patients eligible under the QRISK2 strategy (16·9 per 1000 person-years), followed by the 2011 (15·2) and the 2019 (14·9) NICE guidances, and were considerably lower under the strategy of blood pressure alone (11·4).

Compared with the 2011 NICE guideline, eligibility was higher and more events would be prevented under all other strategies, but efficiency varied. Using blood pressure alone to determine treatment eligibility led to an additional 5 037 504 patients in the UK being eligible for treatment compared with the NICE 2011 strategy. We estimated that, over 10 years, this strategy would avoid an additional 68 371 cardiovascular events compared with the 2011 NICE strategy. Under a blood pressure alone strategy, more patients would be treated for 10 years per event avoided (NNT=38), compared with under the 2011 NICE guideline (NNT=28; table 2). We found that more cardiovascular events would be avoided with the QRISK2 strategy, along with a lower NNT, than with the 2011 NICE guideline, making QRISK2 the most efficient strategy (table 2).

**Table 2:** Patients eligible for blood pressure-lowering treatment under each strategy, predicted events over 10 years, events that would be avoided with treatment for 10 years, patients treated for 10 years per event avoided, and additional eligible patients and events avoided of each strategy compared with those of the 2011 NICE guideline

	**Estimated eligibility in UK population aged 30–79 years (n=29 344 080)**	Predicted outcomes over 10 years among eligible patients if all patients were untreated*	**Events that could be avoided with treatment of all eligible patients**†	**Patients needed to treat for 10 years to avoid one event**	**Additional patients eligible compared with 2011 NICE guideline (n=6 527 112)**	**Additional events avoided compared with 2011 NICE guideline (n=233 152)**
Blood pressure threshold	11 564 616 (39·4%)	1 507 615	301 523	38	5 037 504 (77·2%)	68 371 (29·3%)
2011 NICE guideline	6 527 112 (22·2%)	1 165 760	233 152	28	..	..
Proposed 2019 NICE guideline	7 858 296 (26·8%)	1 351 164	270 233	29	1 331 184 (20·4%)	37 081 (15·9%)
QRISK2 10-year risk threshold	8 588 160 (29·3%)	1 614 606	322 921	27	2 061 048 (31·6%)	89 769 (38·5%)

Data are n (%), unless otherwise specified. The four strategies compared are the following: blood pressure threshold of 140/90 mm Hg or higher, 2011 NICE guideline, proposed 2019 NICE guideline, and QRISK2 10-year risk of 10% or higher. NICE=National Institute for Health and Care Excellence.

* Estimated as predicted outcomes in treated and untreated patients; predicted events in untreated patients were calculated as event rate × number of eligible patients × 10; predicted events in treated patients were calculated as event rate × number of eligible patients × 10 × 1·25 (inflating number of outcomes in treated patients by 20%).

† 20% of predicted outcomes.

**Figure 3:**
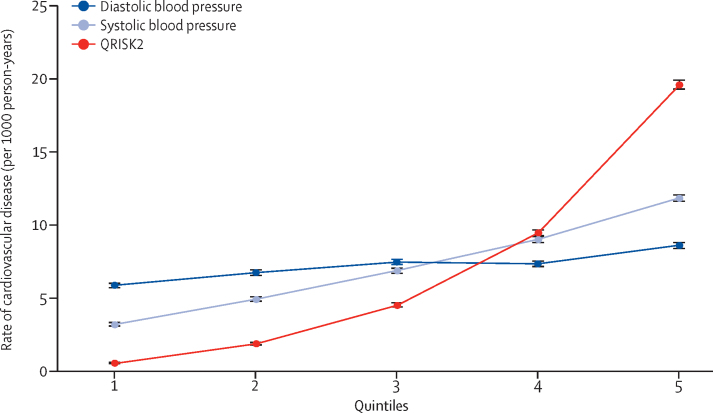
Quintiles of QRISK2 10-year risk score, systolic blood pressure, and diastolic blood pressure at enrolment versus the rate of cardiovascular disease during follow-up Error bars are 95% CIs. Quintile values for QRISK2: (1) 0·04–0·98%, (2) 0·98–2·82%, (3) 2·82–6·61%, (4) 6·61–14·23%, and (5) 14·23–95·70%. Quintiles for systolic blood pressure (in mm Hg): (1) 40–116, (2) 117–125, (3) 126–132, (4) 133–140, and (5) 141–260. Quintiles for diastolic blood pressure (in mm Hg): (1) 20–70, (2) 71–77, (3) 78–80, (4) 81–86, and (5) 87–192.

In the secondary analyses assessing groups of patients split by quintiles of QRISK2 score, systolic blood pressure, and diastolic blood pressure, we observed that the gradient of the rate of cardiovascular disease was steepest when the cohort was split by quintiles of QRISK2 score (figure 3). Rates according to commonly used categories of QRISK2 score, systolic blood pressure, and diastolic blood pressure also showed that QRISK2 score was more strongly associated with future cardiovascular disease events than either systolic blood pressure or diastolic blood pressure (appendix p 14).

In the secondary analyses, we also stratified rates of cardiovascular disease according to use of blood pressure-lowering treatment at enrolment. Among patients eligible for treatment under the 2011 NICE guideline, 70% were receiving treatment at enrolment and approximately half of those had blood pressure lower than the target of 140/90 mm Hg (appendix p 7). Approximately 40% of patients eligible under the other three strategies were treated at enrolment. Among both treated and untreated patients, the use of QRISK2 scores of 10% or higher was more discriminatory than the use of the 2011 NICE guideline for predicting future cardiovascular burden (appendix p 15). The distribution of blood pressures among patients receiving blood pressure-lowering treatment at enrolment showed that the majority of these patients had systolic blood pressure higher than 120 mm Hg (appendix p 16).

We did several post-hoc sensitivity analyses. We estimated that NNT for 5 years to prevent one event were greater than those calculated at 10 years for all strategies, but had the same between-strategy patterns as the main analysis (appendix p 9). Restricting our outcome to acute cardiovascular disease also showed the same pattern as the main analysis (appendix p 10). With the 2011 NICE guideline, NNT for 10 years was 55 patients, with the proposed 2019 NICE guideline was 56, with a QRISK2 strategy was 51, and with blood pressure alone was 74. The QRISK2 strategy was most discriminative in predicting haemorrhagic stroke, with the highest rate found in patients eligible under the QRISK2 strategy and the lowest rate in patients who were ineligible for treatment under the same strategy (appendix p 18).

## Discussion

Our contemporary cohort study of more than 1·2 million patients from primary care in the UK describes eligibility for blood pressure-lowering treatment and subsequent cardiovascular burden on the basis of four different strategies. A strategy based on blood pressure alone or the 2019 NICE guideline would recommend far more patients for treatment and would prevent more cardiovascular outcomes, but with less efficiency in terms of NNT compared with that of the 2011 NICE guideline. However, a strategy based on a QRISK2 10-year cardiovascular risk threshold of 10% (akin to statin guidelines in the UK) would result in a net increase in patients eligible for treatment compared with that of the 2011 NICE guideline, would prevent over a third more cardiovascular events, and would be at least as, or perhaps slightly more, efficient. In the UK, scaling up the results from this analysis suggested that a strategy based on cardiovascular risk for blood pressure-lowering treatment could prevent an additional 89 769 cardiovascular events over 10 years, with the potential for many more globally if guidance was based on absolute risk.

Our findings suggest that substantial numbers of at-risk patients are being overlooked for treatment because guidance is too focused on blood pressure. The patients in our cohort who would gain eligibility under a QRISK2 of 10% or higher (roughly 13% of all patients without cardiovascular disease) had a 10-year cardiovascular risk at least equivalent to that of patients currently eligible for treatment and, therefore, have as much or more to gain as those currently being treated. By definition, patients who lost eligibility under a QRISK2 strategy had a low QRISK2 score (<10%), and this was reflected in the lower cardiovascular event rates of these patients. More than 60% of patients who would lose eligibility under a QRISK2 strategy were using blood pressure-lowering medication and thus, might be overtreated by existing guidance. Our analysis showed that an absolute cardiovascular risk score is more predictive of future cardiovascular burden than systolic blood pressure or diastolic blood pressure. The 2011 NICE guideline incorporates an element of absolute risk by targeting patients at high risk (≥20%), which the proposed 2019 guidance lowers to 10% or higher, but only if they have blood pressure of 140/90 mm Hg or higher.

Large systematic reviews^1^, ^7^ have shown that patients with high cardiovascular risk would benefit considerably from treatment even at blood pressures lower than 140/90 mm Hg. A 2018 systematic review and meta-analysis^21^ contradicted this finding. However, the validity of this review is uncertain because it included heterogeneous studies with very different patient characteristics and lengths of follow-up.^22^, ^23^ The results of our study show the potential effect of using a risk-based approach; therefore, the effectiveness of blood pressure-lowering drugs in preventing cardiovascular disease in patients with blood pressures lower than 140/90 mm Hg needs to be assessed.

Policy makers and clinicians should use the best available tools to predict a patient's subsequent risk, on the basis of all the available evidence. In our study, a QRISK2-based treatment threshold outperformed the 2011 and 2019 NICE guidelines in predicting cardiovascular disease and thus, is likely to be a superior tool for determining treatment eligibility. Because European guidelines^5^ are similar to those of the UK in recommending treatment to patients with hypertension and incorporating risk calculation, similar gains might be made by applying an entirely risk-based strategy to other European populations. The US guidelines^3^ expanded treatment eligibility in 2017, and a similar risk-based approach in the USA would reduce eligibility, but could also be more efficient.

Guidelines^24^ in New Zealand have recommended an absolute cardiovascular risk-based strategy for both blood pressure-lowering and lipid-lowering treatment for longer than a decade, and a similar absolute risk-based strategy for statins is recommended in the UK.^14^ Additionally, a growing body of international evidence^8^, ^25^, ^26^, ^27^, ^28^, ^29^, ^30^, ^31^ has shown that a risk-based strategy is superior to a blood pressure-based approach or a combination (blood pressure and risk) strategy in terms of cardiovascular disease prevention.

A risk-based strategy might also be cost-effective, with projected savings in an Australian study^26^, ^32^ greater than AUD$5·4 billion. Although our study did not incorporate an economic analysis, our results suggest that a risk-based approach to treatment is likely to be as cost-effective as the 2011 or 2019 NICE guidelines.

Importantly, drug treatment for blood pressure is only one component for reducing cardiovascular risk overall. We are not implying that immediate drug treatment should be recommended to patients when increased cardiovascular risk is first identified. Non-drug measures to reduce risk would remain an important component of any strategy.

Our study has several limitations. Our analyses did not investigate the potential harms of changing eligibility for blood pressure treatment. For example, not treating patients who have high blood pressures but have low absolute risk could lead to target organ damage,^33^ or treating patients with high risk and normal blood pressure could result in harmful blood pressure reductions. The evidence for such harms is unclear, particularly among patients with diabetes and older patients.^34^, ^35^, ^36^, ^37^

We assumed that the rate of cardiovascular disease occurring during follow-up (median 4·3 years) could be extrapolated to 10 years. Therefore, our absolute numbers and rates come with a degree of uncertainty, but our sensitivity analysis over 5 years showed that a shorter prediction period would lead to the same conclusion.

Restricting our cohort to patients who had a recorded blood pressure might have biased it towards a sicker population who visit their general practitioner more frequently. The true proportion of the UK population who is eligible for any strategy and the true rates of cardiovascular disease might therefore be lower than reported here. However, patients who do not visit their GP cannot be captured or treated under any strategy, and our comparisons were made in patients who can be reached with preventive measures in real-world clinical practice.

Our cohort was comprised of treated and untreated patients. Patients who were eligible for treatment under the 2011 NICE guideline were more likely to use blood pressure-lowering treatment, affecting the subsequent burden of cardiovascular disease for this strategy more than for other strategies. However, stratification by treatment showed that QRISK2 was superior to the 2011 NICE guideline in both treated and untreated groups. Our calculation of NNT assumed that all patients being treated were already receiving a treatment benefit of 20% reduction in events. However, only half of patients treated had a recently measured blood pressure lower than the UK target of 140/90 mm Hg. This suggests that even treated patients could benefit from further blood pressure lowering. Considering that very few patients in our cohort had systolic blood pressure lower than 120 mm Hg, there could be additional benefits from lowering systolic blood pressure below 120mm Hg.

Eligibility for each strategy was based on data collected in 2011. Secular trends in cardiovascular disease, risk factors, and treatment will affect eligibility and the rate of cardiovascular disease, which might be higher in our cohort than in a more contemporary cohort.

QRISK2 calculators are widely used in UK primary care. However, QRISK2 was developed on the basis of coronary and cerebrovascular outcomes. Other outcomes need to be considered in assessing a risk-based approach to treatment, including peripheral arterial disease, heart failure, and target organ damage. Our sensitivity analysis exploring haemorrhagic stroke showed that using QRISK2 to determine treatment eligibility was unlikely to lead to withdrawal of treatment in patients at risk of haemorrhagic stroke.

Our study had several strengths. Our analysis was based on data from UK primary care. The CPRD is broadly representative of the UK population and thus, we were able to extend our results to the UK population. Linkage to hospital and mortality data allowed more cardiovascular events to be captured than by using CPRD alone.^38^

Some error in blood pressure measurement is likely to exist but, importantly, this strengthens our argument for a risk-based strategy. Blood pressures recorded in these data are those on which clinicians make treatment decisions. If a patient has unusually raised clinic blood pressure measures, they might be considered for therapy under the current approach. Because blood pressure is just one component of absolute risk, its effect on absolute risk is small. Therefore, errors in measurement or out-of-date measures will have less effect on eligibility for treatment.

Our study shows the need for re-assessment of the 2011 and proposed 2019 NICE blood pressure treatment guidelines. A cardiovascular risk-based strategy would align with the lipid-lowering guideline and streamline provision of cardiovascular disease prevention, with the potential to prevent more cardiovascular disease and with more efficiency than current guidelines. Given the large numbers of patients who stand to benefit from a change in guideline, this work should be a priority for public health and cardiovascular disease prevention.

## Data sharing

Data were obtained from the CPRD. CPRD is a research service that provides primary care and linked data for public health research. CPRD data governance, and our own licence to use CPRD data, do not allow us to distribute or make available patient data directly to other parties. Researchers can apply for data access with CPRD and must have their study protocol approved by the Independent Scientific Advisory Committee for Medicines and Healthcare products Regulatory Agency database research.

## Footnotes

* Target organ damage includes hypertensive retinopathy, heart failure, and proteinuria (appendix).

† QRISK2 includes age, sex, ethnicity, blood pressure, deprivation score from linked data, diabetes, family history of coronary heart disease, atrial fibrillation, chronic kidney disease stage 4 or 5, cholesterol–high-density lipoprotein ratio, rheumatoid arthritis, use of blood pressure-lowering drugs, body-mass index, and smoking status.
