# Supplementary material for: Eligibility and subsequent burden of cardiovascular disease of four strategies for blood pressure-lowering treatment: a retrospective cohort study
Source: Lancet. 2019 Aug 24;394(10199):663–71. doi: 10.1016/S0140-6736(19)31359-5 (PMC6717081; doi:10.1016/S0140-6736(19)31359-5)
Supplement: Supplementary appendix [file mmc1.pdf]

THE LANCET

Supplementary appendix

This appendix formed part of the original submission and has been peer reviewed. We post it as supplied by the authors.

Supplement to: Herrett E, Gadd S, Jackson R, et al. Eligibility and subsequent burden of cardiovascular disease of four strategies for blood pressure-lowering treatment: a retrospective cohort study. *Lancet* 2019; published online July 25. http://dx.doi.org/10.1016/S0140-6736(19)31359-5.

Contents

Additional information 2

Additional Tables 3

Additional Figures 11

Code lists for use with CPRD, HES and ONS data 19

Medical code list for cardiovascular disease in CPRD 19

ICD10 code list for cardiovascular disease 48

OPCS code list for cardiovascular disease 54

Target organ damage Read codes 61

ISAC protocol 65

RECORD statement 79

Additional information

Calculation of Number of patients treated for ten years per event avoided

This metric was calculated in order to compare the efficiency of each strategy. For each strategy, it was calculated as:

$$\frac{\text{number of patients eligible for treatment}}{\text{number of events potentially avoided if all eligible patients were treated}}$$

Number of patients eligible for treatment
This was estimated earlier for each strategy, see main text.

Number of events potentially avoided if all eligible patients were treated
This was calculated for eligible patients based on their treatment status at cohort entry. For untreated patients, this was the number that we would avoid if they were treated, and treated patients, this was the number that had already been avoided by treatment.

For *untreated* eligible patients, the number that would be avoided under this strategy was estimated as 20% of the expected number of cardiovascular disease events over ten years, calculated from the crude rate among the untreated eligible patients. So for each strategy:
(crude rate among eligible untreated x 10 years x number of untreated patients x 0.2)
The 20% reduction in events was based on evidence that lowering SBP by 10mmHg with drugs reduces cardiovascular disease risk by 20% in all patients, over about 5 years[7]).

> *For example, calculations for the NICE strategy were as follows:*
> *In our cohort there were 81,562 untreated eligible patients for the NICE strategy, and the crude rate of CVD in this group was 15.42 per 1000 person years.*
> *Therefore under a scenario where all eligible patients were treated, there would be:*
> *15.42/1000 x 10 years x 81,562 x 0.2 = 2515.2 events avoided in the cohort over ten years.*

We assumed that *treated* patients were already receiving their 20% treatment benefit. Therefore, as a group, they were experiencing 80% of the events compared to a scenario in which they were not treated (so multiply by a factor of 100/80 to get events expected if *untreated*). Then, the number they avoided due to treatment for ten years was estimated as 20% of this. The overall calculation for this group is therefore:

> (crude rate among eligible treated x 10 years x number of treated patients x 100/80 x 0.2).
>
> *For example, calculations for the NICE strategy were as follows:*
> *In our cohort there were 190,401 treated eligible patients for the NICE strategy and the crude rate of CVD in this group was 15.1 per 1000 person years.*
> *Therefore under a scenario where all eligible patients were treated, there would be:*
> *15.12/1000 x 10 years x 190,401 x 100/80 x 0.2 = 7199.5 events avoided in the cohort over ten years.*

These two figures were summed to estimate total number of events potentially avoided due to treatment.

All estimates were scaled to the UK population. This was done by multiplying our estimates by 24. (Our original sample of CPRD data, before exclusion criteria were applied, was 2.7 million patients (see Appendix Figure 1), representing approximately $1/24^{th}$ of the UK population in 2011.)

> *So for our example calculation for the NICE strategy:*
> *Events avoided among untreated patients: 2515.2 x 24 = 60,364 events avoided in the UK over ten years; and among treated patients: 7199.5 x 24 = 172,788 events avoided in the UK over ten years.*
> *Therefore the number potentially avoided due to treatment is 60,364 + 172,788 = 233,152.*

Additional Tables

Appendix Table 1. Full demographic and risk factor characteristics at cohort entry for the whole cohort, and for patients eligible and ineligible for blood pressure lowering treatment under each strategy (blood pressure ≥140/90mmHg, current NICE guidelines, QRISK2 ten year risk ≥10%).

	Whole cohort		Eligibility strategy 1, Blood pressure ≥140/90mmHg				Eligibility strategy 2, NICE guidelines 2011				Eligibility strategy 3, NICE guidelines 2019				Eligibility strategy 4, QRISK2 ten year risk ≥10%			
			Eligible		Ineligible		Eligible		Ineligible		Eligible		Ineligible		Eligible		Ineligible	
**N**	1,222,670	(100)	481,859	(39.4)	740,811	(60.6)	271,963	(22.2)	950,707	(77.8)	327,429	(26.8)	895,241	(73.2)	357,840	(29.3)	864,830	(70.7)
**Time in follow-up in years, median (IQR)**	4.3	(2.5-5.2)	4.3	(2.5-5.2)	4.3	(2.5-5.2)	4.2	(2.5-5.2)	4.3	(2.5-5.2)	4	(2.5-5.2)	4.3	(2.5-5.2)	3.9	(2.4-5.2)	4.3	(2.6-5.2)
**Gender, n (%)**																		
Male	530,618	(43.4)	242,423	(50.3)	288,195	(38.9)	134,958	(49.6)	395,660	(41.6)	168,143	(51.4)	362,475	(40.5)	209,882	(58.7)	320,736	(37.1)
Female	692,052	(56.6)	239,436	(49.7)	452,616	(61.1)	137,005	(50.4)	555,047	(58.4)	159,286	(48.6)	532,766	(59.5)	147,958	(41.3)	544,094	(62.9)
**Age group at entry, n(%)**																		
30-39	251,294	(20.6)	38,399	(8)	212,895	(28.7)	9,270	(3.4)	242,024	(25.5)	9,293	(2.8)	242,001	(27)	709	(0.2)	250,585	(29)
40-49	317,280	(25.9)	86,909	(18)	230,371	(31.1)	31,623	(11.6)	285,657	(30)	32,737	(10)	284,543	(31.8)	9,397	(2.6)	307,883	(35.6)
50-59	281,786	(23)	121,185	(25.1)	160,601	(21.7)	58,146	(21.4)	223,640	(23.5)	69,414	(21.2)	212,372	(23.7)	55,263	(15.4)	226,523	(26.2)
60-69	238,774	(19.5)	139,937	(29)	98,837	(13.3)	87,968	(32.3)	150,806	(15.9)	120,577	(36.8)	118,197	(13.2)	159,184	(44.5)	79,590	(9.2)
70-79	133,536	(10.9)	95,429	(19.8)	38,107	(5.1)	84,956	(31.2)	48,580	(5.1)	95,408	(29.1)	38,128	(4.3)	133,287	(37.2)	249	(0)
**Ethnicity, n (%)**																		
White or NS	949,614	(77.7)	381,609	(79.2)	568,005	(76.7)	218,171	(80.2)	731,443	(76.9)	262,905	(80.3)	686,709	(76.7)	292,506	(81.7)	657,108	(76)
Indian	15,289	(1.3)	4,299	(0.9)	10,990	(1.5)	2,537	(0.9)	12,752	(1.3)	2,922	(0.9)	12,367	(1.4)	3,618	(1)	11,671	(1.3)
Pakistani	5,565	(0.5)	1,267	(0.3)	4,298	(0.6)	767	(0.3)	4,798	(0.5)	889	(0.3)	4,676	(0.5)	1,369	(0.4)	4,196	(0.5)
Bangladeshi	1,862	(0.2)	385	(0.1)	1,477	(0.2)	251	(0.1)	1,611	(0.2)	279	(0.1)	1,583	(0.2)	407	(0.1)	1,455	(0.2)
Other Asian	10,541	(0.9)	2,679	(0.6)	7,862	(1.1)	1,385	(0.5)	9,156	(1)	1,620	(0.5)	8,921	(1)	1,822	(0.5)	8,719	(1)
Black Caribbean	6,168	(0.5)	2,543	(0.5)	3,625	(0.5)	1,530	(0.6)	4,638	(0.5)	1,677	(0.5)	4,491	(0.5)	1,289	(0.4)	4,879	(0.6)
Black African	10,591	(0.9)	3,289	(0.7)	7,302	(1)	1,578	(0.6)	9,013	(0.9)	1,676	(0.5)	8,915	(1)	719	(0.2)	9,872	(1.1)
Chinese	2,922	(0.2)	594	(0.1)	2,328	(0.3)	290	(0.1)	2,632	(0.3)	326	(0.1)	2,596	(0.3)	267	(0.1)	2,655	(0.3)
Other ethnic group	220,118	(18)	85,194	(17.7)	134,924	(18.2)	45,454	(16.7)	174,664	(18.4)	55,135	(16.8)	164,983	(18.4)	55,843	(15.6)	164,275	(19)
**QRISK2 score, n (%)**																		
<10%	864,830	(70.7)	235,788	(48.9)	629,042	(84.9)	81,358	(29.9)	783,472	(82.4)	81,358	(24.8)	783,472	(87.5)	0	(0)	864,830	(100)
10-19%	216,349	(17.7)	136,578	(28.3)	79,771	(10.8)	81,112	(29.8)	135,237	(14.2)	136,578	(41.7)	79,771	(8.9)	216,349	(60.5)	0	(0)
20-29%	95,167	(7.8)	71,019	(14.7)	24,148	(3.3)	71,019	(26.1)	24,148	(2.5)	71,019	(21.7)	24,148	(2.7)	95,167	(26.6)	0	(0)
>=30%	46,324	(3.8)	38,474	(8)	7,850	(1.1)	38,474	(14.1)	7,850	(0.8)	38,474	(11.8)	7,850	(0.9)	46,324	(12.9)	0	(0)
**Townsend quintile, n (%)**																		
1	302,731	(24.8)	116,036	(24.1)	186,695	(25.2)	61,221	(22.5)	241,510	(25.4)	75,007	(22.9)	227,724	(25.4)	80,653	(22.5)	222,078	(25.7)
2	207,932	(17)	82,712	(17.2)	125,220	(16.9)	44,987	(16.5)	162,945	(17.1)	55,176	(16.9)	152,756	(17.1)	60,879	(17)	147,053	(17)
3	250,157	(20.5)	96,055	(19.9)	154,102	(20.8)	51,991	(19.1)	198,166	(20.8)	63,341	(19.3)	186,816	(20.9)	68,831	(19.2)	181,326	(21)
4	224,100	(18.3)	100,524	(20.9)	123,576	(16.7)	64,615	(23.8)	159,485	(16.8)	75,032	(22.9)	149,068	(16.7)	80,245	(22.4)	143,855	(16.6)
5	237,750	(19.4)	86,532	(18)	151,218	(20.4)	49,149	(18.1)	188,601	(19.8)	58,873	(18)	178,877	(20)	67,232	(18.8)	170,518	(19.7)

*closest measure prior to cohort entry on 1st January 2011

**closest measure prior to cohort entry on 1st January 2011, and either systolic or diastolic blood pressure met criteria

Continued…

Appendix Table 1 continued

	Whole cohort	Eligibility strategy 1, Blood pressure ≥140/90mmHg	Eligibility strategy 2, NICE guidelines 2011	Eligibility strategy 3, NICE guidelines 2019	Eligibility strategy 4, QRISK2 ten year risk ≥10%

			Eligible		Ineligible		Eligible		Ineligible		Eligible		Ineligible		Eligible		Ineligible	
**Family history cardiovascular disease, n (%)**	97,120	(7.9)	38,868	(8.1)	58,252	(7.9)	21,777	(8)	75,343	(7.9)	27,826	(8.5)	69,294	(7.7)	35,154	(9.8)	61,966	(7.2)
**Diabetes, n (%)**	137,419	(11.2)	76,897	(16)	60,522	(8.2)	76,897	(28.3)	60,522	(6.4)	76,897	(23.5)	60,522	(6.8)	80,058	(22.4)	57,361	(6.6)
**Smoking status, n (%)**																		
Non smoker	688,168	(56.3)	266,013	(55.2)	422,155	(57)	145,484	(53.5)	542,684	(57.1)	172,148	(52.6)	516,020	(57.6)	167,630	(46.8)	520,538	(60.2)
Ex smoker	290,181	(23.7)	131,345	(27.3)	158,836	(21.4)	82,322	(30.3)	207,859	(21.9)	99,325	(30.3)	190,856	(21.3)	116,105	(32.4)	174,076	(20.1)
Light smoker	11,025	(0.9)	3,245	(0.7)	7,780	(1.1)	1,553	(0.6)	9,472	(1)	1,970	(0.6)	9,055	(1)	2,480	(0.7)	8,545	(1)
Moderate smoker	222,021	(18.2)	77,239	(16)	144,782	(19.5)	41,091	(15.1)	180,930	(19)	51,923	(15.9)	170,098	(19)	69,039	(19.3)	152,982	(17.7)
Heavy smoker	4,210	(0.3)	1,605	(0.3)	2,605	(0.4)	849	(0.3)	3,361	(0.4)	1,106	(0.3)	3,104	(0.3)	1,553	(0.4)	2,657	(0.3)
Missing	7,065	(0.6)	2,412	(0.5)	4,653	(0.6)	664	(0.2)	6,401	(0.7)	957	(0.3)	6,108	(0.7)	1,033	(0.3)	6,032	(0.7)
**BMI, median (IQR)**	26.6	(23.8-30.3)	28	(25.1-32)	25.7	(23.2-29)	28.6	(25.5-32.7)	26.1	(23.5-29.5)	28	(25.3-32.2)	26	(23.4-29.4)	27.6	(24.8-31.1)	26.2	(23.5-29.9)
Missing	149,054	(12.2)	44,283	(9.2)	104,771	(14.1)	18,327	(6.7)	130,727	(13.8)	24,553	(7.5)	124,501	(13.9)	31,051	(8.7)	118,003	(13.6)
**Total: HDL cholesterol ratio, mean (SD)**	3.9	(1.2)	3.9	(1.2)	3.9	(1.2)	3.9	(1.2)	3.9	(1.2)	3.9	(1.2)	3.9	(1.2)	4	(1.3)	3.8	(1.2)
Missing	750,733	(61.4)	228,675	(47.5)	522,058	(70.5)	99,156	(36.5)	651,577	(68.5)	126,842	(38.7)	623,891	(69.7)	144,303	(40.3)	606,430	(70.1)
**Systolic blood pressure, mmHg, n (%)***																		
<110	100,345	(8.2)	2,876	(0.6)	97,469	(13.2)	2,348	(0.9)	97,997	(10.3)	2,489	(0.8)	97,856	(10.9)	6,660	(1.9)	93,685	(10.8)
110-119	187,402	(15.3)	13,123	(2.7)	174,279	(23.5)	9,929	(3.7)	177,473	(18.7)	10,610	(3.2)	176,792	(19.7)	21,811	(6.1)	165,591	(19.1)
120-139	603,024	(49.3)	133,961	(27.8)	469,063	(63.3)	92,186	(33.9)	510,838	(53.7)	100,308	(30.6)	502,716	(56.2)	162,640	(45.5)	440,384	(50.9)
140-159	289,745	(23.7)	289,745	(60.1)	0	(0)	125,346	(46.1)	164,399	(17.3)	171,868	(52.5)	117,877	(13.2)	139,895	(39.1)	149,850	(17.3)
160-179	35,618	(2.9)	35,618	(7.4)	0	(0)	35,618	(13.1)	0	(0)	35,618	(10.9)	0	(0)	21,939	(6.1)	13,679	(1.6)
≥180	6,536	(0.5)	6,536	(1.4)	0	(0)	6,536	(2.4)	0	(0)	6,536	(2)	0	(0)	4,895	(1.4)	1,641	(0.2)
**Systolic blood pressure, mean (SD)***	129.2	(15.6)	141.9	(12.9)	120.9	(11)	142.4	(15.6)	125.4	(13.4)	142.5	(14.6)	124	(13)	137.5	(14.8)	125.8	(14.7)
**Diastolic blood pressure, mean (SD)***	78.2	(9.5)	83.4	(9.5)	74.9	(7.9)	82.5	(10.7)	77	(8.8)	82.5	(10.2)	77	(8.7)	79.3	(9.3)	77.8	(9.6)
**Using blood pressure lowering medication, n (%)**	223,465	(18.3)	213,046	(44.2)	10,419	(1.4)	190,401	(70)	33,064	(3.5)	200,214	(61.1)	23,251	(2.6)	153,586	(42.9)	69,879	(8.1)
**Using statins, n (%)**	124,380	(10.2)	96,703	(20.1)	27,677	(3.7)	83,808	(30.8)	40,572	(4.3)	91,206	(27.9)	33,174	(3.7)	95,170	(26.6)	29,210	(3.4)
**Rheumatoid arthritis, n (%)**	11,233	(0.9)	5,889	(1.2)	5,344	(0.7)	4,460	(1.6)	6,773	(0.7)	5,124	(1.6)	6,109	(0.7)	6,553	(1.8)	4,680	(0.5)
**Atrial fibrillation, n (%)**	12,712	(1)	8,929	(1.9)	3,783	(0.5)	8,127	(3)	4,585	(0.5)	8,736	(2.7)	3,976	(0.4)	11,646	(3.3)	1,066	(0.1)
**Renal disease, n (%)**	49,744	(4.1)	36,245	(7.5)	13,499	(1.8)	36,245	(13.3)	13,499	(1.4)	36,245	(11.1)	13,499	(1.5)	36,671	(10.2)	13,073	(1.5)
**Proteinuria, n (%)**	9,713	(0.8)	5,581	(1.2)	4,132	(0.6)	5,581	(2.1)	4,132	(0.4)	5,581	(1.7)	4,132	(0.5)	4,813	(1.3)	4,900	(0.6)
**Hypertensive retinopathy, n (%)**	544	(0)	483	(0.1)	61	(0)	483	(0.2)	61	(0)	483	(0.1)	61	(0)	377	(0.1)	167	(0)

*closest measure prior to cohort entry on 1st January 2011

**closest measure prior to cohort entry on 1st January 2011, and either systolic or diastolic blood pressure met criteria

Appendix table 2. Number and proportion of patients in the UK who would gain and lose eligibility for blood pressure lowering treatment under each strategy (blood pressure ≥140/90mmHg, NICE guidelines 2019, QRISK2 ten year risk ≥10%) compared to the NICE 2011 guideline.

	n (%) in the UK population that would lose eligibility for treatment		n (%) in the UK population that would gain eligibility for treatment		Total n (%) patients in the UK population whose treatment eligibility would change		Net increase in eligibility in the UK population, n (%)	
**Blood pressure ≥140/90mmHg**	0	(0)	5,037,504	(17.2)	5,037,504	(17.2)	5,037,504	(17.2)
**NICE guidelines 2019**	0	(0)	1,331,184	(4.5)	1,331,184	(4.5)	1,331,184	(4.5)
**QRISK2 ten year risk ≥10%**	1,952,592	(6.7)	4,013,640	(13.7)	5,966,232	(20.3)	2,061,048	(7)

Appendix Table 3. Treatment and control of blood pressure to below 140/90 mmHg in patients eligible for treatment under each strategy.

	Whole cohort, n (%)		Strategy 1, Blood pressure ≥140/90mmHg, n (%)		Strategy 2, NICE guidelines 2011, n (%)		Strategy 2, NICE guidelines 2019, n (%)		Strategy 4, QRISK2 ten year risk ≥10%, n (%)	
			Patients eligible for blood pressure lowering treatment under:							
**Treated at cohort entry**	223,465	(18.3)	213,046	(44.2)	190,401	(70)	200,214	(61.1)	153,586	(43)
Blood pressure <140/90mmHg	*117,030*	*(9.6)*	*106,611*	*(22.1)*	*93,132*	*(34.2)*	*98,654*	*(30.1)*	*74,996*	*(21)*
Blood pressure ≥140/90mmHg	*106,435*	*(8.7)*	*106,435*	*(22.1)*	*97,269*	*(35.8)*	*101,560*	*(31)*	*78,590*	*(22)*
**Untreated at cohort entry**	999,205	(81.7)	268,813	(55.8)	81,562	(30)	127,215	(38.9)	204,254	(57.1)
Blood pressure <140/90mmHg	*730,392*	*(59.7)*	-	*(0)*	-	*(0)*	-	*(0)*	*108,434*	*(30.3)*
Blood pressure ≥140/90mmHg	*268,813*	*(22)*	*268,813*	*(55.8)*	*81,562*	*(30)*	*127,215*	*(38.9)*	*95,820*	*(26.8)*

Appendix Table 4. Demographic and risk factor characteristics of patients who are eligible for treatment under NICE guidelines 2011, and who lost and did not lose their treatment eligibility when a risk-based strategy was applied.

	Lost eligibility		Did not lose eligibility	
**N**	81,358	(29.9)	190,605	(70.1)
**Time in follow-up in years, median (IQR)**	4.4	(2.6-5.2)	3.9	(2.3-5.2)
**Gender, n (%)**				
Male	28,901	(35.5)	106,057	(55.6)
Female	52,457	(64.5)	84,548	(44.4)
**Age group at entry**				
30-39	8,769	(10.8)	501	(0.3)
40-49	26,091	(32.1)	5,532	(2.9)
50-59	32,006	(39.3)	26,140	(13.7)
60-69	14,472	(17.8)	73,496	(38.6)
70-79	20	(0)	84,936	(44.6)
**Ethnicity, n (%)**				
White or NS	61,797	(76)	156,374	(82)
Indian	649	(0.8)	1,888	(1)
Pakistani	162	(0.2)	605	(0.3)
Bangladeshi	67	(0.1)	184	(0.1)
Other Asian	477	(0.6)	908	(0.5)
Black Caribbean	682	(0.8)	848	(0.4)
Black African	1,138	(1.4)	440	(0.2)
Chinese	161	(0.2)	129	(0.1)
Other ethnic group	16,225	(19.9)	29,229	(15.3)
**QRISK2 score**				
<10%	81,358	(100)	-	(0)
10-19%	-	(0)	81,112	(42.6)
20-29%	-	(0)	71,019	(37.3)
>=30%	-	(0)	38,474	(20.2)
**Townsend quintile**				
1	20,455	(25.1)	40,766	(21.4)
2	13,676	(16.8)	31,311	(16.4)
3	16,251	(20)	35,740	(18.8)
4	16,490	(20.3)	48,125	(25.2)
5	14,486	(17.8)	34,663	(18.2)
**Family history cardiovascular disease**	5,458	(6.7)	16,319	(8.6)
**Diabetes**	17382	(21.4)	59515	(31.2)
**Smoking status**				
Non smoker	53,171	(65.4)	92,313	(48.4)
Ex smoker	17,378	(21.4)	64,944	(34.1)
Light smoker	433	(0.5)	1,120	(0.6)
Moderate smoker	9,868	(12.1)	31,223	(16.4)
Heavy smoker	177	(0.2)	672	(0.4)
Missing	331	(0.4)	333	(0.2)
**BMI, median (IQR)**	29	(25.7-33.5)	28.4	(25.5-32.3)
Missing	6,405	(7.9)	11,922	(6.3)
**Total: HDL cholesterol ratio, mean SD**	3.8	(1.1)	3.9	(1.2)
Missing	35,533	(43.7)	63,623	(33.4)
**Systolic blood pressure**				
<110	829	(1)	1,519	(0.8)
110-119	3,686	(4.5)	6,243	(3.3)
120-139	29,550	(36.3)	62,636	(32.9)
140-159	31,973	(39.3)	93,373	(49)
160-179	13,679	(16.8)	21,939	(11.5)
≥180	1,641	(2)	4,895	(2.6)
**Systolic blood pressure**	142	(16.2)	143	(15.3)
**Diastolic blood pressure**	87	(10.9)	81	(10)
**Using blood pressure lowering medication**	49,963	(61.4)	140,438	(73.7)
**Using statins**	11,952	(14.7)	71,856	(37.7)
**Rheumatoid arthritis**	707	(0.9)	3,753	(2)
**Atrial fibrillation**	59	(0.1)	8,068	(4.2)
**Renal disease**	6,373	(7.8)	29,872	(15.7)
**Proteinuria**	1,697	(2.1)	3,884	(2)
**Hypertensive retinopathy**	132	(0.2)	351	(0.2)
**Cardiovascular disease during follow-up**	2,038	(2.5)	13,098	(6.9)

Appendix Table 5. Number of patients eligible for blood pressure lowering treatment under each strategy, predicted events over five years, number of events that would be avoided with treatment for five years, number treated for five years per event avoided, additional patients who are eligible compared to NICE guidelines 2011, and additional events avoided compared to NICE guidelines 2011.

	Estimated eligibility in UK population aged 30-79, n(%)		N predicted outcomes among eligible patients over 5 years if all patients were untreated*	N events that could be avoided with treatment of all eligible patients**	Number of patients needed to treat for five years to avoid one event	Additional N (%) patients eligible compared to NICE 2011		Additional N (%) events avoided compared to NICE 2011	
**Blood pressure ≥140/90mmHg**	11,564,616	(39.4)	753,807	150761	77	5,037,504	(77.2)	34,186	(29.3)
**NICE guidelines 2011**	6,527,112	(22.2)	582,880	116576	56	-		-	
**NICE guidelines 2019**	7,858,296	(26.8)	675,582	135116	58	1,331,184	(20.4)	18,540	(15.9)
**QRISK2 ten year risk ≥10%**	8,588,160	(29.3)	807,303	161461	53	2,061,048	(31.6)	44,885	(38.5)

*Estimated as predicted outcomes in treated and untreated patients, where: untreated patients predicted events = event rate x number of eligible patients x 5; treated patients predicted events: event rate x number of eligible patients x 5 x 1.25 (inflating number of outcomes among treated by 20%)

**20% of predicted outcomes

Appendix Table 6. Number of patients eligible for blood pressure lowering treatment under each strategy, predicted acute CVD events (myocardial infarction and stroke) over ten years, number of acute CVD events that would be avoided with treatment for ten years, number treated for ten years per event avoided, additional patients who are eligible compared to NICE guidelines 2011, and additional acute CVD events avoided compared to NICE guidelines 2011.

	Estimated eligibility in UK population aged 30-79, n(%)		N predicted outcomes among eligible patients over 10 years if all patients were untreated*	N events that could be avoided with treatment of all eligible patients**	Number of patients needed to treat for ten years to avoid one event	Additional N (%) patients eligible compared to NICE 2011		Additional N (%) events avoided compared to NICE 2011	
**Blood pressure ≥140/90mmHg**	11,564,616	(39.4)	777,455	155491	74	5,037,504	(77.2)	36,239	(30.4)
**NICE guidelines 2011**	6,527,112	(22.2)	596,258	119252	55	-		-	
**NICE guidelines 2019**	7,858,296	(26.8)	695,987	139197	56	1,331,184	(20.4)	19,946	(16.7)
**QRISK2 ten year risk ≥10%**	8,588,160	(29.3)	848,736	169747	51	2,061,048	(31.6)	50,496	(42.3)

*Estimated as predicted outcomes in treated and untreated patients, where: untreated patients predicted events = event rate x number of eligible patients x 10; treated patients predicted events: event rate x number of eligible patients x 10 x 1.25 (inflating number of outcomes among treated by 20%)

**20% of predicted outcomes

Additional Figures

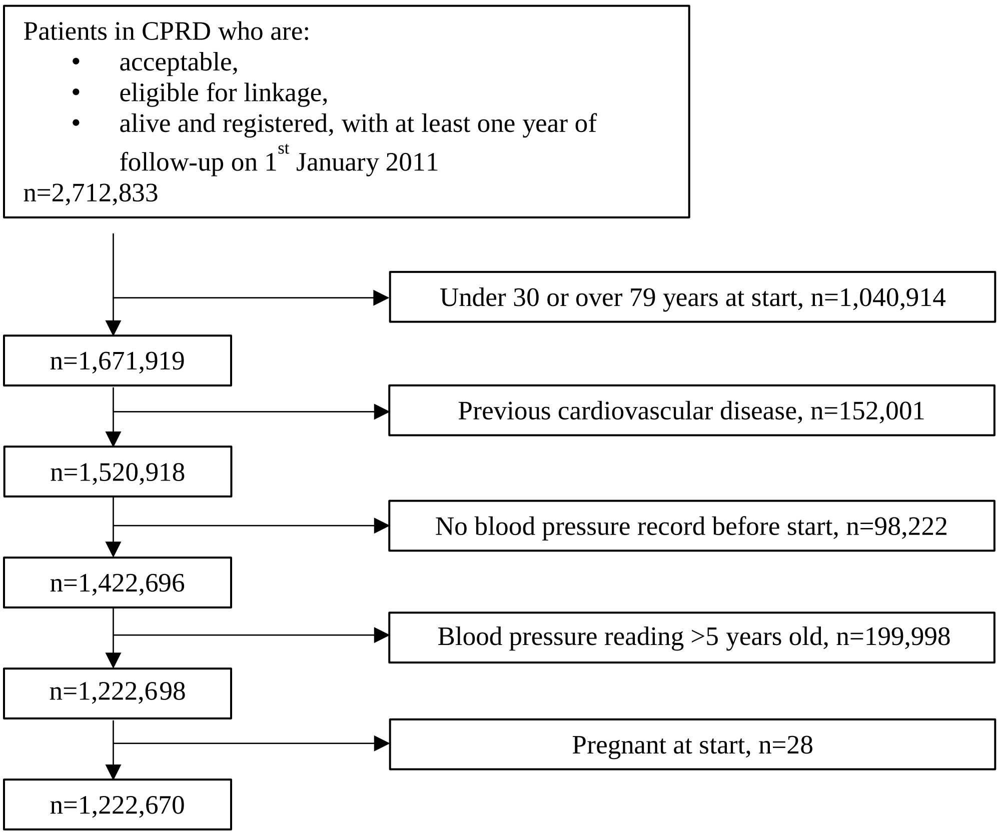

Appendix Figure 1. Cohort selection flowchart

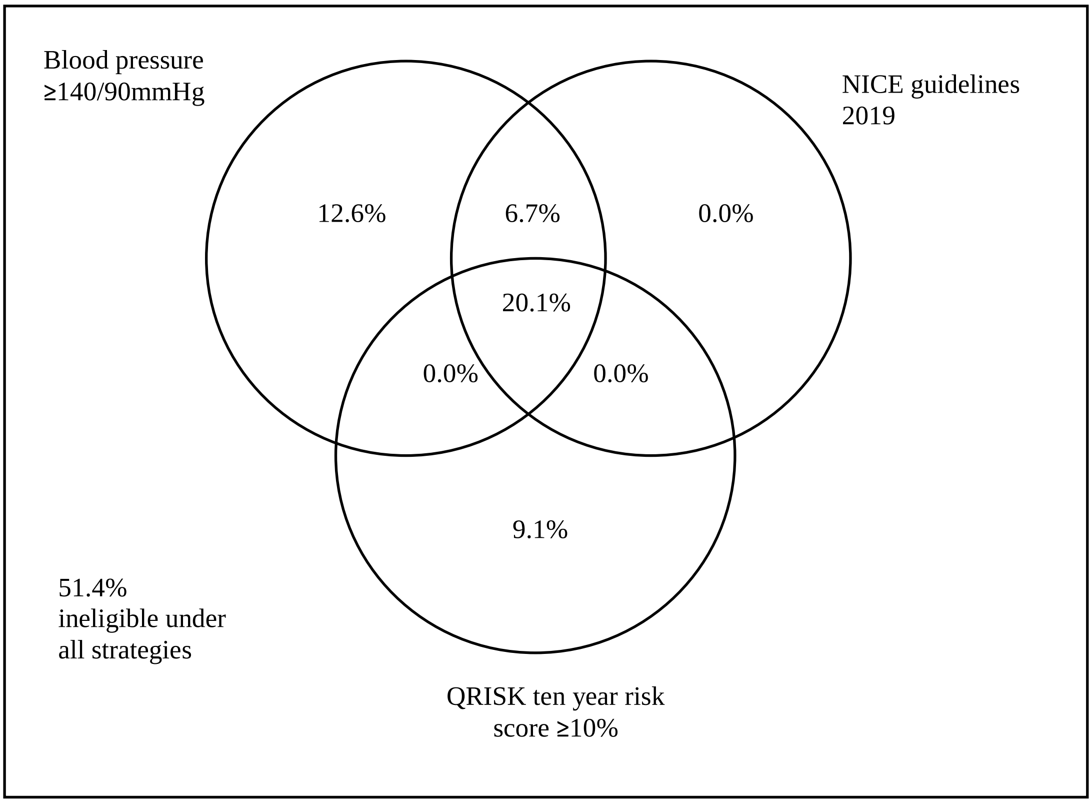

Appendix Figure 2. Comparison of eligibility for blood pressure lowering treatment based on three strategies ((i) blood pressure ≥140/90mmHg alone, (ii) NICE guidelines 2011, (iii) QRISK2 10 year cardiovascular disease risk ≥10%)

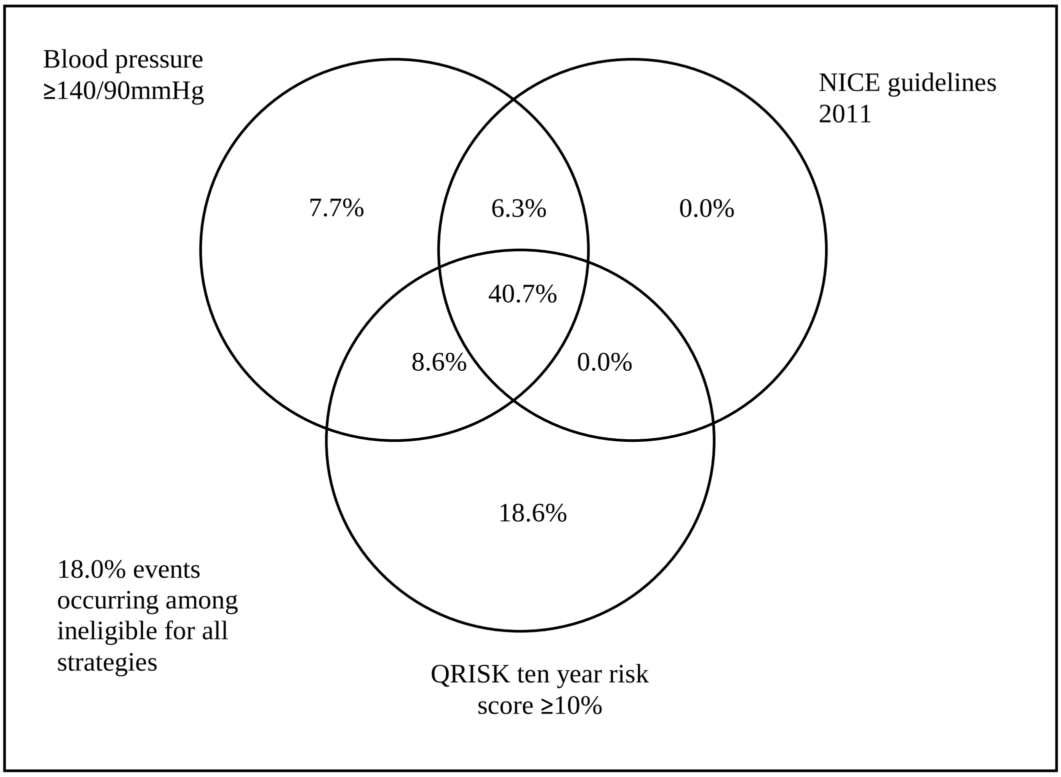

Appendix Figure 3. Proportion of all cardiovascular disease events (N=32,183) occurring according to baseline eligibility based on three strategies ((i) blood pressure ≥140/90mmHg alone, (ii) NICE 2011 guidelines, (iii) QRISK2 10 year cardiovascular disease risk ≥10%). Note: 18.0% of cardiovascular disease events occurred among patients who were not eligible under any strategy.

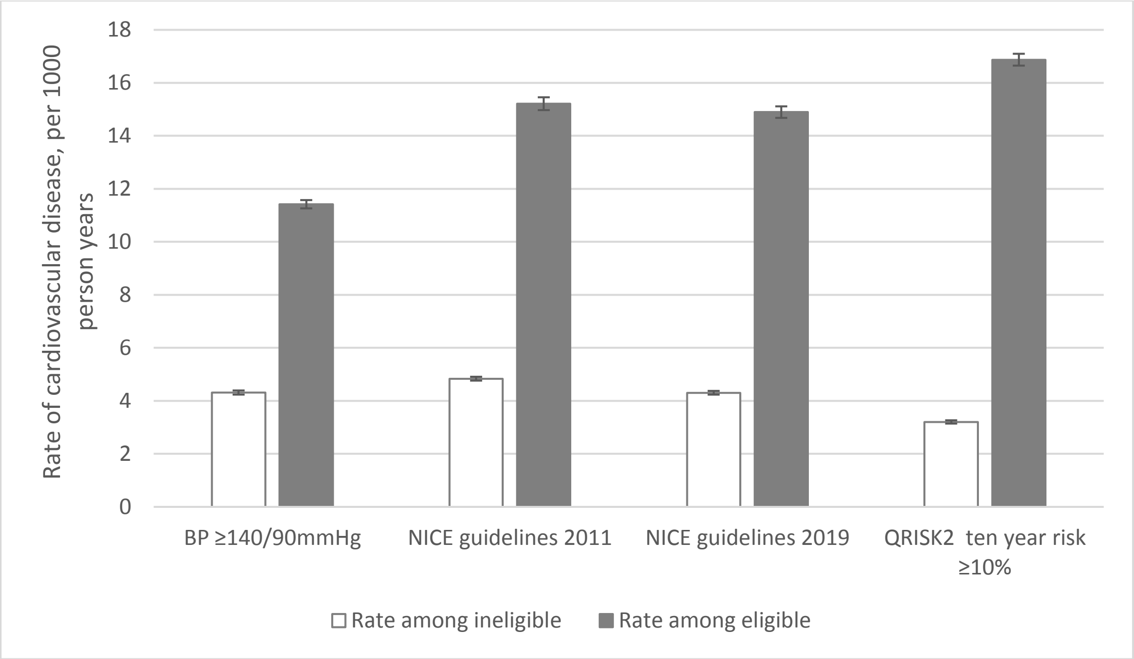

Appendix Figure 4. Rates of cardiovascular disease per 1000 person years among patients who were classified as eligible and ineligible for blood pressure lowering treatment under each treatment strategy (blood pressure ≥140/90mmHg, NICE 2011 guidelines, NICE 2019 guidelines, QRISK2 ten year risk ≥10%) with 95% confidence intervals. BP: blood pressure

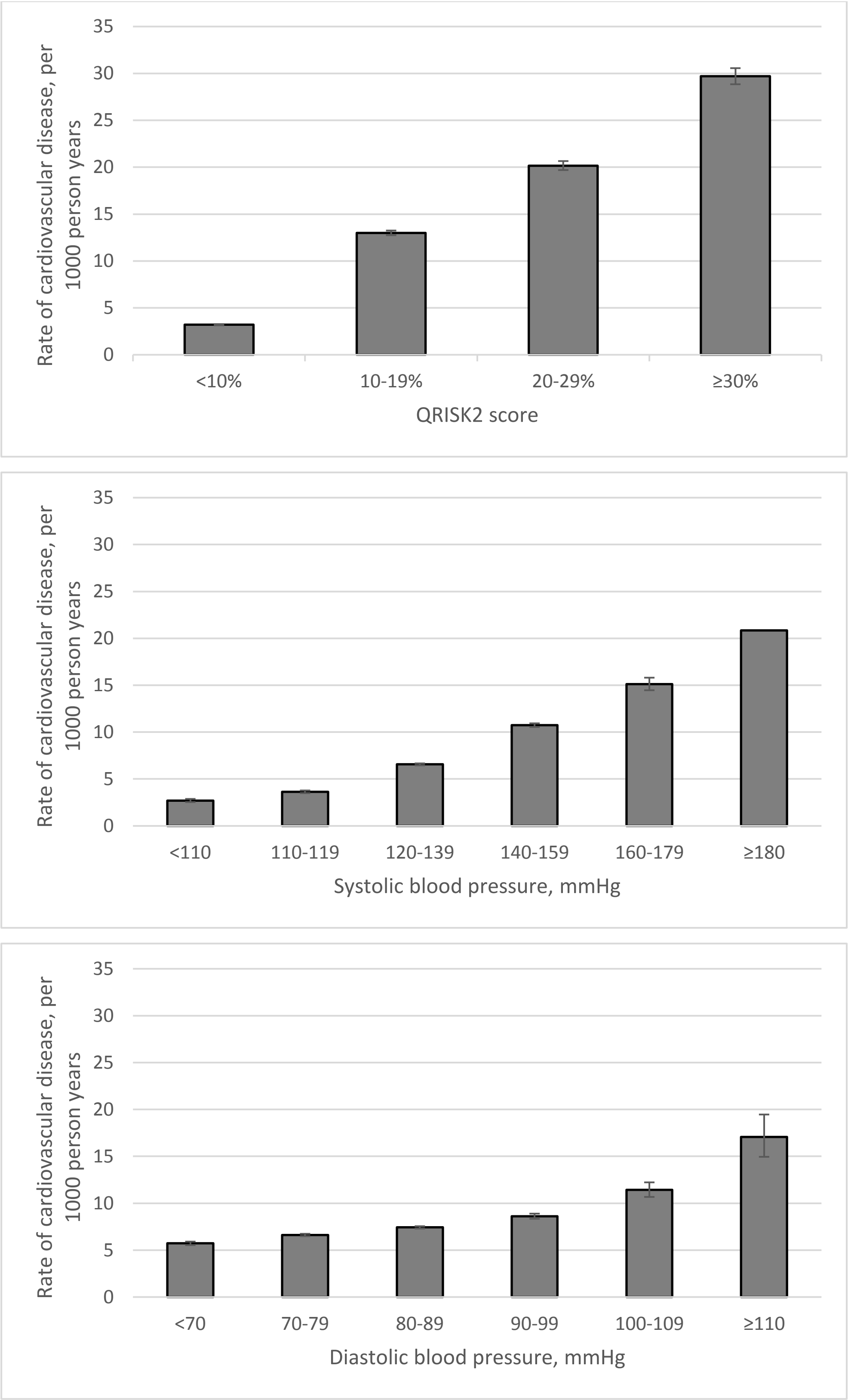

Appendix Figure 5. Rates of cardiovascular disease per 1000 person years, by groups of QRISK2 ten year risk score, systolic blood pressure and diastolic blood pressure (with 95% confidence intervals).

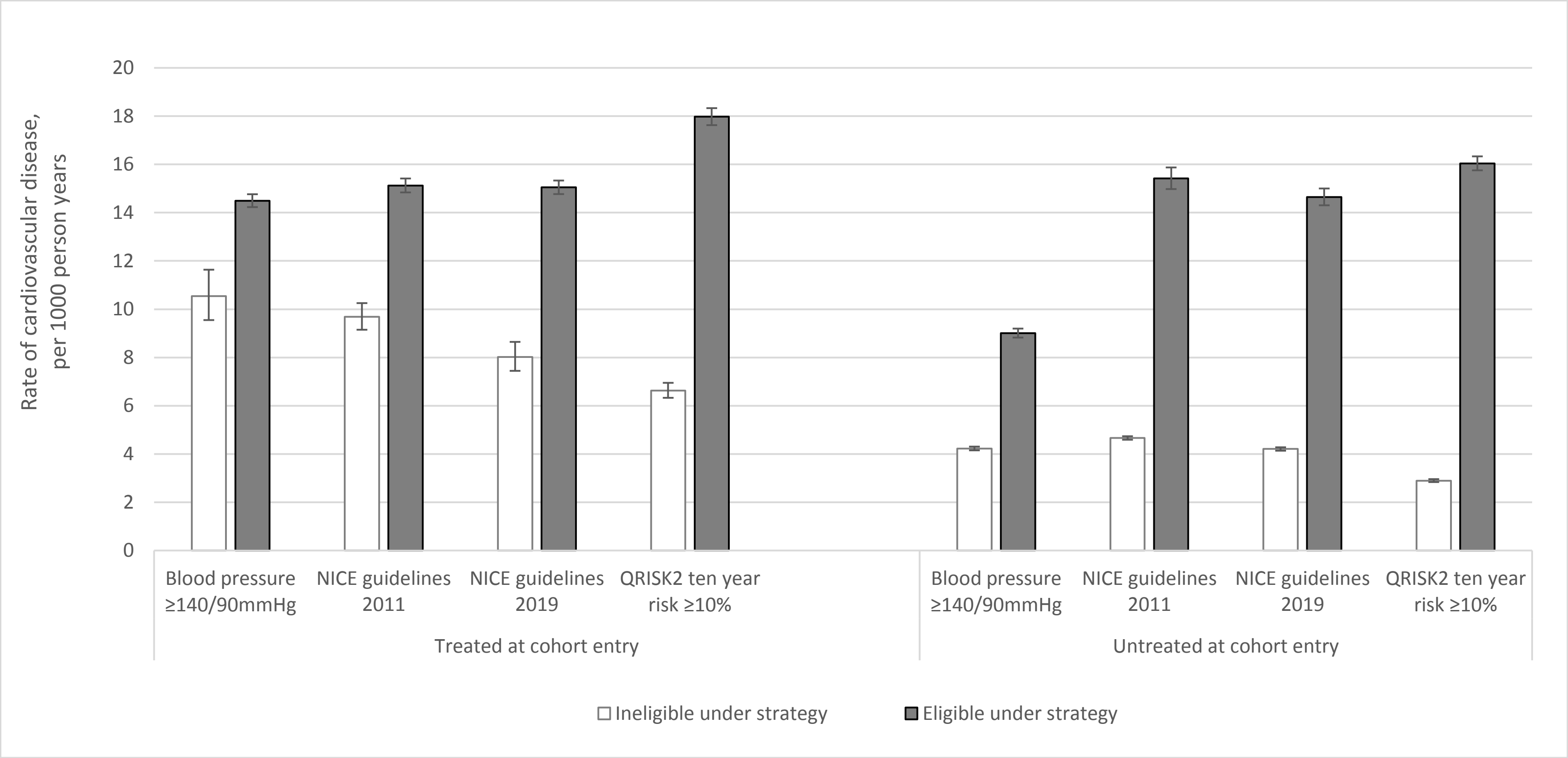

Appendix Figure 6. Rate of cardiovascular disease per 1000 person years (with 95% confidence intervals) among patients eligible and ineligible for blood pressure lowering treatment under each treatment strategy (blood pressure ≥140/90mmHg, NICE 2011 guidelines, NICE 2019 guidelines, QRISK2 ten year risk ≥10%), stratified by treatment with blood pressure lowering at cohort entry.

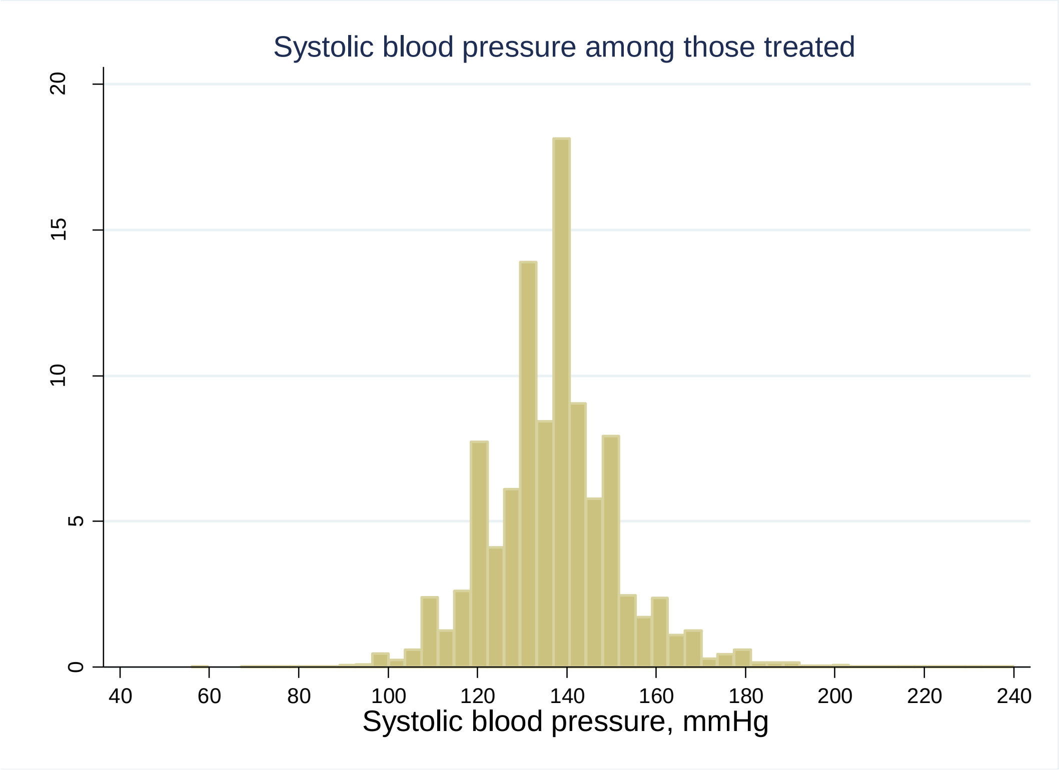

Appendix Figure 7. Distribution of systolic blood pressures (recorded closest to cohort entry date 1st January 2011) among patients who were receiving blood pressure lowering treatment at cohort entry.

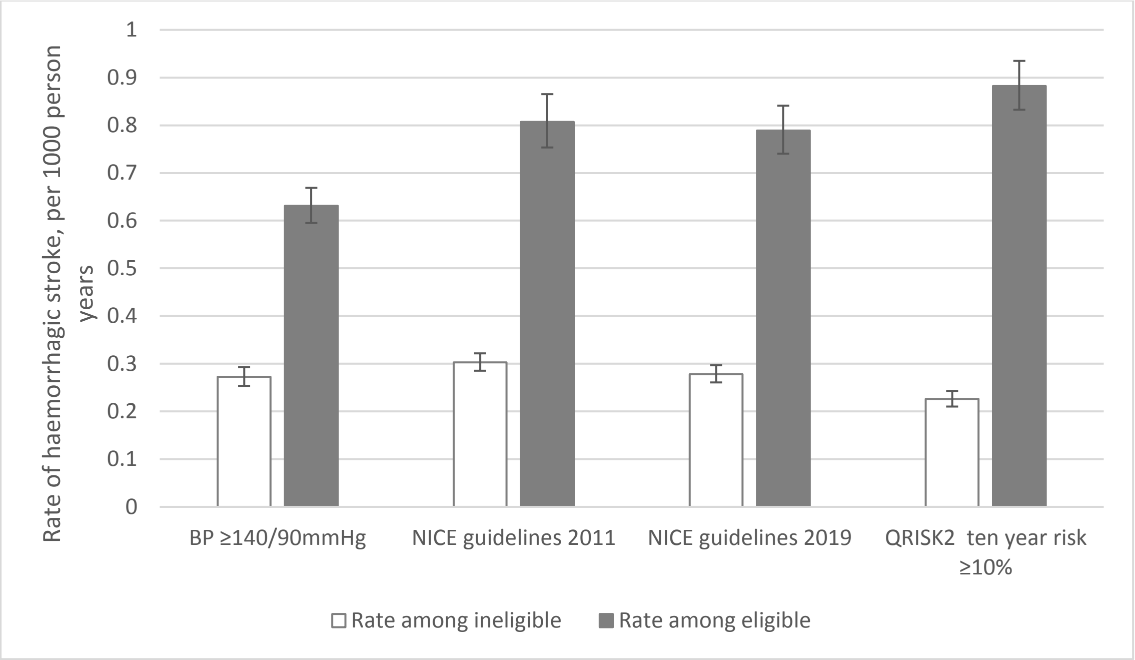

Appendix Figure 8. Rate of haemorrhagic stroke per 1000 person years among patients who were classified as eligible and ineligible for blood pressure lowering treatment under each treatment strategy (blood pressure ≥140/90mmHg, NICE 2011 guidelines, NICE 2019 guidelines, QRISK2 ten year risk ≥10%) with 95% confidence intervals. BP: blood pressure

Code lists for use with CPRD, HES and ONS data

Medical code list for cardiovascular disease in CPRD

- any_chd: any code indicating coronary heart disease; including stable angina, percutaneous coronary intervention, coronary artery bypass graft, myocardial infarction, acute coronary syndrome, unstable angina, heart failure, sudden cardiac death, coronary thrombolysis, coronary heart disease not otherwise specified.
- any_cvd: any code indicating cerebrovascular disease; including stroke and non-stroke cerebrovascular disease.
- any_pad: any code indicating peripheral arterial disease; including peripheral arterial disease, abdominal aortic aneurysm, procedures to repair these.
- any_acs: any code indicating unstable angina, acute coronary syndrome, myocardial infarction, coronary thrombolysis.
- any_stroke: any code indicating ischaemic, haemorrhagic stroke or stroke not otherwise specified.

Medcode	Read code	Read terms	any_chd	any_cvd	any_pad	any_acs	any_stroke
6336	14A5.00	H/O: angina pectoris	1				
57062	14AJ.00	H/O: Angina in last year	1				
103655	187..00	Frequency of angina	1				
107967	661M000	Angina self-management plan agreed	1				
109391	661N000	Angina self-management plan review	1				
13185	662K.00	Angina control	1				
19542	662K000	Angina control - good	1				
15373	662K100	Angina control - poor	1				
14782	662K200	Angina control - improving	1				
108504	662K400	Angina self management plan commenced	1				
108506	662K500	Angina self management plan completed	1				
15349	662Kz00	Angina control NOS	1				
45960	8B27.00	Antianginal therapy	1				
108056	8IEY.00	Referral to Angina Plan self-management programme declined	1				
107574	8T04.00	Referral to Angina Plan self-management programme	1				
1430	G33..00	Angina pectoris	1				
20095	G330.00	Angina decubitus	1				
18125	G330000	Nocturnal angina	1				
29902	G330z00	Angina decubitus NOS	1				
25842	G33z.00	Angina pectoris NOS	1				
54535	G33z100	Stenocardia	1				
7696	G33z200	Syncope anginosa	1				

1414	G33z300	Angina on effort	1				
9555	G33z500	Post infarct angina	1				
26863	G33z600	New onset angina	1				
12804	G33z700	Stable angina	1				
28554	G33zz00	Angina pectoris NOS	1				
24540	G34y000	Chronic coronary insufficiency	1				
39546	Gyu3000	[X]Other forms of angina pectoris	1				
32450	G33z400	Ischaemic chest pain	1				
737	792..11	Coronary artery bypass graft operations	1				
3159	792Dy00	Other specified other bypass of coronary artery	1				
5030	ZV45K00	[V]Presence of coronary artery bypass graft	1				
5674	ZV45K11	[V]Presence of coronary artery bypass graft - CABG	1				
5744	7927500	Open angioplasty of coronary artery	1				
7134	7921.11	Other autograft bypass of coronary artery	1				
7137	7920y00	Saphenous vein graft replacement of coronary artery OS	1				
7442	7920200	Saphenous vein graft replacement of three coronary arteries	1				
7609	7921z00	Other autograft replacement of coronary artery NOS	1				
7634	7920100	Saphenous vein graft replacement of two coronary arteries	1				
8312	7920.11	Saphenous vein graft bypass of coronary artery	1				
8679	7920000	Saphenous vein graft replacement of one coronary artery	1				
9414	7921	Other autograft replacement of coronary artery	1				
10209	7921200	Autograft replacement of three coronary arteries NEC	1				
11610	7920300	Saphenous vein graft replacement of four+ coronary arteries	1				
12734	SP07600	Coronary artery bypass graft occlusion	1				
18249	7920	Saphenous vein graft replacement of coronary artery	1				
18913	ZV45700	[V]Presence of aortocoronary bypass graft	1				
19193	7923z00	Prosthetic replacement of coronary artery NOS	1				
19402	7923	Prosthetic replacement of coronary artery	1				
19413	7921100	Autograft replacement of two coronary arteries NEC	1				
22647	7925311	LIMA single anastomosis	1				
28837	7925.11	Creation of bypass from mammary artery to coronary artery	1				
31519	7925100	Double implant of mammary arteries into coronary arteries	1				
31540	7924200	Revision of bypass for three coronary arteries	1				

31556	7922	Allograft replacement of coronary artery	1					
32651	7922.11	Allograft bypass of coronary artery	1					
33461	7924	Revision of bypass for coronary artery	1					
33471	792Dz00	Other bypass of coronary artery NOS	1					
33718	7925000	Double anastomosis of mammary arteries to coronary arteries	1					
34963	792D.00	Other bypass of coronary artery	1					
36011	7923.11	Prosthetic bypass of coronary artery	1					
37682	7925	Connection of mammary artery to coronary artery	1					
37719	7925y00	Connection of mammary artery to coronary artery OS	1					
42708	7921300	Autograft replacement of four of more coronary arteries NEC	1					
44561	7921000	Autograft replacement of one coronary artery NEC	1					
45370	7922300	Allograft replacement of four or more coronary arteries	1					
45886	7922200	Allograft replacement of three coronary arteries	1					
48767	7922z00	Allograft replacement of coronary artery NOS	1					
51507	7925300	Single anastomosis of mammary artery to coronary artery NEC	1					
51515	7920z00	Saphenous vein graft replacement coronary artery NOS	1					
52938	7924000	Revision of bypass for one coronary artery	1					
55092	792C000	Replacement of coronary arteries using multiple methods	1					
55598	792C.00	Other replacement of coronary artery	1					
56990	7925z00	Connection of mammary artery to coronary artery NOS	1					
57241	7922100	Allograft replacement of two coronary arteries	1					
57634	7924z00	Revision of bypass for coronary artery NOS	1					
59423	7922y00	Other specified allograft replacement of coronary artery	1					
60753	7926300	Single implantation thoracic artery into coronary artery NEC	1					
61310	7921y00	Other autograft replacement of coronary artery OS	1					
62608	7926000	Double anastom thoracic arteries to coronary arteries NEC	1					
66236	7923200	Prosthetic replacement of three coronary arteries	1					
66664	7923100	Prosthetic replacement of two coronary arteries	1					
67554	7924100	Revision of bypass for two coronary arteries	1					
67591	7926200	Single anastomosis of thoracic artery to coronary artery NEC	1					
67761	7923300	Prosthetic replacement of four or more coronary arteries	1					
68123	7925312	RIMA single anastomosis	1					
68139	7925400	Single implantation of mammary artery into coronary artery	1					

69776	SP00300	Mechanical complication of coronary bypass	1				
70111	7922000	Allograft replacement of one coronary artery	1				
70755	792Cz00	Replacement of coronary artery NOS	1				
72780	7926z00	Connection of other thoracic artery to coronary artery NOS	1				
92419	7923000	Prosthetic replacement of one coronary artery	1				
93828	792Cy00	Other specified replacement of coronary artery	1				
96804	7926	Connection of other thoracic artery to coronary artery	1				
97953	7924y00	Other specified revision of bypass for coronary artery	1				
101569	7924300	Revision of bypass for four or more coronary arteries	1				
18643	ZV45800	[V]Presence of coronary angioplasty implant and graft	1				
6980	ZV45L00	[V]Status following coronary angioplasty NOS	1				
2901	7928	Transluminal balloon angioplasty of coronary artery	1				
5703	7928.11	Percutaneous balloon coronary angioplasty	1				
18670	7928000	Percut transluminal balloon angioplasty one coronary artery	1				
33735	7928100	Percut translum balloon angioplasty mult coronary arteries	1				
42462	7928200	Percut translum balloon angioplasty bypass graft coronary a	1				
86071	7928300	Percut translum cutting balloon angioplasty coronary artery	1				
41547	7928y00	Transluminal balloon angioplasty of coronary artery OS	1				
732	7928z00	Transluminal balloon angioplasty of coronary artery NOS	1				
22828	7929000	Percutaneous transluminal laser coronary angioplasty	1				
19046	7929300	Rotary blade coronary angioplasty	1				
8942	7929400	Insertion of coronary artery stent	1				
42304	7929500	Insertion of drug-eluting coronary artery stent	1				
93618	7929600	Percutaneous transluminal atherectomy of coronary artery	1				
22020	792B000	Endarterectomy of coronary artery NEC	1				
105184	792E.00	Percutaneous coronary intervention	1				
107406	7.92E+02	Emergency percutaneous coronary intervention	1				
43939	793G.00	Perc translumin balloon angioplasty stenting coronary artery	1				
60067	793G000	Perc translum ball angio insert 1-2 drug elut stents cor art	1				
87849	793G100	Perc tran ball angio ins 3 or more drug elut stents cor art	1				
85947	793G200	Perc translum balloon angioplasty insert 1-2 stents cor art	1				
92927	793G300	Percutaneous cor balloon angiop 3 more stents cor art NEC	1				
96537	793Gy00	OS perc translumina balloon angioplast stenting coronary art	1				

61208	793Gz00	Perc translum balloon angioplasty stenting coronary art NOS	1				
733	7A54000	Percutaneous transluminal angioplasty of artery NEC	1				
38813	7A54500	Rotary blade angioplasty	1				
70185	7A54800	Percutaneous transluminal atherectomy	1				
86773	7A56400	Percutaneous transluminal balloon angioplasty of artery	1				
20903	7A6G100	Peroperative angioplasty	1				
64923	7A6H300	Prosthetic graft patch angioplasty	1				
66921	7A6H400	Percutaneous transluminal angioplasty of vascular graft	1				
8246	322..00	ECG: myocardial ischaemia	1				
26973	3222	ECG:shows myocardial ischaemia	1				
35287	322Z.00	ECG: myocardial ischaemia NOS	1				
42104	32E4.00	ECG: S-T depression	1				
26965	32F2.00	ECG: T wave abnormal	1				
46230	32F3.00	ECG: T wave flattened	1				
26967	32F4.00	ECG: T wave inverted	1				
26966	32E3.00	ECG: S-T elevation	1			1	
7783	323..00	ECG: myocardial infarction	1			1	
39904	3232	ECG: old myocardial infarction	1			1	
26975	3233	ECG: antero-septal infarct.	1			1	
26972	3234	ECG:posterior/inferior infarct	1			1	
55401	3235	ECG: subendocardial infarct	1			1	
52705	3236	ECG: lateral infarction	1			1	
59032	323Z.00	ECG: myocardial infarct NOS	1			1	
46227	32B2.00	ECG: Q wave abnormal	1			1	
62270	32B3.00	ECG: Q wave pathological	1			1	
1628	3213100	Exercise ECG abnormal	1				
19827	3213111	Positive exercise ECG test	1				
5233	33B9500	Exercise tolerance test abnormal	1				
58135	5C11.00	Radionuclide heart study abnormal	1				
1021	5543	Coronary arteriograph.abnormal	1				
35382	5533	Angiocardiography abnormal	1				
3999	G340000	Single coronary vessel disease	1				
5254	G340100	Double coronary vessel disease	1				

1655	G340.11	Triple vessel disease of the heart	1				
16993	14AE.00	H/O: aortic aneurysm			1		
1735	G71..00	Aortic aneurysm			1		
17767	G713.00	Abdominal aortic aneurysm which has ruptured			1		
13572	G713.11	Ruptured abdominal aortic aneurysm			1		
63920	G713000	Ruptured suprarenal aortic aneurysm			1		
1867	G714.00	Abdominal aortic aneurysm without mention of rupture			1		
17345	G714.11	AAA - Abdominal aortic aneurysm without mention of rupture			1		
45521	G714000	Juxtarenal aortic aneurysm			1		
101379	G714200	Infrarenal abdominal aortic aneurysm			1		
101195	G714300	Aneurysm of suprarenal aorta			1		
15304	G715.00	Ruptured aortic aneurysm NOS			1		
11430	G715000	Thoracoabdominal aortic aneurysm, ruptured			1		
16034	G716.00	Aortic aneurysm without mention of rupture NOS			1		
40787	G716000	Thoracoabdominal aortic aneurysm, without mention of rupture			1		
9759	G718.00	Leaking abdominal aortic aneurysm			1		
6872	G71z.00	Aortic aneurysm NOS			1		
102725	Gyu7100	[X]Aortic aneurysm of unspecified site, ruptured			1		
102719	Gyu7200	[X]Aortic aneurysm of unspecified site, nonruptured			1		
105621	Gyu7800	[X]Aneurysm of aorta in diseases classified elsewhere			1		
17328	7A1..00	Aorta operations			1		
66914	7A10.00	Extraanatomic bypass of aorta			1		
102684	7A10400	Bypass aorta anastomosis axillary artery bi femoral arteries			1		
67839	7A10y00	Other specified extraanatomic bypass of aorta			1		
100712	7A10z00	Extraanatomic bypass of aorta NOS			1		
98082	7A15300	Emerg bypass infrarenal aorta by anastom aorta to aorta NEC			1		
101835	7A15y00	Other specified other emergency bypass of segment of aorta			1		
104815	7A15z00	Other emergency bypass of segment of aorta NOS			1		
37639	7A16.00	Other bypass of segment of aorta			1		
61978	7A16300	Bypass of infrarenal aorta by anastomosis aorta to aorta NEC			1		
5597	7A16400	Bypass of abdominal aorta by anastomosis aorta to aorta NEC			1		
59518	7A16y00	Other specified other bypass of segment of aorta			1		
42751	7A16z00	Other bypass of segment of aorta NOS			1		

98211	7A17.00	Attention to prosthesis of aorta			1		
66316	7A17100	Revision of prosthesis of bifurcation of aorta			1		
65813	7A17200	Revision of prosthesis of abdominal aorta NEC			1		
95721	7A17300	Removal of prosthesis from aorta			1		
107284	7A17y00	Other specified attention to prosthesis of aorta			1		
97596	7A17z00	Attention to prosthesis of aorta NOS			1		
24371	7A18.00	Plastic repair of aorta			1		
39962	7A18000	Plastic repair of aorta and end to end anastomosis of aorta			1		
49878	7A18100	Plastic repair of aorta using subclavian flap			1		
55319	7A18200	Plastic repair of aorta using patch graft			1		
105136	7A18400	Revision of plastic repair of aorta			1		
52497	7A18y00	Other specified plastic repair of aorta			1		
20078	7A18z00	Plastic repair of aorta NOS			1		
53163	7A19.00	Other open operations on aorta			1		
43179	7A19000	Endarterectomy of aorta and patch repair of aorta			1		
36008	7A19100	Endarterectomy of aorta NEC			1		
86053	7A19y00	Other specified other open operation on aorta			1		
36928	7A19z00	Other open operation on aorta NOS			1		
57141	7A1A.00	Transluminal operations on aorta			1		
57539	7A1A.11	Percutaneous transluminal operations on aorta			1		
30296	7A1A000	Percutaneous transluminal balloon angioplasty of aorta			1		
30466	7A1A100	Percutaneous transluminal angioplasty of aorta NEC			1		
50122	7A1A200	Percutaneous transluminal embolectomy of aortic bifurcation			1		
89198	7A1A500	Percutaneous transluminal balloon angioplasty stenting aorta			1		
86810	7A1A600	Percutan transluminal aortic stent graft fenestration NEC			1		
87370	7A1A700	Percutaneous transluminal aortic stent graft branches NEC			1		
96912	7A1A800	Transluminal aortic stent graft with fenestration NEC			1		
89731	7A1AA00	Percutaneous transluminal insertion of stent into aorta			1		
53891	7A1Ay00	Other specified transluminal operation on aorta			1		
48768	7A1Az00	Transluminal operation on aorta NOS			1		
99787	7A1BB00	Endovascular ins stent for aortic dissection in any position			1		
35023	7A1y.00	Other specified operations on aorta			1		
25735	7A1z.00	Aorta operations NOS			1		

42142	7A3..00	Branches of abdominal aorta operations			1		
101017	7A3y.00	Other specified operations on branches of abdominal aorta			1		
38546	7A1..11	Dacron graft operations on aorta			1		
52358	7A11.00	Replacement of aneurysmal bifurcation of aorta			1		
96654	7A11000	Emerg repl aneurysm bifurc aorta by anast aorta to fem art			1		
56495	7A11100	Replace aneurysm bifurc aorta by anast aorta to femoral art			1		
69922	7A11200	Emerg repl aneurysm bifurc aorta by anast aorta to iliac a			1		
92925	7A11211	Y graft of abdominal Aortic aneurysm (emergency)			1		
56510	7A11300	Replace aneurysm bifurc aorta by anast aorta to iliac artery			1		
51166	7A11311	Y graft abdominal Aortic aneurysm			1		
62301	7A11y00	Replacement of aneurysmal bifurcation of aorta OS			1		
66761	7A11z00	Replacement of aneurysmal bifurcation of aorta NOS			1		
31613	7A13.00	Emergency replacement of aneurysmal segment of aorta			1		
17220	7A13.11	Emergency repair of aortic aneurysm			1		
99722	7A13000	Emerg replace aneurysm asc aorta by anastom aorta to aorta			1		
66232	7A13300	Emerg replace aneurysm infrarenal aorta by anast aorta/aorta			1		
54192	7A13400	Emerg replace aneurysm abdom aorta by anast aorta/aorta NEC			1		
63408	7A13411	Tube graft abdominal Aortic aneurysm (emergency)			1		
45477	7A13y00	Emergency replacement of aneurysmal segment of aorta OS			1		
45474	7A13z00	Emergency replacement of aneurysmal segment of aorta NOS			1		
43108	7A14.00	Other replacement of aneurysmal segment of aorta			1		
1736	7A14.11	Aortic aneurysm repair			1		
54379	7A14200	Replace aneurys suprarenal aorta by anast aorta to aorta NEC			1		
44553	7A14300	Replace aneurys infrarenal aorta by anast aorta to aorta NEC			1		
19996	7A14400	Replace aneurysm abdominal aorta by anast aorta to aorta NEC			1		
26232	7A14411	Tube graft of Abdominal aortic aneurysm			1		
55445	7A14y00	Other replacement of aneurysmal segment of aorta OS			1		
36651	7A14z00	Other replacement of aneurysmal segment of aorta NOS			1		
31822	7A15.00	Other emergency bypass of segment of aorta			1		
102031	7A15400	Emerg bypass abdominal aorta by anastom aorta to aorta NEC			1		
33430	7A19400	Operation on aneurysm of aorta NEC			1		
93959	7A1B.00	Transluminal operations on aneurysmal segment of aorta			1		
70446	7A1B000	Endovascular stenting infrarenal abdominal aortic aneurysm			1		

97030	7A1B100	Endovascular stenting of suprarenal aortic aneurysm			1		
90549	7A1B400	Endovascular stenting of aortic bifurcation NEC			1		
95976	7A1B500	Endovascular stenting of aorto-uniiliac aneurysm			1		
100195	7A1B600	Endovascular stenting for aortic aneurysm of bifurcation NEC			1		
97109	7A1B700	Endovascular stenting for aorto-uniiliac aneurysm			1		
97217	7A1B800	Endovascul insert stent infrarenal abdominal aortic aneurysm			1		
106780	7A1B900	Endovascular insertion stent for suprarenal aortic aneurysm			1		
99859	7A1BC00	Endovas insert stent for aortic aneurysm of bifurcation NEC			1		
98175	7A1BD00	Endovascular insertion of stent for aorto-uniiliac aneurysm			1		
90861	7A1Bz00	Transluminal operations on aneurysmal segment of aorta NOS			1		
94331	7A1C.00	Translum insert stent graft for aneurysmal segment of aorta			1		
83577	7A1C000	Endovas ins stent graft for infrarenal abdom aortic aneurysm			1		
94682	7A1C100	Endovas insert of stent graft for suprarenal aortic aneurysm			1		
94069	7A1C400	Endovas insertion of stent graft for aortic bifurcation NEC			1		
103427	7A1C500	Endovas insertion of stent graft for aorto-uniiliac aneurysm			1		
98565	7A1Cy00	OS translum ins stent graft for aneurysmal segment of aorta			1		
93627	7A1Cz00	Translum ins stent graft for aneurysmal segment of aorta NOS			1		
105216	14AW.00	H/O acute coronary syndrome	1			1	
11983	G311500	Acute coronary syndrome	1			1	
5221	44H3.00	Cardiac enzymes abnormal	1			1	
60664	44H3000	Cardiac enzymes abnormal - first set	1			1	
28914	662o.00	Haemorrhagic stroke monitoring		1			1
48149	G681.00	Sequelae of intracerebral haemorrhage		1			1
43451	G682.00	Sequelae of other nontraumatic intracranial haemorrhage		1			1
5051	G61..00	Intracerebral haemorrhage		1			1
6960	G61..11	CVA - cerebrovascular accid due to intracerebral haemorrhage		1			1
18604	G61..12	Stroke due to intracerebral haemorrhage		1			1
31595	G610.00	Cortical haemorrhage		1			1
40338	G611.00	Internal capsule haemorrhage		1			1
46316	G612.00	Basal nucleus haemorrhage		1			1
13564	G613.00	Cerebellar haemorrhage		1			1
7912	G614.00	Pontine haemorrhage		1			1
30045	G616.00	External capsule haemorrhage		1			1

30202	G617.00	Intracerebral haemorrhage, intraventricular		1			1
57315	G618.00	Intracerebral haemorrhage, multiple localized		1			1
107440	G619.00	Lobar cerebral haemorrhage		1			1
31060	G61X.00	Intracerebral haemorrhage in hemisphere, unspecified		1			1
28314	G61X000	Left sided intracerebral haemorrhage, unspecified		1			1
19201	G61X100	Right sided intracerebral haemorrhage, unspecified		1			1
3535	G61z.00	Intracerebral haemorrhage NOS		1			1
53810	Gyu6200	[X]Other intracerebral haemorrhage		1			1
96630	Gyu6F00	[X]Intracerebral haemorrhage in hemisphere, unspecified		1			1
56007	G601.00	Subarachnoid haemorrhage from carotid siphon and bifurcation		1			1
19412	G602.00	Subarachnoid haemorrhage from middle cerebral artery		1			1
17326	G60X.00	Subarachnoid haemorrh from intracranial artery, unspecif		1			1
108668	Gyu6000	[X]Subarachnoid haemorrhage from other intracranial arteries		1			1
108630	Gyu6E00	[X]Subarachnoid haemorrh from intracranial artery, unspecif		1			1
4917	7017000	Evacuation of subdural haematoma		1			1
4273	G621.00	Subdural haemorrhage - nontraumatic		1			1
17734	G622.00	Subdural haematoma - nontraumatic		1			1
18912	G623.00	Subdural haemorrhage NOS		1			1
6569	S62..13	Subdural haemorrhage following injury		1			1
2883	S622.00	Closed traumatic subdural haemorrhage		1			1
94351	S623.00	Open traumatic subdural haemorrhage		1			1
8181	S628.00	Traumatic subdural haemorrhage		1			1
7862	S629.00	Traumatic subdural haematoma		1			1
53980	S629000	Traumatic subdural haematoma without open intracranial wound		1			1
96677	S629100	Traumatic subdural haematoma with open intracranial wound		1			1
4107	7032000	Evacuation of extradural haematoma		1			1
36178	G620.00	Extradural haemorrhage - nontraumatic		1			1
27661	S62..11	Extradural haemorrhage following injury		1			1
45421	S624.00	Closed traumatic extradural haemorrhage		1			1
43418	S624.11	Epidural haematoma following injury		1			1
73471	S625.00	Open traumatic extradural haemorrhage		1			1
51504	S626.00	Epidural haemorrhage		1			1
18411	S62A.00	Traumatic extradural haematoma		1			1

109369	S62A100	Traumatic extradural haematoma with open intracranial wound		1			1
31805	G62..00	Other and unspecified intracranial haemorrhage		1			1
20284	G62z.00	Intracranial haemorrhage NOS		1			1
31941	A94y600	Rupture of syphilitic cerebral aneurysm		1			1
5682	S62..00	Cerebral haemorrhage following injury		1			1
28077	S62..14	Traumatic cerebral haemorrhage		1			1
46545	S62z.00	Cerebral haemorrhage following injury NOS		1			1
52968	S63..00	Other cerebral haemorrhage following injury		1			1
42283	S63z.00	Other cerebral haemorrhage following injury NOS		1			1
35916	7A20300	Endarterectomy and patch repair of carotid artery		1			1
12733	7A20311	Carotid endarterectomy and patch		1			1
2654	7A20400	Endarterectomy of carotid artery NEC		1			1
93134	7A20500	High-flow inter extrac intrac byp ext carot art mid cer art		1			1
89365	7A20600	Byp carot art anastom superfic tempor artery middle cere art		1			1
91775	7A20700	Intracranial bypass from carotid artery NEC		1			1
29973	7A22000	Percutaneous transluminal angioplasty of carotid artery		1			1
47580	7A22300	Percutaneous transluminal insertion stent carotid artery		1			1
93770	7A25600	Percutaneous transluminal insertion of stent cerebral artery		1			1
68906	7A26C00	Endarterectomy of vertebral artery NEC		1			1
55074	7A28200	Percutaneous transluminal angioplasty of vertebral artery		1			1
68905	7A28500	Percutaneous transluminal embolectomy of vertebral artery		1			1
45476	14AL.00	H/O: Treatment for ischaemic heart disease	1				
11798	14A..12	H/O: myocardial problem	1				
32526	14AA.00	H/O: heart disease NOS	1				
3468	662..00	Cardiac disease monitoring	1				
1537	662..11	Heart disease monitoring	1				
46664	6625	Cardiac drug side effects	1				
26044	6626	Cardiac treatment changed	1				
34488	662J.00	Cardiac drug monitoring	1				
42659	662JZ00	Cardiac drug monitoring NOS	1				
13187	662N.00	CHD monitoring	1				
19298	662Y.00	Cardiac event recording	1				
17681	662Z.00	Cardiac disease monitoring NOS	1				

19185	66f..00	Cardiovascular disease monitoring	1				
48981	66f1.00	Cardiovascular disease interim monitoring	1				
19164	7927100	Repair of aneurysm of coronary artery	1				
72925	8A51.00	Cardiac emergency monitoring	1				
100496	8CEJ.00	Coronary heart disease leaflet given	1				
103932	8CMP.00	Coronary heart disease care plan	1				
8516	8F9..00	Cardiac rehabilitation	1				
37990	8F90.00	Cardiac rehabilitation - phase 1	1				
37991	8F91.00	Cardiac rehabilitation - phase 2	1				
41032	8F92.00	Cardiac rehabilitation - phase 3	1				
46565	8F93.00	Cardiac rehabilitation - phase 4	1				
104675	8F97.00	Cardiac rehabilitation programme completed	1				
110535	8F98.00	Cardiac rehabilitation programme offered	1				
38379	8H11.00	Admit to cardiac ITU	1				
30027	8H2Q.00	Admit cardiology emergency	1				
106415	8H44000	Referral to cardiology multidisciplinary team	1				
10127	8H7v.00	Referral to cardiac rehabilitation nurse	1				
104176	8HRG.00	Referral for cardiac event recording	1				
102447	8Hkk.00	Referral to cardiac rehabilitation programme	1				
102943	8Hkl.00	Referral to cardiac rehabilitation service by secondary care	1				
103632	8Hkt.00	Referral to community cardiology service	1				
19744	8I37.00	Coronary heart disease monitoring refused	1				
19250	8I3a.00	Cardiac rehabilitation declined	1				
102914	8IE3.00	Referral to cardiac rehabilitation programme declined	1				
18150	9Ob..00	Coronary heart disease monitoring administration	1				
35373	9Ob0.00	Attends coronary heart disease monitoring	1				
35277	9Ob1.00	Refuses coronary heart disease monitoring	1				
47798	9Ob2.00	Coronary heart disease monitoring default	1				
25814	9Ob3.00	Coronary heart disease monitoring 1st letter	1				
34207	9Ob4.00	Coronary heart disease monitoring 2nd letter	1				
34329	9Ob5.00	Coronary heart disease monitoring 3rd letter	1				
37908	9Ob6.00	Coronary heart disease monitoring verbal invitation	1				
54007	9Ob7.00	Coronary heart disease monitoring deleted	1				

39500	9Ob8.00	Coronary heart disease monitoring check done	1				
70160	9Ob9.00	Coronary heart disease monitoring telephone invite	1				
10963	9h0..00	Exception reporting: CHD quality indicators	1				
11038	9h01.00	Excepted from CHD quality indicators: Patient unsuitable	1				
10910	9h02.00	Excepted from CHD quality indicators: Informed dissent	1				
13250	G....12	Cardiac diseases	1				
10109	G....13	Heart diseases	1				
6331	G341.00	Aneurysm of heart	1				
27484	G341.11	Cardiac aneurysm	1				
2155	G341000	Ventricular cardiac aneurysm	1				
67087	G341100	Other cardiac wall aneurysm	1				
105250	G341111	Mural cardiac aneurysm	1				
59193	G341200	Aneurysm of coronary vessels	1				
41677	G341z00	Aneurysm of heart NOS	1				
30171	G5...00	Other forms of heart disease	1				
36193	G5y..00	Other specified heart disease	1				
59687	G5yy.00	Other ill-defined heart disease	1				
41179	G5yyz00	Other ill-defined heart disease NOS	1				
1811	G5yz.00	Other heart disease NOS	1				
1490	G5z..00	Heart disease NOS	1				
68979	Gyu5.00	[X]Other forms of heart disease	1				
18218	Z677.00	Cardiac rehabilitation class	1				
10662	ZL18300	Under care of cardiologist	1				
32666	ZL1G200	Under care of cardiothoracic surgeon	1				
42669	ZL1G400	Under care of cardiac surgeon	1				
19067	ZL22200	Under care of cardiac rehabilitation nurse	1				
23098	ZV57900	[V]Cardiac rehabilitation	1				
18135	6A2..00	Coronary heart disease annual review	1				
10260	6A4..00	Coronary heart disease review	1				
11648	8B3k.00	Coronary heart disease medication review	1				
95550	8H2V.00	Admit ischaemic heart disease emergency	1				
240	G3...00	Ischaemic heart disease	1				
24783	G3...11	Arteriosclerotic heart disease	1				

20416	G3...12	Atherosclerotic heart disease	1				
1792	G3...13	IHD - Ischaemic heart disease	1				
27951	G31..00	Other acute and subacute ischaemic heart disease	1				
61072	G311000	Myocardial infarction aborted	1				
55137	G311011	MI - myocardial infarction aborted	1				
9413	G31y.00	Other acute and subacute ischaemic heart disease	1				
39693	G31y200	Subendocardial ischaemia	1				
21844	G31y300	Transient myocardial ischaemia	1				
27977	G31yz00	Other acute and subacute ischaemic heart disease NOS	1				
28138	G34..00	Other chronic ischaemic heart disease	1				
5413	G340.00	Coronary atherosclerosis	1				
1344	G340.12	Coronary artery disease	1				
7320	G343.00	Ischaemic cardiomyopathy	1				
29421	G344.00	Silent myocardial ischaemia	1				
34633	G34y.00	Other specified chronic ischaemic heart disease	1				
23078	G34y100	Chronic myocardial ischaemia	1				
35713	G34yz00	Other specified chronic ischaemic heart disease NOS	1				
15754	G34z.00	Other chronic ischaemic heart disease NOS	1				
18889	G34z000	Asymptomatic coronary heart disease	1				
22383	G3y..00	Other specified ischaemic heart disease	1				
1676	G3z..00	Ischaemic heart disease NOS	1				
52517	Gyu3.00	[X]Ischaemic heart diseases	1				
68401	Gyu3200	[X]Other forms of acute ischaemic heart disease	1				
47637	Gyu3300	[X]Other forms of chronic ischaemic heart disease	1				
23268	5853	U-S heart scan	1				
1271	5853.11	Echocardiogram	1				
1432	5853000	Echocardiogram normal	1				
5245	5853100	Echocardiogram abnormal	1				
27851	5853z00	U-S heart scan NOS	1				
18508	585R.00	Echocardiogram normal	1				
11284	585f.00	Echocardiogram shows left ventricular systolic dysfunction	1				
11351	585g.00	Echocardiogram shows left ventricular diastolic dysfunction	1				
12314	585k.00	Echocardiogram shows normal left ventricular function	1				

30917	5C20.00	Echocardiogram equivocal	1					
3919	7935200	Transoesophageal echocardiography	1					
89353	7935500	Transluminal intracardiac echocardiography	1					
26449	7P0H.00	Diagnostic echocardiography	1					
26445	7P0H000	Transthoracic echocardiography	1					
85952	7P0H100	Transoesophageal echocardiography	1					
94626	7P0H300	Epicardial echocardiography	1					
105341	7P0H600	Contrast echocardiography	1					
99841	7P0Hy00	Other specified diagnostic echocardiography	1					
90493	7P0Hz00	Diagnostic echocardiography NOS	1					
105649	8A54400	Monitoring of cardiac output using echocardiography	1					
106377	9EV7.00	Echocardiography report received	1					
101184	9Ee0800	Adult echocardiography procedure report	1					
10317	R132000	[D]Echocardiogram abnormal	1					
40366	R132200	[D]Ultrasound cardiogram abnormal	1					
15058	14A6.00	H/O: heart failure	1					
46912	14AM.00	H/O: Heart failure in last year	1					
9913	1O1..00	Heart failure confirmed	1					
100784	2126400	Heart failure resolved	1					
106198	661M500	Heart failure self-management plan agreed	1					
30779	662W.00	Heart failure annual review	1					
83502	662p.00	Heart failure 6 month review	1					
24503	8B29.00	Cardiac failure therapy	1					
103732	8CMK.00	Has heart failure management plan	1					
32898	8H2S.00	Admit heart failure emergency	1					
19380	9Or0.00	Heart failure review completed	1					
22262	G1yz100	Rheumatic left ventricular failure	1					
50157	G210.00	Malignant hypertensive heart disease	1					
95334	G210000	Malignant hypertensive heart disease without CCF	1					
72668	G210100	Malignant hypertensive heart disease with CCF	1					
103046	G210z00	Malignant hypertensive heart disease NOS	1					
52127	G211100	Benign hypertensive heart disease with CCF	1					
62718	G21z100	Hypertensive heart disease NOS with CCF	1					

67232	G230.00	Malignant hypertensive heart and renal disease	1					
21837	G232.00	Hypertensive heart&renal dis wth (congestive) heart failure	1					
57987	G234.00	Hyperten heart&renal dis+both(congestv)heart and renal fail	1					
8464	G400.00	Acute cor pulmonale	1					
5695	G41z.11	Chronic cor pulmonale	1					
5141	G554000	Congestive cardiomyopathy	1					
68766	G554011	Congestive obstructive cardiomyopathy	1					
2062	G58..00	Heart failure	1					
1223	G58..11	Cardiac failure	1					
398	G580.00	Congestive heart failure	1					
2906	G580.11	Congestive cardiac failure	1					
10079	G580.12	Right heart failure	1					
10154	G580.13	Right ventricular failure	1					
9524	G580.14	Biventricular failure	1					
23707	G580000	Acute congestive heart failure	1					
32671	G580100	Chronic congestive heart failure	1					
27884	G580200	Decompensated cardiac failure	1					
11424	G580300	Compensated cardiac failure	1					
94870	G580400	Congestive heart failure due to valvular disease	1					
884	G581.00	Left ventricular failure	1					
23481	G581.11	Asthma - cardiac	1					
5942	G581.13	Impaired left ventricular function	1					
5255	G581000	Acute left ventricular failure	1					
27964	G582.00	Acute heart failure	1					
101138	G583.00	Heart failure with normal ejection fraction	1					
101137	G583.11	HFNEF - heart failure with normal ejection fraction	1					
106897	G583.12	Heart failure with preserved ejection fraction	1					
104275	G584.00	Right ventricular failure	1					
4024	G58z.00	Heart failure NOS	1					
17278	G58z.12	Cardiac failure NOS	1					
8966	G5yy900	Left ventricular systolic dysfunction	1					
12550	G5yyA00	Left ventricular diastolic dysfunction	1					
104876	G5yyB00	Right ventricular diastolic dysfunction	1					

107397	G5yyD00	Left ventricular cardiac dysfunction	1				
108180	G5yyE00	Right ventricular systolic dysfunction	1				
20822	Q48y100	Congenital cardiac failure	1				
20324	R2y1000	[D]Cardiorespiratory failure	1				
41577	1477	H/O: cerebrovascular disease		1			
19477	E004.00	Arteriosclerotic dementia		1			
43089	E004000	Uncomplicated arteriosclerotic dementia		1			
56912	E004100	Arteriosclerotic dementia with delirium		1			
55467	E004200	Arteriosclerotic dementia with paranoia		1			
43292	E004300	Arteriosclerotic dementia with depression		1			
42279	E004z00	Arteriosclerotic dementia NOS		1			
9565	Eu01.11	[X]Arteriosclerotic dementia		1			
2418	G6...00	Cerebrovascular disease		1			
45781	G63..00	Precerebral arterial occlusion		1			
63830	G63..12	Stenosis of precerebral arteries		1			
32447	G630.00	Basilar artery occlusion		1			
4240	G631.00	Carotid artery occlusion		1			
2156	G631.11	Stenosis, carotid artery		1			
4152	G631.12	Thrombosis, carotid artery		1			
40847	G632.00	Vertebral artery occlusion		1			
98642	G633.00	Multiple and bilateral precerebral arterial occlusion		1			
2652	G634.00	Carotid artery stenosis		1			
51326	G63y.00	Other precerebral artery occlusion		1			
71585	G63z.00	Precerebral artery occlusion NOS		1			
8837	G64..00	Cerebral arterial occlusion		1			
16517	G640.00	Cerebral thrombosis		1			
15019	G641.00	Cerebral embolism		1			
34758	G641.11	Cerebral embolus		1			
18689	G660.00	Middle cerebral artery syndrome		1			
19280	G661.00	Anterior cerebral artery syndrome		1			
19260	G662.00	Posterior cerebral artery syndrome		1			
13577	G67..00	Other cerebrovascular disease		1			
24385	G671100	Chronic cerebral ischaemia		1			

12555	G671z00	Generalised ischaemic cerebrovascular disease NOS		1			
31704	G677.00	Occlusion/stenosis cerebral arts not result cerebral infarct		1			
51759	G677000	Occlusion and stenosis of middle cerebral artery		1			
57527	G677100	Occlusion and stenosis of anterior cerebral artery		1			
65770	G677200	Occlusion and stenosis of posterior cerebral artery		1			
55602	G677300	Occlusion and stenosis of cerebellar arteries		1			
71274	G677400	Occlusion+stenosis of multiple and bilat cerebral arteries		1			
9943	G678.00	Cereb autosom dominant arteriop subcort infarcts leukoenceph		1			
98188	G679.00	Small vessel cerebrovascular disease		1			
34117	G67y.00	Other cerebrovascular disease OS		1			
37493	G67z.00	Other cerebrovascular disease NOS		1			
23361	G68..00	Late effects of cerebrovascular disease		1			
51138	G68W.00	Sequelae/other + unspecified cerebrovascular diseases		1			
51311	G6y..00	Other specified cerebrovascular disease		1			
10062	G6z..00	Cerebrovascular disease NOS		1			
37199	G70y000	Carotid artery atherosclerosis		1			
22677	G70y011	Carotid artery disease		1			
73901	Gyu6.00	[X]Cerebrovascular diseases		1			
109679	5C13.00	Old cerebral infarction on imaging		1			1
39403	G683.00	Sequelae of cerebral infarction		1			1
57495	G63..11	Infarction - precerebral		1			1
23671	G63y000	Cerebral infarct due to thrombosis of precerebral arteries		1			1
24446	G63y100	Cerebral infarction due to embolism of precerebral arteries		1			1
5363	G64..11	CVA - cerebral artery occlusion		1			1
569	G64..12	Infarction - cerebral		1			1
6155	G64..13	Stroke due to cerebral arterial occlusion		1			1
36717	G640000	Cerebral infarction due to thrombosis of cerebral arteries		1			1
27975	G641000	Cerebral infarction due to embolism of cerebral arteries		1			1
3149	G64z.00	Cerebral infarction NOS		1			1
15252	G64z.11	Brainstem infarction NOS		1			1
5602	G64z.12	Cerebellar infarction		1			1
25615	G64z000	Brainstem infarction		1			1
9985	G64z200	Left sided cerebral infarction		1			1

10504	G64z300	Right sided cerebral infarction		1			1
26424	G64z400	Infarction of basal ganglia		1			1
39344	G676000	Cereb infarct due cerebral venous thrombosis, nonpyogenic		1			1
101733	G67A.00	Cerebral vein thrombosis		1			1
40758	G6W..00	Cereb infarct due unsp occlus/stenos precerebr arteries		1			1
33543	G6X..00	Cerebrl infarctn due/unspcf occlusn or sten/cerebrl artrs		1			1
91627	Gyu6300	[X]Cerebrl infarctn due/unspcf occlusn or sten/cerebrl artrs		1			1
53745	Gyu6400	[X]Other cerebral infarction		1			1
90572	Gyu6500	[X]Occlusion and stenosis of other precerebral arteries		1			1
92036	Gyu6600	[X]Occlusion and stenosis of other cerebral arteries		1			1
94482	Gyu6G00	[X]Cereb infarct due unsp occlus/stenos precerebr arteries		1			1
33650	7929100	Percut transluminal coronary thrombolysis with streptokinase	1			1	
40996	7929111	Percut translum coronary thrombolytic therapy- streptokinase	1			1	
94504	8B3a.00	Door to needle time	1			1	
70440	8B3g.00	Pain to thrombolysis time	1			1	
35674	14A3.00	H/O: myocardial infarct <60	1			1	
40399	14A4.00	H/O: myocardial infarct >60	1			1	
50372	14AH.00	H/O: Myocardial infarction in last year	1			1	
100139	14AT.00	History of myocardial infarction	1			1	
23579	G310.00	Postmyocardial infarction syndrome	1			1	
4017	G32..00	Old myocardial infarction	1			1	
16408	G32..11	Healed myocardial infarction	1			1	
17464	G32..12	Personal history of myocardial infarction	1			1	
9555	G33z500	Post infarct angina	1			1	
36423	G36..00	Certain current complication follow acute myocardial infarct	1			1	
24126	G360.00	Haemopericardium/current comp folow acut myocard infarct	1			1	
23708	G361.00	Atrial septal defect/curr comp folow acut myocardal infarct	1			1	
37657	G362.00	Ventric septal defect/curr comp fol acut myocardal infarctn	1			1	
59189	G363.00	Ruptur cardiac wall w'out haemopericard/cur comp fol ac MI	1			1	
59940	G364.00	Ruptur chordae tendinae/curr comp fol acute myocard infarct	1			1	
69474	G365.00	Rupture papillary muscle/curr comp fol acute myocard infarct	1			1	
29553	G366.00	Thrombosis atrium,auric append&vent/curr comp foll acute MI	1			1	
35119	G501.00	Post infarction pericarditis	1			1	

61670	889A.00	Diab mellit insulin-glucose infus acute myocardial infarct	1			1	
241	G30..00	Acute myocardial infarction	1			1	
13566	G30..11	Attack - heart	1			1	
2491	G30..12	Coronary thrombosis	1			1	
30421	G30..13	Cardiac rupture following myocardial infarction (MI)	1			1	
1204	G30..14	Heart attack	1			1	
1677	G30..15	MI - acute myocardial infarction	1			1	
13571	G30..16	Thrombosis - coronary	1			1	
17689	G30..17	Silent myocardial infarction	1			1	
12139	G300.00	Acute anterolateral infarction	1			1	
5387	G301.00	Other specified anterior myocardial infarction	1			1	
40429	G301000	Acute anteroapical infarction	1			1	
17872	G301100	Acute anteroseptal infarction	1			1	
14897	G301z00	Anterior myocardial infarction NOS	1			1	
8935	G302.00	Acute inferolateral infarction	1			1	
29643	G303.00	Acute inferoposterior infarction	1			1	
23892	G304.00	Posterior myocardial infarction NOS	1			1	
14898	G305.00	Lateral myocardial infarction NOS	1			1	
63467	G306.00	True posterior myocardial infarction	1			1	
3704	G307.00	Acute subendocardial infarction	1			1	
9507	G307000	Acute non-Q wave infarction	1			1	
1678	G308.00	Inferior myocardial infarction NOS	1			1	
30330	G309.00	Acute Q-wave infarct	1			1	
17133	G30A.00	Mural thrombosis	1			1	
32854	G30B.00	Acute posterolateral myocardial infarction	1			1	
29758	G30X.00	Acute transmural myocardial infarction of unspecif site	1			1	
34803	G30y.00	Other acute myocardial infarction	1			1	
28736	G30y000	Acute atrial infarction	1			1	
62626	G30y100	Acute papillary muscle infarction	1			1	
41221	G30y200	Acute septal infarction	1			1	
46017	G30yz00	Other acute myocardial infarction NOS	1			1	
14658	G30z.00	Acute myocardial infarction NOS	1			1	
15661	G310.11	Dressler's syndrome	1			1	

68357	G31y100	Microinfarction of heart	1			1	
18842	G35..00	Subsequent myocardial infarction	1			1	
45809	G350.00	Subsequent myocardial infarction of anterior wall	1			1	
38609	G351.00	Subsequent myocardial infarction of inferior wall	1			1	
72562	G353.00	Subsequent myocardial infarction of other sites	1			1	
46166	G35X.00	Subsequent myocardial infarction of unspecified site	1			1	
32272	G38..00	Postoperative myocardial infarction	1			1	
46112	G380.00	Postoperative transmural myocardial infarction anterior wall	1			1	
46276	G381.00	Postoperative transmural myocardial infarction inferior wall	1			1	
106812	G383.00	Postoperative transmural myocardial infarction unspec site	1			1	
41835	G384.00	Postoperative subendocardial myocardial infarction	1			1	
68748	G38z.00	Postoperative myocardial infarction, unspecified	1			1	
96838	Gyu3400	[X]Acute transmural myocardial infarction of unspecif site	1			1	
109035	Gyu3500	[X]Subsequent myocardial infarction of other sites	1			1	
99991	Gyu3600	[X]Subsequent myocardial infarction of unspecified site	1			1	
10562	G307100	Acute non-ST segment elevation myocardial infarction	1			1	
19825	5593	Femoral arteriogram abnormal			1		
41825	55A2.00	Lower limb arteriogram abnorm.			1		
59534	14NB.00	H/O: Peripheral vascular disease procedure			1		
109034	1M11000	Ischaemic foot pain at rest			1		
109217	1M11100	Ischaemic foot pain when walking			1		
9561	2G63.00	Ischaemic toe			1		
35399	C107.00	Diabetes mellitus with peripheral circulatory disorder			1		
32403	C107.11	Diabetes mellitus with gangrene			1		
32556	C107.12	Diabetes with gangrene			1		
70448	C107000	Diabetes mellitus, juvenile +peripheral circulatory disorder			1		
63357	C107100	Diabetes mellitus, adult, + peripheral circulatory disorder			1		
33807	C107200	Diabetes mellitus, adult with gangrene			1		
69124	C107300	IDDM with peripheral circulatory disorder			1		
56803	C107400	NIDDM with peripheral circulatory disorder			1		
65025	C107z00	Diabetes mellitus NOS with peripheral circulatory disorder			1		
60499	C108600	Insulin dependent diabetes mellitus with gangrene			1		
64446	C108G00	Insulin dependent diab mell with peripheral angiopathy			1		

40401	C109500	Non-insulin dependent diabetes mellitus with gangrene			1		
62107	C109511	Type II diabetes mellitus with gangrene			1		
46150	C109512	Type 2 diabetes mellitus with gangrene			1		
54212	C109F00	Non-insulin-dependent d m with peripheral angiopath			1		
54899	C109F11	Type II diabetes mellitus with peripheral angiopathy			1		
60699	C109F12	Type 2 diabetes mellitus with peripheral angiopathy			1		
69993	C10E600	Type 1 diabetes mellitus with gangrene			1		
102112	C10E611	Type I diabetes mellitus with gangrene			1		
109051	C10E612	Insulin dependent diabetes mellitus with gangrene			1		
93468	C10EG00	Type 1 diabetes mellitus with peripheral angiopathy			1		
12736	C10F500	Type 2 diabetes mellitus with gangrene			1		
104323	C10F511	Type II diabetes mellitus with gangrene			1		
37806	C10FF00	Type 2 diabetes mellitus with peripheral angiopathy			1		
104639	C10FF11	Type II diabetes mellitus with peripheral angiopathy			1		
14797	G702.00	Extremity artery atheroma			1		
16260	G702z00	Extremity artery atheroma NOS			1		
5943	G73..00	Other peripheral vascular disease			1		
5702	G73..11	Peripheral ischaemic vascular disease			1		
1826	G73..12	Ischaemia of legs			1		
6827	G73..13	Peripheral ischaemia			1		
98174	G733.00	Ischaemic foot			1		
105317	G734.00	Peripheral arterial disease			1		
38907	G73y.00	Other specified peripheral vascular disease			1		
34152	G73y000	Diabetic peripheral angiopathy			1		
23871	G73y100	Peripheral angiopathic disease EC NOS			1		
4325	G73yz00	Other specified peripheral vascular disease NOS			1		
3530	G73z.00	Peripheral vascular disease NOS			1		
1517	G73z000	Intermittent claudication			1		
6853	G73z011	Claudication			1		
101866	G73z012	Vascular claudication			1		
2760	G73zz00	Peripheral vascular disease NOS			1		
15302	G742z00	Peripheral arterial embolism and thrombosis NOS			1		
73961	Gyu7400	[X]Other specified peripheral vascular diseases			1		

6308	M271.12	Ischaemic leg ulcer			1		
24327	M271000	Ischaemic ulcer diabetic foot			1		
53634	R054200	[D]Gangrene of toe in diabetic			1		
31053	R054300	[D]Widespread diabetic foot gangrene			1		
95573	7A10000	Emerg aortic bypass by anastomosis axillary to femoral art			1		
23352	7A10100	Bypass aorta by anastomosis axillary to femoral artery NEC			1		
34153	7A10200	Axillo-bifemoral bypass graft			1		
40302	7A10300	Axillo-unifemoral PTFE bypass graft			1		
36952	7A12.00	Other bypass of bifurcation of aorta			1		
48755	7A12000	Emerg bypass bifurc aorta by anast aorta to femoral artery			1		
2761	7A12100	Bypass bifurc aorta by anastom aorta to femoral artery NEC			1		
14895	7A12111	Aorto bifemoral graft			1		
28166	7A12112	Dacron aortofemoral Y graft			1		
15532	7A12300	Bypass bifurcation aorta by anastom aorta to iliac artery			1		
3778	7A12311	Aorto biiliac graft			1		
42465	7A12312	Dacron aortoiliac Y graft			1		
55825	7A12y00	Other specified other bypass of bifurcation of aorta			1		
64798	7A12z00	Other bypass of bifurcation of aorta NOS			1		
55402	7A19200	Open embolectomy of bifurcation of aorta			1		
21927	7A41.00	Other bypass of iliac artery			1		
101910	7A41.11	Other bypass of iliac artery by anastomosis			1		
44250	7A41000	Emerg bypass iliac art by iliac/femoral art anastomosis NEC			1		
28616	7A41100	Bypass iliac artery by iliac/femoral artery anastomosis NEC			1		
72448	7A41200	Emerg bypass iliac artery by femoral/femoral art anast NEC			1		
43648	7A41211	Emergency femoro-femoral prosthetic cross over graft			1		
36443	7A41300	Bypass iliac artery by femoral/femoral art anastomosis NEC			1		
30989	7A41311	Femoro-femoral prosthetic cross over graft			1		
68141	7A41400	Emerg bypass comm iliac art by aorta/com iliac art anast NEC			1		
66917	7A41600	Emerg bypass leg artery by aorta/com fem art anastomosis NEC			1		
32492	7A41900	Bypass common iliac artery by aorta/com iliac art anast NEC			1		
55554	7A41B00	Bypass leg artery by aorta/com femoral art anastomosis NEC			1		
66804	7A41C00	Bypass leg artery by aorta/deep femoral art anastomosis NEC			1		
100036	7A41D00	Bypass iliac artery by iliac/iliac artery anastomosis NEC			1		

41768	7A41F00	Ilio-femoral prosthetic cross over graft			1		
52357	7A41y00	Other specified other bypass of iliac artery			1		
38921	7A41z00	Other bypass of iliac artery NOS			1		
39039	7A42.00	Reconstruction of iliac artery			1		
63280	7A42.11	Reconstruction of common iliac artery			1		
62818	7A42000	Endarterectomy and patch repair of iliac artery			1		
36065	7A42011	Endarterectomy and patch repair of common iliac artery			1		
59602	7A42012	Iliac endarterectomy and patch			1		
51211	7A42100	Endarterectomy of iliac artery NEC			1		
44430	7A42111	Endarterectomy of common iliac artery NEC			1		
50894	7A42y00	Other specified reconstruction of iliac artery			1		
62866	7A42z00	Reconstruction of iliac artery NOS			1		
51331	7A43.00	Other open operations on iliac artery			1		
53580	7A43.11	Other open operations on common iliac artery			1		
61256	7A43000	Repair of iliac artery NEC			1		
66437	7A43011	Repair of common iliac artery NEC			1		
61255	7A43100	Open embolectomy of iliac artery			1		
47562	7A43111	Open embolectomy of common iliac artery			1		
34037	7A43300	Open insertion of iliac artery stent			1		
10827	7A44000	Percutaneous transluminal angioplasty of iliac artery			1		
66930	7A44100	Percutaneous transluminal embolectomy of iliac artery			1		
7111	7A44300	Insertion of iliac artery stent			1		
24229	7A44400	Percutaneous transluminal insertion of iliac artery stent			1		
28894	7A44y00	Other specified transluminal operation on iliac artery			1		
55877	7A44z00	Transluminal operation on iliac artery NOS			1		
9099	7A47.00	Other emergency bypass of femoral artery or popliteal artery			1		
72491	7A47.11	Other emerg bypass femoral or popliteal art by anastomosis			1		
100113	7A47.12	Other emergency bypass of common femoral artery			1		
63238	7A47.13	Other emergency bypass of deep femoral artery			1		
39776	7A47.14	Other emergency bypass of popliteal artery			1		
97606	7A47.15	Other emergency bypass of superficial femoral artery			1		
11766	7A47.16	Other emergency bypass of femoral artery			1		
43651	7A47000	Emerg bypass femoral art by fem/pop art anast c prosth NEC			1		

67818	7A47100	Emerg bypass popliteal art by pop/pop art anast c prosth NEC			1		
52342	7A47200	Emerg bypass femoral art by fem/pop a anast c vein graft NEC			1		
60693	7A47300	Emerg bypass pop art by pop/pop art anast c vein graft NEC			1		
66820	7A47400	Emerg bypass femoral art by fem/tib art anast c prosth NEC			1		
96255	7A47600	Emerg bypass femoral art by fem/tib a anast c vein graft NEC			1		
66879	7A47700	Emerg bypass pop art by pop/tib art anast c vein graft NEC			1		
99676	7A47800	Emerg bypass femoral art by fem/peron art anast c prosth NEC			1		
62775	7A47B00	Emerg bypass pop art by pop/peron art anast c vein graft NEC			1		
48939	7A47C00	Emerg bypass femoral artery by fem/fem art anastomosis NEC			1		
70922	7A47D00	Emerg bypass popliteal artery by pop/fem art anastomosis NEC			1		
65692	7A47y00	Other emergency bypass of femoral or popliteal artery OS			1		
68320	7A47z00	Other emergency bypass of femoral or popliteal artery NOS			1		
24692	7A48.00	Other bypass of femoral artery or popliteal artery			1		
61974	7A48.11	Other bypass of femoral or popliteal artery by anastomosis			1		
37787	7A48.12	Other bypass of common femoral artery			1		
18060	7A48.14	Other bypass of femoral artery			1		
12331	7A48.15	Other bypass of popliteal artery			1		
40732	7A48.16	Other bypass of superficial femoral artery			1		
27580	7A48000	Bypass femoral artery by fem/pop art anast c prosthesis NEC			1		
64555	7A48100	Bypass popliteal artery by pop/pop a anast c prosthesis NEC			1		
28030	7A48200	Bypass femoral artery by fem/pop art anast c vein graft NEC			1		
24097	7A48300	Bypass popliteal artery by pop/pop a anast c vein graft NEC			1		
39877	7A48400	Bypass femoral artery by fem/tib art anast c prosthesis NEC			1		
60465	7A48500	Bypass popliteal artery by pop/tib a anast c prosthesis NEC			1		
41823	7A48600	Bypass femoral artery by fem/tib art anast c vein graft NEC			1		
48700	7A48700	Bypass popliteal artery by pop/tib a anast c vein graft NEC			1		
67982	7A48800	Bypass femoral artery by fem/peron a anast c prosthesis NEC			1		
107158	7A48900	Bypass popliteal artery by pop/peron art anast c prosth NEC			1		
53675	7A48A00	Bypass femoral artery by fem/peron a anast c vein graft NEC			1		
68412	7A48B00	Bypass popliteal art by pop/peron art anast c vein graft NEC			1		
45428	7A48C00	Bypass femoral artery by femoral/femoral art anastomosis NEC			1		
42115	7A48D00	Bypass popliteal artery by pop/fem artery anastomosis NEC			1		
22016	7A48E00	Femoro-femoral prosthetic cross over graft			1		

42640	7A48y00	Other bypass of femoral artery or popliteal artery OS			1		
2066	7A48z00	Other bypass of femoral artery or popliteal artery NOS			1		
31338	7A49.00	Reconstruction of femoral artery or popliteal artery			1		
73822	7A49.11	Reconstruction of common femoral artery			1		
63396	7A49.13	Reconstruction of femoral artery			1		
28125	7A49.14	Reconstruction of popliteal artery			1		
96809	7A49.15	Reconstruction of superficial femoral artery			1		
28777	7A49000	Endarterectomy and patch repair of femoral artery			1		
52695	7A49100	Endarterectomy and patch repair of popliteal artery			1		
18816	7A49200	Endarterectomy of femoral artery NEC			1		
36136	7A49300	Endarterectomy of popliteal artery NEC			1		
57793	7A49400	Profundoplasty femoral artery & patch repair deep fem artery			1		
63605	7A49500	Profundoplasty and patch repair of popliteal artery			1		
47835	7A49600	Profundoplasty of femoral artery NEC			1		
65669	7A49700	Profundoplasty of popliteal artery NEC			1		
52869	7A49800	Reconstruction of femoral artery with vein graft			1		
69519	7A49900	Reconstruction of popliteal artery with vein graft			1		
67083	7A49y00	Reconstruction of femoral or popliteal artery OS			1		
6617	7A49z00	Reconstruction of femoral or popliteal artery NOS			1		
40397	7A4A.00	Other open operations on femoral artery or popliteal artery			1		
60212	7A4A.11	Other open operations on common femoral artery			1		
20892	7A4A.13	Other open operations on popliteal artery			1		
49319	7A4A.14	Other open operations on superficial femoral artery			1		
33555	7A4A000	Repair of femoral artery NEC			1		
28119	7A4A100	Repair of popliteal artery NEC			1		
9119	7A4A200	Open embolectomy of femoral artery			1		
16363	7A4A211	Open thrombectomy of femoral artery			1		
17336	7A4A212	Open femoral embolectomy			1		
39437	7A4A300	Open embolectomy popliteal artery			1		
42645	7A4A311	Open thrombectomy of popliteal artery			1		
63368	7A4A700	Repair of femoral artery with temporary silastic shunt			1		
66869	7A4A800	Repair of popliteal artery with temporary silastic shunt			1		
24677	7A4Ay00	Other open operation on femoral or popliteal artery OS			1		

6356	7A4B000	Percutaneous transluminal angioplasty of femoral artery			1		
29112	7A4B100	Percutaneous transluminal angioplasty of popliteal artery			1		
6256	7A4B200	Percutaneous transluminal embolectomy of femoral artery			1		
52289	7A4B300	Percutaneous transluminal embolectomy of popliteal artery			1		
71041	7A4B400	Percutaneous transluminal embolisation of femoral artery			1		
40619	7A4B500	Percutaneous transluminal embolisation of popliteal artery			1		
18030	7A4B800	Percut translum thrombolysis femoral graft streptokinase			1		
81445	7A4B900	Percutaneous transluminal insertion of stent femoral artery			1		
20657	7A50.00	Revision of reconstruction of artery			1		
54071	7A50000	Revision of reconstruction involving aorta			1		
49273	7A50100	Revision of reconstruction involving iliac artery			1		
18038	7A50200	Revision of reconstruction involving femoral artery			1		
65286	7A50300	Revision of reconstruction of popliteal artery			1		
12229	G30X000	Acute ST segment elevation myocardial infarction	1			1	
34135	14A7.00	H/O: CVA/stroke		1			1
6305	14A7.11	H/O: CVA		1			1
5871	14A7.12	H/O: stroke		1			1
66873	14AK.00	H/O: Stroke in last year		1			1
100639	1M4..00	Central post-stroke pain		1			1
107195	661M700	Stroke self-management plan agreed		1			1
109743	661N700	Stroke self-management plan review		1			1
105100	662M100	Stroke 6 month review		1			1
18686	662e.00	Stroke/CVA annual review		1			1
107886	662e.11	Stroke annual review		1			1
55351	7P24200	Delivery of rehabilitation for stroke		1			1
56458	8HHM.00	Ref to multidisciplinary stroke function improvement service		1			1
104638	8IEC.00	Ref multidisciplinary stroke function improvement declined		1			1
31218	9Om..00	Stroke/transient ischaemic attack monitoring administration		1			1
28753	9Om0.00	Stroke/transient ischaemic attack monitoring first letter		1			1
34245	9Om1.00	Stroke/transient ischaemic attack monitoring second letter		1			1
34375	9Om2.00	Stroke/transient ischaemic attack monitoring third letter		1			1
51465	9Om3.00	Stroke/transient ischaemic attack monitoring verbal invitati		1			1
89913	9Om4.00	Stroke/transient ischaemic attack monitoring telephone invte		1			1

6228	G68X.00	Sequelae of stroke,not specfd as h'morrhage or infarction		1				1
110337	Gyu6C00	[X]Sequelae of stroke,not specfd as h'morrhage or infarction		1				1
19348	ZV12511	[V]Personal history of stroke		1				1
7138	ZV12512	[V]Personal history of cerebrovascular accident (CVA)		1				1
104505	662M200	Stroke initial post discharge review		1				1
93459	Fyu5600	[X]Other lacunar syndromes		1				1
47642	G64z100	Wallenberg syndrome		1				1
5185	G64z111	Lateral medullary syndrome		1				1
1469	G66..00	Stroke and cerebrovascular accident unspecified		1				1
1298	G66..11	CVA unspecified		1				1
6253	G66..12	Stroke unspecified		1				1
6116	G66..13	CVA - Cerebrovascular accident unspecified		1				1
8443	G663.00	Brain stem stroke syndrome		1				1
17322	G664.00	Cerebellar stroke syndrome		1				1
33499	G665.00	Pure motor lacunar syndrome		1				1
51767	G666.00	Pure sensory lacunar syndrome		1				1
7780	G667.00	Left sided CVA		1				1
12833	G668.00	Right sided CVA		1				1
47607	L440.11	CVA - cerebrovascular accident in the puerperium		1				1
56279	L440.12	Stroke in the puerperium		1				1
21195	G575100	Sudden cardiac death, so described	1					
13567	14AB.00	H/O: TIA		1				
101251	ZV12D00	[V]Personal history of transient ischaemic attack		1				
100015	8CRB.00	Transient ischaemic attack clinical management plan		1				
63746	Fyu5500	[X]Other transnt cerebral ischaemic attacks+related syndroms		1				
504	G65..00	Transient cerebral ischaemia		1				
1433	G65..12	Transient ischaemic attack		1				
105738	G657.00	Carotid territory transient ischaemic attack		1				
19354	G65y.00	Other transient cerebral ischaemia		1				
1895	G65z.00	Transient cerebral ischaemia NOS		1				
15788	G65zz00	Transient cerebral ischaemia NOS		1				
97001	44p2.00	Cardiac troponin positive	1			1		
29300	662K300	Angina control - worsening	1					

36523	G311.00	Preinfarction syndrome	1			1	
4656	G311.11	Crescendo angina	1			1	
39655	G311.12	Impending infarction	1			1	
1431	G311.13	Unstable angina	1			1	
19655	G311.14	Angina at rest	1			1	
7347	G311100	Unstable angina	1			1	
17307	G311200	Angina at rest	1			1	
34328	G311300	Refractory angina	1				
18118	G311400	Worsening angina	1				
54251	G311z00	Preinfarction syndrome NOS	1			1	
39449	G312.00	Coronary thrombosis not resulting in myocardial infarction	1			1	
9276	G31y000	Acute coronary insufficiency	1			1	
66388	G33z000	Status anginosus	1			1	

ICD10 code list for cardiovascular disease

- any_chd: any code indicating coronary heart disease; including stable angina, percutaneous coronary intervention, coronary artery bypass graft, myocardial infarction, acute coronary syndrome, unstable angina, heart failure, sudden cardiac death, coronary thrombolysis, coronary heart disease not otherwise specified.
- any_cvd: any code indicating cerebrovascular disease; including stroke and non-stroke cerebrovascular disease, plus procedures to repair cerebrovascular disease.
- any_pad: any code indicating peripheral arterial disease; including peripheral arterial disease, abdominal aortic aneurysm, procedures to repair these.
- any_acs: any code indicating unstable angina; acute coronary syndrome, myocardial infarction, coronary thrombolysis.
- any_stroke: any code indicating ischaemic, haemorrhagic stroke or stroke not otherwise specified.

icd	alt_code	description	any_chd	any_cvd	any_pad	any_acs	any_stroke
I20	I20	Angina pectoris	1				
I20.1	I201	Angina pectoris with documented spasm	1				
I20.8	I208	Other forms of angina pectoris	1				
I20.9	I209	Angina pectoris, unspecified	1				
Z95.5	Z955	Presence of coronary angioplasty implant and graft	1				
I71	I71	Aortic aneurysm and dissection			1		
I71.3	I713	Abdominal aortic aneurysm, ruptured			1		
I71.4	I714	Abdominal aortic aneurysm, without mention of rupture			1		
I71.5	I715	Thoracoabdominal aortic aneurysm, ruptured			1		
I71.6	I716	Thoracoabdominal aortic aneurysm, without mention of rupture			1		
I71.8	I718	Aortic aneurysm of unspecified site, ruptured			1		
I71.9	I719	Aortic aneurysm of unspecified site, without mention of rupture			1		
I79.0	I790	Aneurysm of aorta in diseases classified elsewhere			1		
I25	I25	Chronic ischaemic heart disease	1				
I25.0	I250	Atherosclerotic cardiovascular disease, so described	1				
I25.1	I251	Atherosclerotic heart disease	1				
I25.3	I253	Aneurysm of heart	1				
I25.4	I254	Coronary artery aneurysm	1				
I25.5	I255	Ischaemic cardiomyopathy	1				
I25.6	I256	Silent myocardial ischaemia	1				
I25.8	I258	Other forms of chronic ischaemic heart disease	1				

I25.9	I259	Chronic ischaemic heart disease, unspecified	1				
I11.0	I110	Hypertensive heart disease with (congestive) heart failure	1				
I13.0	I130	Hypertensive heart and renal disease with (congestive) heart failure	1				
I13.2	I132	Hypertensive heart and renal disease with both (congestive) heart failure and renal failure	1				
I26.0	I260	Pulmonary embolism with mention of acute cor pulmonale	1				
I50	I50	Heart failure	1				
I50.0	I500	Congestive heart failure	1				
I50.1	I501	Left ventricular failure	1				
I50.9	I509	Heart failure, unspecified	1				
I21	I21	Acute myocardial infarction	1			1	
I21.0	I210	Acute transmural myocardial infarction of anterior wall	1			1	
I21.1	I211	Acute transmural myocardial infarction of inferior wall	1			1	
I21.2	I212	Acute transmural myocardial infarction of other sites	1			1	
I21.3	I213	Acute transmural myocardial infarction of unspecified site	1			1	
I21.4	I214	Acute subendocardial myocardial infarction	1			1	
I21.9	I219	Acute myocardial infarction, unspecified	1			1	
I22	I22	Subsequent myocardial infarction	1			1	
I22.0	I220	Subsequent myocardial infarction of anterior wall	1			1	
I22.1	I221	Subsequent myocardial infarction of inferior wall	1			1	
I22.8	I228	Subsequent myocardial infarction of other sites	1			1	
I22.9	I229	Subsequent myocardial infarction of unspecified site	1			1	
I23	I23	Certain current complications following acute myocardial infarction	1			1	
I23.0	I230	Haemopericardium as current complication following acute myocardial infarction	1			1	
I23.1	I231	Atrial septal defect as current complication following acute myocardial infarction	1			1	
I23.2	I232	Ventricular septal defect as current complication following acute myocardial infarction	1			1	
I23.3	I233	Rupture of cardiac wall without haemopericardium as current complication following acute myocardial infarction	1			1	
I23.4	I234	Rupture of chordae tendineae as current complication following acute myocardial infarction	1			1	
I23.5	I235	Rupture of papillary muscle as current complication following acute myocardial infarction	1			1	
I23.6	I236	Thrombosis of atrium, auricular appendage, and ventricle as current complications following acute myocardial infarction	1			1	
I23.8	I238	Other current complications following acute myocardial infarction	1			1	

I24.1	I241	Dressler's syndrome	1			1	
I25.2	I252	Old myocardial infarction	1			1	
I70.2	I702	Atherosclerosis of arteries of extremities			1		
I70.20	I7020	Atherosclerosis of arteries of extremities			1		
I70.21	I7021	Atherosclerosis of arteries of extremities			1		
I73	I73	Other peripheral vascular diseases			1		
I73.8	I738	Other specified peripheral vascular diseases			1		
I73.9	I739	Peripheral vascular disease, unspecified			1		
I79.2	I792	Peripheral angiopathy in diseases classified elsewhere			1		
I63	I63	Cerebral infarction		1			1
I63.0	I630	Cerebral infarction due to thrombosis of precerebral arteries		1			1
I63.1	I631	Cerebral infarction due to embolism of precerebral arteries		1			1
I63.2	I632	Cerebral infarction due to unspecified occlusion or stenosis of precerebral arteries		1			1
I63.3	I633	Cerebral infarction due to thrombosis of cerebral arteries		1			1
I63.4	I634	Cerebral infarction due to embolism of cerebral arteries		1			1
I63.5	I635	Cerebral infarction due to unspecified occlusion or stenosis of cerebral arteries		1			1
I63.6	I636	Cerebral infarction due to cerebral venous thrombosis, nonpyogenic		1			1
I63.8	I638	Other cerebral infarction		1			1
I63.9	I639	Cerebral infarction, unspecified		1			1
I69.3	I693	Sequelae of cerebral infarction		1			1
I60	I60	Subarachnoid haemorrhage		1			1
I60.0	I600	Subarachnoid haemorrhage from carotid siphon and bifurcation		1			1
I60.1	I601	Subarachnoid haemorrhage from middle cerebral artery		1			1
I60.2	I602	Subarachnoid haemorrhage from anterior communicating artery		1			1
I60.3	I603	Subarachnoid haemorrhage from posterior communicating artery		1			1
I60.4	I604	Subarachnoid haemorrhage from basilar artery		1			1
I60.5	I605	Subarachnoid haemorrhage from vertebral artery		1			1
I60.6	I606	Subarachnoid haemorrhage from other intracranial arteries		1			1
I60.7	I607	Subarachnoid haemorrhage from intracranial artery, unspecified		1			1
I60.8	I608	Other subarachnoid haemorrhage		1			1

I60.9	I609	Subarachnoid haemorrhage, unspecified		1			1
I61	I61	Intracerebral haemorrhage		1			1
I61.0	I610	Intracerebral haemorrhage in hemisphere, subcortical		1			1
I61.1	I611	Intracerebral haemorrhage in hemisphere, cortical		1			1
I61.2	I612	Intracerebral haemorrhage in hemisphere, unspecified		1			1
I61.3	I613	Intracerebral haemorrhage in brain stem		1			1
I61.4	I614	Intracerebral haemorrhage in cerebellum		1			1
I61.5	I615	Intracerebral haemorrhage, intraventricular		1			1
I61.6	I616	Intracerebral haemorrhage, multiple localized		1			1
I61.8	I618	Other intracerebral haemorrhage		1			1
I61.9	I619	Intracerebral haemorrhage, unspecified		1			1
I62	I62	Other nontraumatic intracranial haemorrhage		1			1
I62.0	I620	Subdural haemorrhage (acute)(nontraumatic)		1			1
I62.1	I621	Nontraumatic extradural haemorrhage		1			1
I62.9	I629	Intracranial haemorrhage (nontraumatic), unspecified		1			1
I69.0	I690	Sequelae of subarachnoid haemorrhage		1			1
I69.1	I691	Sequelae of intracerebral haemorrhage		1			1
I69.2	I692	Sequelae of other nontraumatic intracranial haemorrhage		1			1
G45.0	G450	Vertebro-basilar artery syndrome		1			
G45.1	G451	Carotid artery syndrome (hemispheric)		1			
G45.2	G452	Multiple and bilateral precerebral artery syndromes		1			
G46	G46	Vascular syndromes of brain in cerebrovascular diseases		1			
G46.0	G460	Middle cerebral artery syndrome		1			
G46.1	G461	Anterior cerebral artery syndrome		1			
G46.2	G462	Posterior cerebral artery syndrome		1			
G46.3	G463	Brain stem stroke syndrome		1			
G46.4	G464	Cerebellar stroke syndrome		1			
G46.5	G465	Pure motor lacunar syndrome		1			
G46.6	G466	Pure sensory lacunar syndrome		1			
G46.7	G467	Other lacunar syndromes		1			

G46.8	G468	Other vascular syndromes of brain in cerebrovascular diseases		1			
I65	I65	Occlusion and stenosis of precerebral arteries, not resulting in cerebral infarction		1			
I65.0	I650	Occlusion and stenosis of vertebral artery		1			
I65.1	I651	Occlusion and stenosis of basilar artery		1			
I65.2	I652	Occlusion and stenosis of carotid artery		1			
I65.3	I653	Occlusion and stenosis of multiple and bilateral precerebral arteries		1			
I65.8	I658	Occlusion and stenosis of other precerebral artery		1			
I65.9	I659	Occlusion and stenosis of unspecified precerebral artery		1			
I66	I66	Occlusion and stenosis of cerebral arteries, not resulting in cerebral infarction		1			
I66.0	I660	Occlusion and stenosis of middle cerebral artery		1			
I66.1	I661	Occlusion and stenosis of anterior cerebral artery		1			
I66.2	I662	Occlusion and stenosis of posterior cerebral artery		1			
I66.3	I663	Occlusion and stenosis of cerebellar arteries		1			
I66.4	I664	Occlusion and stenosis of multiple and bilateral cerebral arteries		1			
I66.8	I668	Occlusion and stenosis of other cerebral artery		1			
I66.9	I669	Occlusion and stenosis of unspecified cerebral artery		1			
I67	I67	Other cerebrovascular diseases		1			
I67.2	I672	Cerebral atherosclerosis		1			
I67.8	I678	Other specified cerebrovascular diseases		1			
I67.9	I679	Cerebrovascular disease, unspecified		1			
I69	I69	Sequelae of cerebrovascular disease		1			
I69.8	I698	Sequelae of other and unspecified cerebrovascular diseases		1			
I72.5	I725	Aneurysm and dissection of other precerebral arteries		1			
I64	I64X	Stroke, not specified as haemorrhage or infarction		1			1
I69.4	I694	Sequelae of stroke, not specified as haemorrhage or infarction		1			1
I46.1	I461	Sudden cardiac death, so described	1				
G45	G45	Transient cerebral ischaemic attacks and related syndromes		1			
G45.8	G458	Other transient cerebral ischaemic attacks and related syndromes		1			
G45.9	G459	Transient cerebral ischaemic attack, unspecified		1			
I20.0	I200	Unstable angina	1			1	

I24	I24	Other acute ischaemic heart diseases	1			1	
I24.0	I240	Coronary thrombosis not resulting in myocardial infarction	1			1	
I24.8	I248	Other forms of acute ischaemic heart disease	1			1	
I24.9	I249	Acute ischaemic heart disease, unspecified	1			1	

OPCS code list for cardiovascular disease

- any_chd: any code indicating coronary heart disease; including percutaneous coronary intervention, coronary artery bypass graft, coronary thrombolysis.
- any_cvd: any code indicating stroke or procedures to repair cerebrovascular disease.
- any_pad: any code indicating procedures to repair peripheral arterial disease or abdominal aortic aneurysm.
- any_acs: any code indicating coronary thrombolysis.
- any_stroke: any code indicating ischaemic, haemorrhagic stroke or stroke not otherwise specified.

opcs_code	opcs_term	any_chd	any_cvd	any_pad	any_acs	any_stroke
L294	Endarterectomy of carotid artery and patch repair of carotid artery		1			
L295	Endarterectomy of carotid artery NEC		1			
L311	Percutaneous transluminal angioplasty of carotid artery		1			
L314	Percutaneous transluminal insertion of stent into carotid artery		1			
K40	Saphenous vein graft replacement of coronary artery	1				
K40.1	Saphenous vein graft replacement of one coronary artery	1				
K40.2	Saphenous vein graft replacement of two coronary arteries	1				
K40.3	Saphenous vein graft replacement of three coronary arteries	1				
K40.4	Saphenous vein graft replacement of four or more coronary arteries	1				
K40.8	Other specified saphenous vein graft replacement of coronary artery	1				
K40.9	Unspecified saphenous vein graft replacement of coronary artery	1				
K41	Other autograft replacement of coronary artery	1				
K41.1	Autograft replacement of one coronary artery NEC	1				
K41.2	Autograft replacement of two coronary arteries NEC	1				
K41.3	Autograft replacement of three coronary arteries NEC	1				
K41.4	Autograft replacement of four or more coronary arteries NEC	1				
K41.8	Other specified other autograft replacement of coronary artery	1				
K41.9	Unspecified other autograft replacement of coronary artery	1				
K42	Allograft replacement of coronary artery	1				
K42.1	Allograft replacement of one coronary artery	1				
K42.2	Allograft replacement of two coronary arteries	1				
K42.3	Allograft replacement of three coronary arteries	1				
K42.4	Allograft replacement of four or more coronary arteries	1				

K42.8	Other specified allograft replacement of coronary artery	1				
K42.9	Unspecified allograft replacement of coronary artery	1				
K43	Prosthetic replacement of coronary artery	1				
K43.1	Prosthetic replacement of one coronary artery	1				
K43.2	Prosthetic replacement of two coronary arteries	1				
K43.3	Prosthetic replacement of three coronary arteries	1				
K43.4	Prosthetic replacement of four or more coronary arteries	1				
K43.8	Other specified prosthetic replacement of coronary artery	1				
K43.9	Unspecified prosthetic replacement of coronary artery	1				
K44	Other replacement of coronary artery	1				
K44.1	Replacement of coronary arteries using multiple methods	1				
K44.2	Revision of replacement of coronary artery	1				
K44.8	Other specified other replacement of coronary artery	1				
K44.9	Unspecified other replacement of coronary artery	1				
K45	Connection of thoracic artery to coronary artery	1				
K45.1	Double anastomosis of mammary arteries to coronary arteries	1				
K45.2	Double anastomosis of thoracic arteries to coronary arteries NEC	1				
K45.3	Anastomosis of mammary artery to left anterior descending coronary artery	1				
K45.4	Anastomosis of mammary artery to coronary artery NEC	1				
K45.5	Anastomosis of thoracic artery to coronary artery NEC	1				
K45.6	Revision of connection of thoracic artery to coronary artery	1				
K45.8	Other specified connection of thoracic artery to coronary artery	1				
K45.9	Unspecified connection of thoracic artery to coronary artery	1				
K46	Other bypass of coronary artery	1				
K46.1	Double implantation of mammary arteries into heart	1				
K46.2	Double implantation of thoracic arteries into heart NEC	1				
K46.3	Implantation of mammary artery into heart NEC	1				
K46.4	Implantation of thoracic artery into heart NEC	1				
K46.5	Revision of implantation of thoracic artery into heart	1				
K46.8	Other specified other bypass of coronary artery	1				
K46.9	Unspecified other bypass of coronary artery	1				

		1				
K47.1	Endarterectomy of coronary artery	1				
K49	Transluminal balloon angioplasty of coronary artery	1				
K49.1	Percutaneous transluminal balloon angioplasty of one coronary artery	1				
K49.2	Percutaneous transluminal balloon angioplasty of multiple coronary arteries	1				
K49.3	Percutaneous transluminal balloon angioplasty of bypass graft of coronary artery	1				
K49.4	Percutaneous transluminal cutting balloon angioplasty of coronary artery	1				
K49.8	Other specified transluminal balloon angioplasty of coronary artery	1				
K49.9	Unspecified transluminal balloon angioplasty of coronary artery	1				
K50	Other therapeutic transluminal operations on coronary artery	1				
K50.1	Percutaneous transluminal laser coronary angioplasty	1				
K50.4	Percutaneous transluminal atherectomy of coronary artery	1				
K50.8	Other specified other therapeutic transluminal operations on coronary artery	1				
K50.9	Unspecified other therapeutic transluminal operations on coronary artery	1				
K75	Percutaneous transluminal balloon angioplasty and insertion of stent into coronary artery	1				
K75.1	Percutaneous transluminal balloon angioplasty and insertion of 1-2 drug-eluting stents into coronary artery	1				
K75.2	Percutaneous transluminal balloon angioplasty and insertion of 3 or more drug-eluting stents into coronary artery	1				
K75.3	Percutaneous transluminal balloon angioplasty and insertion of 1-2 stents into coronary artery	1				
K75.4	Percutaneous transluminal balloon angioplasty and insertion of 3 or more stents into coronary artery NEC	1				
K75.8	Other specified percutaneous transluminal balloon angioplasty and insertion of stent into coronary artery	1				
K75.9	Unspecified percutaneous transluminal balloon angioplasty and insertion of stent into coronary artery	1				
K50.2	Percutaneous transluminal coronary thrombolysis using streptokinase	1			1	
K50.3	Percutaneous transluminal injection of therapeutic substance into coronary artery NEC	1			1	
U54.3	Delivery of rehabilitation for stroke		1			1
L18	Emergency replacement of aneurysmal segment of aorta			1		
L18.4	Emergency replacement of aneurysmal segment of infrarenal abdominal aorta by anastomosis of aorta to aorta			1		
L18.5	Emergency replacement of aneurysmal segment of abdominal aorta by anastomosis of aorta to aorta NEC			1		
L18.8	Other specified emergency replacement of aneurysmal segment of aorta			1		
L18.9	Unspecified emergency replacement of aneurysmal segment of aorta			1		
L19	Other replacement of aneurysmal segment of aorta			1		
L19.3	Replacement of aneurysmal segment of suprarenal abdominal aorta by anastomosis of aorta to aorta NEC			1		

L19.4	Replacement of aneurysmal segment of infrarenal abdominal aorta by anastomosis of aorta to aorta NEC			1		
L19.5	Replacement of aneurysmal segment of abdominal aorta by anastomosis of aorta to aorta NEC			1		
L19.6	Replacement of aneurysmal bifurcation of aorta by anastomosis of aorta to iliac artery NEC			1		
L19.8	Other specified other replacement of aneurysmal segment of aorta			1		
L19.9	Unspecified other replacement of aneurysmal segment of aorta			1		
L20	Other emergency bypass of segment of aorta			1		
L20.3	Emergency bypass of segment of suprarenal abdominal aorta by anastomosis of aorta to aorta NEC			1		
L20.4	Emergency bypass of segment of infrarenal abdominal aorta by anastomosis of aorta to aorta NEC			1		
L20.5	Emergency bypass of segment of abdominal aorta by anastomosis of aorta to aorta NEC			1		
L20.6	Emergency bypass of bifurcation of aorta by anastomosis of aorta to iliac artery NEC			1		
L22.2	Revision of prosthesis of bifurcation of aorta			1		
L22.3	Revision of prosthesis of abdominal aorta NEC			1		
L22.4	Removal of prosthesis from aorta			1		
L23.5	Revision of plastic repair of aorta			1		
L25.4	Operations on aneurysm of aorta NEC			1		
L27	Transluminal insertion of stent graft for aneurysmal segment of aorta			1		
L27.1	Endovascular insertion of stent graft for infrarenal abdominal aortic aneurysm			1		
L27.2	Endovascular insertion of stent graft for suprarenal aortic aneurysm			1		
L27.5	Endovascular insertion of stent graft for aortic aneurysm of bifurcation NEC			1		
L27.6	Endovascular insertion of stent graft for aorto-uniiliac aneurysm			1		
L27.8	Other specified transluminal insertion of stent graft for aneurysmal segment of aorta			1		
L27.9	Unspecified transluminal insertion of stent graft for aneurysmal segment of aorta			1		
L28	Transluminal operations on aneurysmal segment of aorta			1		
L28.1	Endovascular insertion of stent for infrarenal abdominal aortic aneurysm			1		
L28.2	Endovascular insertion of stent for suprarenal aortic aneurysm			1		
L28.5	Endovascular insertion of stent for aortic aneurysm of bifurcation NEC			1		
L28.6	Endovascular insertion of stent for aorto-uniiliac aneurysm			1		
L50	Other emergency bypass of iliac artery			1		
L50.1	Emergency bypass of common iliac artery by anastomosis of aorta to common iliac artery NEC			1		
L50.2	Emergency bypass of iliac artery by anastomosis of aorta to external iliac artery NEC			1		
L50.3	Emergency bypass of artery of leg by anastomosis of aorta to common femoral artery NEC			1		

L50.4	Emergency bypass of artery of leg by anastomosis of aorta to deep femoral artery NEC			1		
L50.5	Emergency bypass of iliac artery by anastomosis of iliac artery to iliac artery NEC			1		
L50.6	Emergency bypass of artery of leg by anastomosis of iliac artery to femoral artery NEC			1		
L50.8	Other specified other emergency bypass of iliac artery			1		
L50.9	Unspecified other emergency bypass of iliac artery			1		
L51	Other bypass of iliac artery			1		
L51.1	Bypass of common iliac artery by anastomosis of aorta to common iliac artery NEC			1		
L51.2	Bypass of iliac artery by anastomosis of aorta to external iliac artery NEC			1		
L51.3	Bypass of artery of leg by anastomosis of aorta to common femoral artery NEC			1		
L51.4	Bypass of artery of leg by anastomosis of aorta to deep femoral artery NEC			1		
L51.5	Bypass of iliac artery by anastomosis of iliac artery to iliac artery NEC			1		
L51.6	Bypass of artery of leg by anastomosis of iliac artery to femoral artery NEC			1		
L51.8	Other specified other bypass of iliac artery			1		
L51.9	Unspecified other bypass of iliac artery			1		
L52	Reconstruction of iliac artery			1		
L52.1	Endarterectomy of iliac artery and patch repair of iliac artery			1		
L52.2	Endarterectomy of iliac artery NEC			1		
L52.8	Other specified reconstruction of iliac artery			1		
L52.9	Unspecified reconstruction of iliac artery			1		
L53	Other open operations on iliac artery			1		
L53.1	Repair of iliac artery NEC			1		
L53.2	Open embolectomy of iliac artery			1		
L54.1	Percutaneous transluminal angioplasty of iliac artery			1		
L54.2	Percutaneous transluminal embolectomy of iliac artery			1		
L54.4	Percutaneous transluminal insertion of stent into iliac artery			1		
L54.8	Other specified transluminal operations on iliac artery			1		
L54.9	Unspecified transluminal operations on iliac artery			1		
L58	Other emergency bypass of femoral artery			1		
L58.1	Emergency bypass of femoral artery by anastomosis of femoral artery to femoral artery NEC			1		
L58.2	Emergency bypass of femoral artery by anastomosis of femoral artery to popliteal artery using prosthesis NEC			1		
L58.3	Emergency bypass of femoral artery by anastomosis of femoral artery to popliteal artery using vein graft NEC			1		

L58.4	Emergency bypass of femoral artery by anastomosis of femoral artery to tibial artery using prosthesis NEC			1		
L58.5	Emergency bypass of femoral artery by anastomosis of femoral artery to tibial artery using vein graft NEC			1		
L58.6	Emergency bypass of femoral artery by anastomosis of femoral artery to peroneal artery using prosthesis NEC			1		
L58.7	Emergency bypass of femoral artery by anastomosis of femoral artery to peroneal artery using vein graft NEC			1		
L58.8	Other specified other emergency bypass of femoral artery			1		
L58.9	Unspecified other emergency bypass of femoral artery			1		
L59	Other bypass of femoral artery			1		
L59.1	Bypass of femoral artery by anastomosis of femoral artery to femoral artery NEC			1		
L59.2	Bypass of femoral artery by anastomosis of femoral artery to popliteal artery using prosthesis NEC			1		
L59.3	Bypass of femoral artery by anastomosis of femoral artery to popliteal artery using vein graft NEC			1		
L59.4	Bypass of femoral artery by anastomosis of femoral artery to tibial artery using prosthesis NEC			1		
L59.5	Bypass of femoral artery by anastomosis of femoral artery to tibial artery using vein graft NEC			1		
L59.6	Bypass of femoral artery by anastomosis of femoral artery to peroneal artery using prosthesis NEC			1		
L59.7	Bypass of femoral artery by anastomosis of femoral artery to peroneal artery using vein graft NEC			1		
L59.8	Other specified other bypass of femoral artery			1		
L59.9	Unspecified other bypass of femoral artery			1		
L60	Reconstruction of femoral artery			1		
L60.1	Endarterectomy of femoral artery and patch repair of femoral artery			1		
L60.2	Endarterectomy of femoral artery NEC			1		
L60.3	Profundoplasty of femoral artery and patch repair of deep femoral artery			1		
L60.4	Profundoplasty of femoral artery NEC			1		
L60.8	Other specified reconstruction of femoral artery			1		
L60.9	Unspecified reconstruction of femoral artery			1		
L62	Other open operations on femoral artery			1		
L62.1	Repair of femoral artery NEC			1		
L62.2	Open embolectomy of femoral artery			1		
L62.8	Other specified other open operations on femoral artery			1		
L62.9	Unspecified other open operations on femoral artery			1		
L63.1	Percutaneous transluminal angioplasty of femoral artery			1		
L63.2	Percutaneous transluminal embolectomy of femoral artery			1		
L63.3	Percutaneous transluminal embolisation of femoral artery			1		

L63.5	Percutaneous transluminal insertion of stent into femoral artery			1		
L65	Revision of reconstruction of artery			1		
L65.1	Revision of reconstruction involving aorta			1		
L65.2	Revision of reconstruction involving iliac artery			1		
L65.3	Revision of reconstruction involving femoral artery			1		
K48.3	Open angioplasty of coronary artery	1				
L97.2	Peroperative angioplasty	1				
L29.4	Endarterectomy of carotid artery and patch repair of carotid artery		1			
L29.5	Endarterectomy of carotid artery NEC		1			
L29.6	High-flow interposition extracranial to intracranial bypass from external carotid artery to middle cerebral artery		1			
L29.7	Bypass of carotid artery by anastomosis of superficial temporal artery to middle cerebral artery		1			
L31.1	Percutaneous transluminal angioplasty of carotid artery		1			
L31.4	Percutaneous transluminal insertion of stent into carotid artery		1			
L35.3	Percutaneous transluminal insertion of stent into cerebral artery		1			
L37.2	Endarterectomy of vertebral artery		1			

Target organ damage Read codes

Chronic kidney disease

medcode	readcode	readterm
12479	1Z13.00	Chronic kidney disease stage 4
12566	1Z12.00	Chronic kidney disease stage 3
12585	1Z14.00	Chronic kidney disease stage 5
12586	1Z11.00	Chronic kidney disease stage 2
12860	9hE0.00	Except chronic kidney disease qual indic: Patient unsuitable
19473	66i..00	Chronic kidney disease monitoring
29013	1Z10.00	Chronic kidney disease stage 1
30735	6AA..00	Chronic kidney disease annual review
30739	9Ot0.00	Chronic kidney disease monitoring first letter
40100	9hE1.00	Exc chronic kidney disease quality indicators: Inform dissen
46626	9hE..00	Exception reporting: chronic kidney disease quality indicato
69679	9Ot4.00	Chronic kidney disease monitoring telephone invite
71271	9Ot..00	Chronic kidney disease monitoring administration
72962	9Ot1.00	Chronic kidney disease monitoring second letter
72964	9Ot2.00	Chronic kidney disease monitoring third letter
88494	9Ot3.00	Chronic kidney disease monitoring verbal invite
89332	9Ot5.00	Predicted stage chronic kidney disease
94789	1Z17.00	Chronic kidney disease stage 1 with proteinuria
94793	1Z1B.00	Chronic kidney disease stage 3 with proteinuria
94965	1Z15.00	Chronic kidney disease stage 3A
95121	1Z1A.00	Chronic kidney disease stage 2 without proteinuria
95122	1Z1H.00	Chronic kidney disease stage 4 with proteinuria
95123	1Z1C.00	Chronic kidney disease stage 3 without proteinuria
95145	1Z1B.11	CKD stage 3 with proteinuria
95146	1Z19.00	Chronic kidney disease stage 2 with proteinuria
95175	1Z1E.00	Chronic kidney disease stage 3A without proteinuria
95176	1Z1E.11	CKD stage 3A without proteinuria
95177	1Z1G.00	Chronic kidney disease stage 3B without proteinuria
95178	1Z1F.00	Chronic kidney disease stage 3B with proteinuria
95179	1Z16.00	Chronic kidney disease stage 3B
95180	1Z1F.11	CKD stage 3B with proteinuria
95188	1Z1C.11	CKD stage 3 without proteinuria
95405	1Z1L.00	Chronic kidney disease stage 5 without proteinuria
95406	1Z1J.00	Chronic kidney disease stage 4 without proteinuria
95408	1Z1D.00	Chronic kidney disease stage 3A with proteinuria
95422	9Ni9.00	Did not attend chronic kidney disease monitoring clinic
95508	1Z1K.00	Chronic kidney disease stage 5 with proteinuria
95571	1Z1D.11	CKD stage 3A with proteinuria
95572	1Z18.00	Chronic kidney disease stage 1 without proteinuria
97587	1Z1J.11	CKD stage 4 without proteinuria
97683	1Z1L.11	CKD stage 5 without proteinuria
97978	1Z1A.11	CKD stage 2 without proteinuria
97979	1Z19.11	CKD stage 2 with proteinuria
97980	1Z17.11	CKD stage 1 with proteinuria
99160	1Z1K.11	CKD stage 5 with proteinuria

99312	1Z1H.11	CKD stage 4 with proteinuria
99813	14OY.00	At risk of chronic kidney disease
100633	1Z1G.11	CKD stage 3B without proteinuria
104619	K053.00	Chronic kidney disease stage 3
104963	K054.00	Chronic kidney disease stage 4
104981	K05..13	Chronic kidney disease
105009	68D1.11	Chronic kidney disease screening
105151	K055.00	Chronic kidney disease stage 5
105383	K052.00	Chronic kidney disease stage 2
105392	K051.00	Chronic kidney disease stage 1
108766	661M200	Chronic kidney disease self-management plan agreed
109657	1Z1Y.00	CKD with GFR category G3b & albuminuria category A2
109804	1Z1T.00	CKD with GFR category G3a & albuminuria category A1
109805	1Z1V.00	CKD with GFR category G3a & albuminuria category A2
109904	1Z1b.00	CKD with GFR category G4 & albuminuria category A2
109905	1Z1W.00	CKD with GFR category G3a & albuminuria category A3
109963	1Z1X.00	CKD with GFR category G3b & albuminuria category A1
109980	1Z1a.00	CKD with GFR category G4 & albuminuria category A1
109981	1Z1e.00	CKD with GFR category G5 & albuminuria category A2
109990	1Z1Z.00	CKD with GFR category G3b & albuminuria category A3
110003	1Z1N.00	CKD with GFR category G1 & albuminuria category A2
110033	1Z1M.00	CKD with GFR category G1 & albuminuria category A1
110108	1Z1R.00	CKD with GFR category G2 & albuminuria category A2
110133	1Z1d.00	CKD with GFR category G5 & albuminuria category A1
110251	1Z1S.00	CKD with GFR category G2 & albuminuria category A3
110269	1Z1Q.00	CKD with GFR category G2 & albuminuria category A1
110467	1Z1f.00	CKD with GFR category G5 & albuminuria category A3
110484	1Z1P.00	CKD with GFR category G1 & albuminuria category A3
110626	1Z1c.00	CKD with GFR category G4 & albuminuria category A3
111022	1Z18.11	CKD stage 1 without proteinuria

Proteinuria

medcode	readcode	readterm
1802	4678	Proteinuria
13600	4677	Urine protein test = ++++
13611	4675	Urine protein test = ++
13613	46N2.00	Urine protein abnormal
13621	4676	Urine protein test = +++
14901	K136.00	Benign postural proteinuria
16465	K190X00	Persistent proteinuria, unspecified
26054	C10FL00	Type 2 diabetes mellitus with persistent proteinuria
30323	C10EK00	Type 1 diabetes mellitus with persistent proteinuria
36243	K136.11	Orthostatic proteinuria
43611	K0A4.00	Isolated proteinuria with specified morphological lesion
44804	K0A4100	Isolatd proteinur/specifd morphlgcl les foc+seg glom lesn
59992	K0A4W00	Isolated proteinuria, with unspecified morpholog changes
60796	C10FL11	Type II diabetes mellitus with persistent proteinuria
61470	66Al.00	Diabetic monitoring - higher risk albumin excretion
61811	K0A4500	Isoltd prteinur+specfd morph les df mesangiocap glomnephr
64030	Kyu5G00	[X]Persistent proteinuria, unspecified
66475	66Ak.00	Diabetic monitoring - lower risk albumin excretion
66613	K0A4300	Isoltd prteinur/spcfd morph lesn df mesngl prolf glomneph
71964	K0A4200	Isolatd proteinur/specfd morphlgcl les df membrn glomneph
94789	1Z17.00	Chronic kidney disease stage 1 with proteinuria
94793	1Z1B.00	Chronic kidney disease stage 3 with proteinuria
95122	1Z1H.00	Chronic kidney disease stage 4 with proteinuria
95145	1Z1B.11	CKD stage 3 with proteinuria
95146	1Z19.00	Chronic kidney disease stage 2 with proteinuria
95178	1Z1F.00	Chronic kidney disease stage 3B with proteinuria
95180	1Z1F.11	CKD stage 3B with proteinuria
95408	1Z1D.00	Chronic kidney disease stage 3A with proteinuria
95508	1Z1K.00	Chronic kidney disease stage 5 with proteinuria
95571	1Z1D.11	CKD stage 3A with proteinuria
97979	1Z19.11	CKD stage 2 with proteinuria
97980	1Z17.11	CKD stage 1 with proteinuria
99160	1Z1K.11	CKD stage 5 with proteinuria
99312	1Z1H.11	CKD stage 4 with proteinuria
101572	K0A4X00	Isolated proteinuria, with oth specif morpholog changes
105302	K08yA00	Proteinuric diabetic nephropathy
109657	1Z1Y.00	CKD with GFR category G3b & albuminuria category A2
109805	1Z1V.00	CKD with GFR category G3a & albuminuria category A2
109904	1Z1b.00	CKD with GFR category G4 & albuminuria category A2
109905	1Z1W.00	CKD with GFR category G3a & albuminuria category A3
109981	1Z1e.00	CKD with GFR category G5 & albuminuria category A2
109990	1Z1Z.00	CKD with GFR category G3b & albuminuria category A3
110003	1Z1N.00	CKD with GFR category G1 & albuminuria category A2
110108	1Z1R.00	CKD with GFR category G2 & albuminuria category A2
110251	1Z1S.00	CKD with GFR category G2 & albuminuria category A3
110467	1Z1f.00	CKD with GFR category G5 & albuminuria category A3
110484	1Z1P.00	CKD with GFR category G1 & albuminuria category A3
110626	1Z1c.00	CKD with GFR category G4 & albuminuria category A3

Hypertensive Retinopathy

Medcode	Read term	Read code
6702	Hypertensive retinopathy	F421300

Left ventricular hypertrophy

medcode	readcode	readterm
562	G5y3411	Left ventricular hypertrophy
2724	G5y3400	Ventricular hypertrophy
6319	324..00	ECG:left ventricle hypertrophy
13857	3242	ECG: shows LVH
23142	324Z.00	ECG: LVH NOS
61124	G5y3500	Cardiac hypertrophy NOS
64242	2492	O/E - apex beat displaced -LVH

ISAC protocol

PROTOCOL INFORMATION REQUIRED

The following sections below **must** be included in the CPRD ISAC research protocol. Please refer to the guidance on '***Contents of CPRD ISAC Research Protocols***' (www.cprd.com/isac) for more information on how to complete the sections below. Pages should be numbered. All abbreviations must be defined on first use.

**Applicants must complete all sections listed below**<br>**Sections which do not apply should be completed as '*Not Applicable*'**
**A. Study Title**§<br>§*Please note: This information will be published on CPRD's website as part of its transparency policy*<br><br>Eligibility for blood pressure lowering treatment and subsequent cardiovascular disease burden under four different treatment approaches: a cohort study
**B. Lay Summary (Max. 200 words)**§<br>§*Please note: This information will be published on CPRD's website as part of its transparency policy*<br><br>Blood pressure lowering drugs are extremely effective in reducing heart attacks and strokes. In the UK, only patients whose blood pressures are above a certain threshold are considered for treatment. However, recent research studies have shown that even patients with blood pressures below that threshold might benefit from treatment and therefore the current approach may not be the best way to save lives.<br><br>Other possible approaches to blood pressure lowering treatment include treating based on a patient's age and sex (e.g. treat men >55 and women >65 years), or treating based on a patient's overall cardiovascular disease risk, which takes into account other factors like diabetes, smoking and older age. This approach is used for deciding who should be offered statins.<br><br>This research aims to provide information to help decide which of four proposed strategies for recommending blood pressure treatment is best for population health. In particular, we will analyse primary care and linked hospital records from CPRD to first work out the number of patients who would be eligible for treatment under each suggested approach, and second, to look at which strategy prioritises treatment to patients who go on to experience heart attacks and strokes.
**C. Technical Summary (Max. 200 words)**§<br>§*Please note: This information will be published on CPRD's website as part of its transparency policy*<br>The current approach to blood pressure treatment in the UK relies predominantly on blood pressure thresholds. This ignores evidence showing that treatment lowers cardiovascular disease (CVD) risk at all blood pressures, with no threshold. One alternative treatment approach would be to use a threshold of overall CVD risk, a strategy that is now accepted for lipid lowering drugs. Another suggested approach is to use age and sex to determine treatment.<br><br>To help decide on the optimal strategy for population health, there are two key considerations: the number of patients who would be eligible for treatment under each strategy, and whether the strategy prioritises treatment to patients

with the highest future burden of CVD. This protocol describes a cohort study to investigate eligibility for blood pressure lowering treatment under four different approaches (i) current NICE guideline, (ii) blood pressure alone (to reflect evidence that some GPs are ignoring the NICE guideline), (iii) cardiovascular risk, and (iv) age/sex. Beginning in 2011, the cohort will be split according to treatment eligibility under each strategy, and followed up for their first cardiovascular disease event. Rates and proportions of events, according to treatment eligibility, will be reported.

D. Objectives, Specific Aims and Rationale

The overall objective is to describe and compare eligibility for blood pressure lowering treatment under four different approaches, and relate this to subsequent cardiovascular disease burden among eligible and ineligible patients.

Specific aims are:
Among a cohort of CPRD patients:

1. To use baseline data to determine patients' treatment eligibility based on four different approaches to blood pressure lowering treatment: (i) blood pressure alone, (ii) current NICE guidelines, (iii) QRISK2 score, and (iv) age and sex.
2. To describe the rate and proportion of cardiovascular disease events that occur during follow-up in patients eligible and ineligible for treatment under each of the four approaches.
3. To describe the rate and proportion of cardiovascular disease events that occur during follow-up in patients grouped according to their blood pressure, overall cardiovascular disease risk, and age and sex.

Rationale
To help decide which blood pressure treatment strategy is optimal for population health, this study will address two key considerations: the number of patients who would be eligible for treatment under each strategy, and whether the strategy prioritises treatment to patients with the highest future burden of CVD. In addition to the focus on treatment eligibility, this study will also investigate the ability of *different levels* of blood pressure alone, *different levels* of overall cardiovascular disease risk, and *different age groups* to discriminate between patients who will and won't have cardiovascular disease events in the future.

E. Study Background

The current approach to blood pressure treatment
High blood pressure is the most common, modifiable risk factor for cardiovascular disease (CVD).[1] Randomised trials have shown that blood pressure reductions, whether by diet, lifestyle or drug therapy, reduce the risk of CVD.[2, 3] Guidance from the National Institute for Health and Care Excellence (NICE) recommends that clinicians consider pharmacological intervention for primary prevention of CVD when clinic blood pressure is ≥140/90 mm Hg, confirmed by ambulatory monitoring ('hypertension') **and** ten year cardiovascular disease risk is at least 20%, or if blood pressure is >160/100mmHg.[4]

However, the use of a blood pressure threshold to determine eligibility for treatment may be suboptimal for two reasons. Firstly, there is strong evidence for a continuous, log-linear association between blood pressure and CVD risk between 110/70 and 170/105 mm Hg with no evidence of a threshold.[5, 6] Furthermore, meta-analyses of randomised and non-randomised studies showed that treating blood pressures across this whole range reduces CVD risk.[2, 7] Secondly, the use of a blood pressure threshold to determine treatment ignores overall or 'absolute' CVD risk, which is strongly influenced by other CVD risk factors. For example, a patient with a systolic pressure of 160 mm Hg could have a five year absolute risk as low as 2% if no other risk factors are present, and would qualify for treatment under the current approach, but a patient with a systolic pressure of 130 mm Hg could have a risk as high as 35% and would not qualify.[8]

Using absolute CVD risk to guide treatment
Since the aim of blood pressure lowering treatment is to reduce CVD risk, a rational alternative strategy would be to treat those with highest risk, rather than only those with 'hypertension'. Such a strategy would involve using a

validated CVD risk score (such as QRISK2,[9] or QRISK3,[10] calculated based on blood pressure and other risk factors) to determine treatment. CVD prevention based on absolute CVD risk rather than on a single risk factor threshold is now accepted for lipid lowering drugs, but has not been widely accepted for blood pressure.

Guidelines in New Zealand already recommend an absolute CVD risk-based approach to blood pressure lowering treatment. Additionally there is a growing body of international evidence that a risk-based approach may be superior to a blood pressure threshold-based approach in terms of CVD prevention.[11-14] Studies have also demonstrated that a risk-based approach is cost effective; projected savings in an Australian study were over $5.4 billion AUD.[13, 15] This could equate to similar savings in the UK in terms of NHS cost and could allow redistribution of healthcare resources towards populations with greatest need.

Other approaches to guide treatment

Prior to 2011, NICE guidance used simple blood pressure thresholds alone to determine treatment. The 2011 guideline still categorises patients with blood pressures below 140/90mm Hg as ineligible for treatment. However, patients with blood pressures between 140/90 and 160/100mm Hg were made eligible for treatment only if they have an absolute risk of CVD of ≥20%, or they have diabetes, kidney disease, or target organ damage. Despite this updated guideline, there is some evidence to suggest that GPs ignore the component of risk and continue to use simple cut offs to guide treatment decisions.[16]

Another suggested approach to blood pressure lowering treatment is to base decisions on age and sex (e.g. all men over 55, all women over 65[17]). This approach reflects the high underlying risk of CVD amongst older age groups, but is much simpler than using QRISK2 as only age and sex are required to make a judgement about treatment.

Determining the best approach

Two key considerations in determining the optimal approach to blood pressure lowering treatment are (i) the number of patients eligible for treatment under each approach, and (ii) which approach prioritises treatment to the group of patients who are most likely to experience a CVD event. Therefore, the objective of this study is to describe and compare eligibility for blood pressure lowering treatment under four different approaches, and relate this to subsequent cardiovascular disease burden among eligible and ineligible patients.

F. Study Type

Descriptive

G. Study Design

An open cohort of patients without cardiovascular disease event will be created. Patient records will be reviewed to establish their blood pressure, age, sex, other cardiovascular risk factors and predicted absolute CVD risk. Patients in the cohort will be categorised as eligible or ineligible for treatment under four different approaches: (i) blood pressure alone, (ii) current NICE guidelines for blood pressure treatment, (iii) QRISK2 absolute disease risk, and (iv) age and sex. Patients will then be followed until the earliest of a CVD event (the outcome), death, or end of CPRD follow-up.

A cohort has been chosen in order to capture the distribution of blood pressure and overall cardiovascular disease risk at baseline, and to use these categorisations to observe the rate of CVD events during follow-up.

Applicants must complete all sections listed below

Sections which do not apply should be completed as '*Not Applicable*'

H. Feasibility counts

In a previous study examining a cohort of patients in the CPRD without existing cardiovascular disease, 1.25 million patients were included and one fifth of these were using blood pressure lowering treatment. Five year follow up yielded over 80,000 CVD diagnoses.[18] Pilot data for the present study, later than the previous study and using fewer linked sources, estimated a rate of 6.2 CVD events per 1000 person years, and a conservative estimate of 23,000 events (based on a more restricted set of acute CVD outcomes). Therefore, the feasibility counts have been calculated based on this very conservative event rate.

Feasibility counts have been carried out to estimate the likely number of events over five year follow up. These are shown in the table below. Our pilot data show that across all strategies, the minimum proportion of eligible patients (i.e. the most prudent treatment strategy) is likely to be 20%. Predicted confidence intervals have been calculated for the most prudent treatment strategy, and within subgroups of that treatment strategy. This includes the secondary analyses investigating blood pressure lowering treatment use, age 80+ vs <80, and statin use.

	Estimated N patients in cohort	Expected N events during follow-up$	Expected % events in subgroup	Expected 95% confidence interval
Total patients without CVD at cohort entry	1,250,000	23,250	-	
Minimum number eligible under the most prudent treatment strategy	250,000	4,650	20.0	(18.9-21.1)
**Stratification for secondary analyses - minimum numbers expected under most prudent treatment strategy (N=250,000):**				
On blood pressure lowering treatment*	50,000	930	4.0	(2.7-5.3)
Not on blood pressure lowering treatment	200,000	3,720	16.0	(14.8-17.2)
Age 80+**	12,500	233	1.0	(-0.3-2.3)
Age <80	237,500	4,418	19.0	(17.8-20.2)
Statin users***	15,000	279	0.6	(-0.1-2.5)
Statin non-users	242,500	4,511	19.4	(17.6-20.0)

$Assuming patients are followed up for an average of 3 years, and the average rate of CVD in each group is 6.2 per 1000 person years (from pilot data)

*Assume 20% of patients are using blood pressure lowering treatment[18]

**Assume 5% of adult patients are 80 of over, (ONS Overview of the UK population, 2017)

Applicants must complete all sections listed below

Sections which do not apply should be completed as '*Not Applicable'*

***Assume 40% of patients are eligible for a statin (based on pilot data) and uptake is 15%, based on published information about statin prescriptions, cessation and health care check uptake in the UK[19-21]

I. Sample size considerations

See section H.

J. Data Linkage Required (if applicable):§

§*Please note that the data linkage/s requested in research protocols will be published by the CPRD as part of its transparency policy*

Data linkage will be required for:

- Hospital episode statistics (HES): this will improve identification of CVD events by recording those that required hospital admission, but were not identified in primary care records.
- Townsend Score data: Townsend data will give a measure of deprivation at the patient level. This will be used in the calculation of QRISK2.
- Pregnancy register to identify and exclude pregnant women.

K. Study population

The study population will be comprised of patients who are over the age of 18, who do not have a diagnosis of cardiovascular disease, who are eligible for HES and IMD linkage, and with at least 12 months of follow-up in CPRD data.

- The start of follow-up will be defined as the latest of: current registration date, up to standard date, first blood pressure measurement, or 1st January 2011.
- The end of follow-up will be the earliest of: first cardiovascular disease record, transfer out date, last collection date, or the end of the study period six months prior to the latest available data cut at time of analysis.

The overall start and end of follow-up will also be truncated by the linkage coverage dates.

Exclusions:

Pregnant individuals will be excluded, as blood pressure lowering treatment is contraindicated during pregnancy. Patients with existing cardiovascular disease will be excluded: this includes myocardial infarction, stroke, transient ischaemic attack, non-stroke cerebrovascular disease, stable and unstable angina, previous PCI or CABG, heart failure, peripheral arterial disease including intermittent claudication and abdominal aortic aneurysm, procedures indicating repair of peripheral arterial disease and abdominal aortic aneurysm, and codes indicating other CHD.

L. Selection of comparison group(s) or controls

Applicants must complete all sections listed below

Sections which do not apply should be completed as '*Not Applicable*'

There is no control or comparison group. This study will compare the numbers of patients eligible at baseline for treatment under four different approaches, and the subsequent rate of cardiovascular disease, and number of cardiovascular disease events in eligible and ineligible patients.

M. Exposures, Health Outcomes§ and Covariates

§*Please note: Summary information on health outcomes (as included on the ISAC application form above)will be published on CPRD's website as part of its transparency policy*

Exposures

Aims 1 & 2

Four different approaches to blood pressure lowering treatment will be investigated:

(i) Blood pressure alone

Patients with blood pressures of ≥140/90mm Hg will be categorised as eligible for treatment. Patients with isolated systolic blood pressure ≥140mm Hg or isolated diastolic blood pressure >90 MM Hg will be categorised as eligible for treatment. Systolic and diastolic blood pressures will be taken from the additional clinical details file.

(ii) NICE guidance 2011

- Patients with blood pressures ≥160/100mm Hg will be categorised as eligible for treatment.
- Patients with blood pressures between 140/90 and 160/100mm Hg will be categorised as eligible for treatment if they have target organ damage*, renal disease, diabetes or a QRISK2 score ≥20% (code lists in appendices).
- Patients with blood pressures below 140/90mm Hg will be categorised as ineligible for treatment.

(*Target organ damage will include hypertensive retinopathy, heart failure, proteinuria (code lists in appendices))
Patients with diabetes will be identified using the same code lists and approach as Mathur et al.[22]

(iii) QRISK2

QRISK2 scores will be calculated based on the approach taken by Bhaskaran, Gadd et al (ISAC 17_008). QRISK2 scores will be calculated using bulk processing software provided by ClinRisk, using the most recent measures available. Variables in QRISK2 include age, sex, ethnicity, blood pressure, deprivation score from linked data, diabetes, family history of coronary heart disease, atrial fibrillation, chronic kidney disease stage 4 or 5, cholesterol/HDL ratio, rheumatoid arthritis, use of blood pressure lowering drugs, BMI (using weight and height records), smoking status (using smoking-related Read codes and structured data on smoking). Time variant variables such as BMI and smoking status will be recorded at cohort entry using the most recent record within one year before the start of follow-up, where available.

Patients with a QRISK2 score of ≥10% (reflecting the same 10% threshold for statin eligibility in primary prevention) will be categorised as eligible for treatment. The study will also explore thresholds of 15% and 20% QRISK2 scores.

(iv) Age and sex

Men aged ≥55 and women aged ≥65 will be categorised as eligible for treatment. Additional cut offs will also be explored (men aged ≥50, women aged ≥60, and men aged ≥60, women aged ≥70).

Aim 3

Blood pressure, QRISK2, and categories of age will be split into standard groupings:

- Systolic blood pressure; categorised at cohort entry (<119, 120-139, 140-159, 160-179, 180+mm Hg)

Applicants must complete all sections listed below

Sections which do not apply should be completed as '*Not Applicable'*

- Diastolic blood pressure; categorised at cohort entry (<80, 80-89, 90-99, 100-109, 110+mm Hg)
- QRISK2 will be categorised into bands of percentage risk (<10%, 10-19%, 20-29%, 30%+)
- Year of birth; age categorised into 10 year bands at cohort entry (<30, 30-39, 40-49, 50-59, 60-69, 70-79, 80-89, 90+)

Outcomes

Outcomes of interest in aims 2 and 3 are CVD. CVD will be defined as any record of coronary heart disease (myocardial infarction, angina, revascularisation procedures, coronary heart disease not otherwise specified), cerebrovascular disease (stroke, transient ischaemic attack, non-stroke cerebrovascular disease), peripheral arterial disease (including abdominal aortic aneurism, intermittent claudication and their treatment) and heart failure. Cardiovascular outcomes will be ascertained through CPRD and HES linkage. Medical and ICD code lists for cardiovascular disease are provided in appendices.

N. Data/ Statistical Analysis

Statistical analysis will be carried out using STATA MP version 14.2 statistical software.

Aim 1 – eligibility under four different approaches to blood pressure lowering treatment

Under each treatment approach, eligibility will be determined at cohort entry (latest of 1st January 2011, first blood pressure measure, UTS date). The number of patients eligible under each strategy will be calculated and eligibility will be compared between approaches using cross tabulations. Eligible and ineligible patients will be described according to their cardiovascular disease risk factors (variables included in QRISK2).

For the approach using blood pressure thresholds alone (strategy i) and the approach based on NICE guidelines (strategy ii), patients will be additionally categorised according to their use of treatment at cohort entry. This will allow stratification according to control of blood pressure by treatment:

- Controlled blood pressure: on treatment and BP <140/90mm Hg
- Uncontrolled blood pressure: on treatment and BP ≥140/90mm Hg
- Untreated high blood pressure: no treatment and BP ≥140/90mm Hg

Aim 2 – rate and proportion of CVD events occurring during follow-up, by treatment eligibility

Patients in the cohort will be followed up for the outcome of their first cardiovascular disease event. The total number of events and overall rate of cardiovascular disease will be calculated.

For each treatment approach, the cohort will be split according to treatment eligibility (eligible vs. ineligible) at cohort entry and eligibility will be updated during follow-up when new measures become available. QRISK2 scores will be updated each time a new measure becomes available or every year (to reflect change in age), whichever is sooner. Men and women who reach the age cut offs for treatment in strategy iv will be updated as eligible.

The number of CVD events throughout follow-up will be calculated. The incidence rate of CVD in eligible and ineligible patients will be calculated using the number of events and person-years. The proportion of all CVD events that occur in patients eligible for treatment at the time of their first CVD diagnosis (vs not eligible for treatment at their first CVD diagnosis) will be described and compared across treatment strategies (see **shell table 1** below). An approach using proportions is appropriate for these follow-up data because the whole cohort is followed up for the same period, no matter how the cohort is split.

Applicants must complete all sections listed below

Sections which do not apply should be completed as '*Not Applicable'*

Shell table 1. Rate of CVD and proportion of CVD events occurring among eligible patients for each strategy.

**Strategy**	**Rate of CVD/1000 person yrs**		**% CVD events during follow-up that occur among patients eligible for treatment**
	**Among those eligible for treatment**	**Among those not eligible for treatment**	
Blood pressure only			
NICE guideline			
QRISK2 10% cut off			
Age and sex:<br>Age 55 for men<br>Age 65 for women			

For the QRISK2-based approach to treatment, analyses will initially be performed for a 10% cut-off. Additional analyses will investigate a 15% and 20% cut-off. For the age/sex-based approach to treatment, analyses will initially be performed for an age 55 cut off for men and age 65 cut off for women. Additional analyses will investigate cut-offs of 50 and 60 for men, and 60 and 70 for women.

Aim 3 – rate and proportion of CVD events occurring during follow-up, by groups of blood pressure, risk, and age/sex

For each treatment approach, the cohort will be split into groups at cohort entry, as described in section M, Aim 3. Patients will be able to switch between groups during follow-up to reflect changes in their risk factor profile. In each group, the rate and proportion of CVD events will be calculated and compared across treatment strategies. This analysis will allow us to complete the **shell table 2**.

Shell table 2. Rate of CVD and proportion of events occurring in each category.

**Strategy**	**Category**	**Rate of CVD/1000 person yrs**	**% of all follow-up events that occur in group**
**Systolic blood pressure, mm Hg**	<119		
	120-139		
	140-159		
	160-179		
	180+		
**Diastolic blood pressure, mm Hg**	<80		
	80-89		
	90-99		
	100-109		
	110+		
**QRISK2**	<10%		
	10-19%		
	20-29%		

	30%+		
**Men: Age, years**	<30		
	30-39		
	40-49		
	50-59		
	60-69		
	70-79		
	80-89		
	90+		
**Women: Age, years**	<30		
	30-39		
	40-49		
	50-59		
	60-69		
	70-79		
	80-89		
	90+		

Aims 1-3 secondary analyses

Investigating use of blood pressure lowering treatment

As this cohort takes real life patients, some will use blood pressure lowering treatment at cohort entry or will start treatment during follow-up. This will reduce their risk of CVD during follow-up, and will affect the burden of CVD, particularly among the group eligible for treatment under the approach used by NICE.

Therefore, in order to maximise comparability of the burden according to each treatment strategy, for each of the four treatment strategies, patients will be stratified according to their use of blood pressure lowering at cohort entry (for Aim 1) and at the time of event (Aims 2 and 3). The burden of CVD can then be compared across strategies among treated and untreated patients. The rate and burden of CVD will also be assessed according to controlled blood pressure, uncontrolled blood pressure and untreated blood pressure.

Investigating the impact of age

The current guidance for blood pressure lowering is slightly different in patients over 80 and advises more caution, given their likelihood of falls. Therefore, eligibility at cohort entry will be stratified by age <80 vs 80+ years for each strategy. The rate and proportion of CVD events will also be stratified by age <80 vs 80+ at the time of the event.

Investigating statin use

Many patients in the cohort are likely to use statins. Where there is overlap between statin use and eligibility for blood pressure lowering, the burden of disease will be lower due to statin use. To investigate the impact of statin use on the burden, we will stratify by statin use in a sensitivity analysis. For each of the four treatment strategies, patients will be stratified according to their use of statins at cohort entry (for Aim 1) and at the time of event (Aims 2 and 3). The burden of CVD can then be compared across strategies among treated and untreated patients.

Investigating different types of CVD

To further understand the burden of CVD according to each treatment strategy, CVD will be split into two categories in a sensitivity analysis:

- MI and stroke
- Other CVD

**Applicants must complete all sections listed below**<br>**Sections which do not apply should be completed as '*Not Applicable*'**
**O. Plan for addressing confounding**<br>This is a descriptive study examining numbers of patients eligible for treatment and their subsequent CVD burden. No causal inference is sought and therefore confounding will not be addressed.
**P. Plans for addressing missing data**<br>Recording of exposures such as smoking status and BMI are unlikely to be independent of the values of variables themselves, and so does not satisfy the missing at random (MAR) requirement for multiple imputation. Data are more likely to fit the assumption required for complete case analysis (the probability of a variable record being missing is independent of the outcome, given the value of the variable and other covariates) and thus complete case analysis (with respect to BMI, calendar time, smoking status, year of birth, sex and deprivation) will be conducted.<br><br>For many variables in this study, the presence or absence of a code will be used to assign patients to one of two groups. Because the absence of a code is an outcome of interest, missing data (i.e. incorrectly absent codes) will not be identifiable, and thus cannot be addressed as part of the study. For example, some patients may have cardiovascular disease which is not coded in their record. This will result in some misclassification of patients who have cardiovascular disease as not having disease.
**Q. Patient or user group involvement (if applicable)**<br>No patient or user group involvement will occur.
**R. Plans for disseminating and communicating study results, including the presence or absence of any restrictions on the extent and timing of publication**<br>The study findings will be submitted for publication in peer-reviewed scientific journals, and will be presented at appropriate conferences and other meetings.
**S. Limitations of the study design, data sources, and analytic methods**<br>The CVD burden will be decreased among patients who take blood pressure lowering drugs. Patients who are eligible under the current NICE treatment approach are more likely to use blood pressure lowering treatment and their subsequent burden of CVD would be expected to be lower than patients who are only eligible under different treatment approaches.<br><br>One way of investigating the impact of treatment on the future burden of disease is to compare patients who are eligible for treatment, according to their use of treatment. Evidence shows that only 50% of patients eligible for treatment actually are treated.[23] This will give an indication of burden in both treated and untreated scenarios.<br><br>Statin use among the cohort will also influence burden of disease. This will have most impact when eligibility for both statins and blood pressure lowering overlap (strategies based on QRISK2 and age/sex). Therefore, in a sensitivity analysis, we will stratify by statin use.
**T. References**

Applicants must complete all sections listed below

Sections which do not apply should be completed as '*Not Applicable*'

1. Lim, S.S., et al., *A comparative risk assessment of burden of disease and injury attributable to 67 risk factors and risk factor clusters in 21 regions, 1990-2010: a systematic analysis for the Global Burden of Disease Study 2010.* Lancet, 2012. **380**(9859): p. 2224-60.
2. Law, M.R., J.K. Morris, and N.J. Wald, *Use of blood pressure lowering drugs in the prevention of cardiovascular disease: meta-analysis of 147 randomised trials in the context of expectations from prospective epidemiological studies.* Bmj, 2009. **338**: p. b1665.
3. Blood Pressure Lowering Treatment Trialists, C., et al., *Effects of different regimens to lower blood pressure on major cardiovascular events in older and younger adults: meta-analysis of randomised trials.* Bmj, 2008. **336**(7653): p. 1121-3.
4. Krause, T., et al., *Management of hypertension: summary of NICE guidance.* Bmj, 2011. **343**: p. d4891.
5. Lewington, S., et al., *Age-specific relevance of usual blood pressure to vascular mortality: a meta-analysis of individual data for one million adults in 61 prospective studies.* Lancet, 2002. **360**(9349): p. 1903-13.
6. Sundstrom, J., et al., *Effects of blood pressure reduction in mild hypertension: a systematic review and meta-analysis.* Ann Intern Med, 2015. **162**(3): p. 184-91.
7. Ettehad, D., et al., *Blood pressure lowering for prevention of cardiovascular disease and death: a systematic review and meta-analysis.* Lancet, 2015. http://dx.doi.org/10.1016/S0140-6736(15)01225-8.
8. Jackson, R., et al., *Treatment with drugs to lower blood pressure and blood cholesterol based on an individual's absolute cardiovascular risk.* Lancet, 2005. **365**(9457): p. 434-441.
9. Hippisley-Cox, J., et al., *Predicting cardiovascular risk in England and Wales: prospective derivation and validation of QRISK2.* Bmj, 2008. **336**(7659): p. 1475-82.
10. Hippisley-Cox, J., C. Coupland, and P. Brindle, *Development and validation of QRISK3 risk prediction algorithms to estimate future risk of cardiovascular disease: prospective cohort study.* Bmj, 2017. **357**: p. j2099.
11. Sundstrom, J., et al., *Blood pressure-lowering treatment based on cardiovascular risk: a meta-analysis of individual patient data.* Lancet, 2014. **384**(9943): p. 591-598.
12. Sussman, J., S. Vijan, and R. Hayward, *Using benefit-based tailored treatment to improve the use of antihypertensive medications.* Circulation, 2013. **128**(21): p. 2309-17.
13. Gaziano, T.A., et al., *Cost-effectiveness analysis of hypertension guidelines in South Africa: absolute risk versus blood pressure level.* Circulation, 2005. **112**(23): p. 3569-76.
14. Karmali, K.N., et al., *Identifying Individuals at Risk for Cardiovascular Events Across the Spectrum of Blood Pressure Levels.* J Am Heart Assoc, 2015. **4**(9): p. e002126.
15. Cobiac, L.J., et al., *Improving the cost-effectiveness of cardiovascular disease prevention in Australia: a modelling study.* BMC Public Health, 2012. **12**: p. 398.
16. Jansen, J., et al., *General practitioners' use of absolute risk versus individual risk factors in cardiovascular disease prevention: an experimental study.* Bmj Open, 2014. **4**(5).
17. Pletcher, M.J., et al., *Comparing impact and cost-effectiveness of primary prevention strategies for lipid-lowering.* Ann Intern Med, 2009. **150**(4): p. 243-54.
18. Rapsomaniki, E., et al., *Blood pressure and incidence of twelve cardiovascular diseases: lifetime risks, healthy life-years lost, and age-specific associations in 1.25 million people.* Lancet, 2014. **383**(9932): p. 1899-911.
19. Vinogradova, Y., et al., *Discontinuation and restarting in patients on statin treatment: prospective open cohort study using a primary care database.* Bmj, 2016. **353**: p. i3305.
20. Chang, K.C., et al., *Coverage of a national cardiovascular risk assessment and management programme (NHS Health Check): Retrospective database study.* Prev Med, 2015. **78**: p. 1-8.
21. Robson, J., et al., *The NHS Health Check in England: an evaluation of the first 4 years.* BMJ Open, 2016. **6**(1): p. e008840.
22. Mathur, R., et al., *Population trends in the 10-year incidence and prevalence of diabetic retinopathy in the UK: a cohort study in the Clinical Practice Research Datalink 2004-2014.* BMJ Open, 2017. **7**(2): p. e014444.
23. Joffres, M., et al., *Hypertension prevalence, awareness, treatment and control in national surveys from England, the USA and Canada, and correlation with stroke and ischaemic heart disease mortality: a cross-sectional study.* BMJ Open, 2013. **3**(8): p. e003423.

List of Appendices *(Submit all appendices as separate documents to this application)*

Appendix 1 – Medical codelist for cardiovascular disease

Appendix 2 – List of cardiovascular disease ICD10 codes and OPCS codes

Appendix 3 – Product codelist for blood pressure lowering prescriptions

Appendix 4 – Medical codelists for chronic kidney disease and target organ damage (proteinuria, hypertensive retinopathy, left ventricular hypertrophy)

Applicants must complete all sections listed below

Sections which do not apply should be completed as '*Not Applicable*'

Appendix 5 – Product codelist for statin prescriptions

Amendment 23/01/2019

Study Title

Eligibility for blood pressure lowering treatment and subsequent cardiovascular disease burden under three different treatment approaches: a cohort study

Objectives, Specific Aims and Rationale

Throughout the analysis, approach (iv) (age and sex) has been dropped.

Study Design

This is now a closed cohort. If patients do not meet inclusion criteria on 1st January 2011, then they are excluded from the study.

K. Study Population

The study population will be comprised of patients who are aged 30-79 on 1st January 2011, who do not have a diagnosis of cardiovascular disease, who are eligible for HES and IMD linkage, and with at least 12 months of follow-up in CPRD data.

- The start of follow-up will be defined as 1st January 2011.
- The end of follow-up will be the earliest of: first cardiovascular disease record, transfer out date, last collection date, or the end of the study period six months prior to the latest available data cut at time of analysis.

Exclusion criteria as described in original ISAC protocol above.

M. Exposures, Health Outcomes and Co-variates

Applicants must complete all sections listed below

Sections which do not apply should be completed as '*Not Applicable'*

Aims 1 and 2

The definition of the blood pressure only approach has been updated. This is now:

Either most recent recorded blood pressure to cohort entry ≥140mmHg systolic or ≥90mmHg diastolic,
Or using blood pressure lowering treatment at cohort entry, with either:

- two previous blood pressure measures ≥140/90 mmHg (systolic or diastolic above threshold) within a two year period,
- a hypertension diagnosis
- a flag on the general practice hypertension register

Aim 3

A new category of systolic blood pressure has been added (<110mmHg) and a new category of diastolic blood pressure has been added (<70mmHg).

Systolic and diastolic blood pressure now have the following categories:

- Systolic blood pressure; categorised at cohort entry (<110, 110-119, 120-139, 140-159, 160-179, 180+mm Hg)
- Diastolic blood pressure; categorised at cohort entry (<70, 79-79, 80-89, 90-99, 100-109, 110+mm Hg)

QRISK2 scores, systolic blood pressure and diastolic blood pressures will also be split into quintile values for this aim.

Outcomes in the main analysis will be coronary heart disease and cerebrovascular disease, measured using data from CPRD, HES and ONS. Heart failure and peripheral arterial disease will no longer be included, to align the analysis with outcomes included in QRISK2.

N. Statistical Analysis

The date at which treatment eligibility will be calculated is 1st January 2011 for all patients.

Control of blood pressure: treatment and control of blood pressure is now investigated for all strategies and categories have been updated as follows:

- Treated at cohort entry, and BP <140/90mm Hg (controlled)
- Treated at cohort entry, and BP ≥140/90mm Hg (uncontrolled)
- Untreated at cohort entry, and BP <140/90mm Hg
- Untreated at cohort entry, and BP ≥140/90mm Hg

New analyses

- The number of patients treated to avoid one event will be calculated for each strategy by dividing the number of patients eligible for treatment by the number of events avoided if all eligible patients were treated for ten years.
- In Aim 3, the rate and proportion of events will be described by quintile of QRISK2 score, systolic blood pressure and diastolic blood pressure.

Additional sensitivity analyses:

In the main analysis, patients with very high and very low blood pressures were treated in the same way as patients with normal blood pressure, which may not reflect a safe approach to treatment, as treating very low blood

**Applicants must complete all sections listed below**<br>**Sections which do not apply should be completed as '*Not Applicable*'**
pressures could result in falls, and very high blood pressures do require treatment. Therefore in this sensitivity analysis, very high and very low blood pressures will be additionally used to define eligibility under each strategy. Patients will be defined as not eligible for treatment if blood pressure was <120/70mmHg and eligible for treatment if blood pressure was >170/110mmHg.<br><br>In the main analysis, patients were included if they had a blood pressure measure in the five years before cohort entry. To account for any misclassification in blood pressures, this period will be restricted to one year in sensitivity analysis.<br><br>Patients with diabetes will be excluded in a sensitivity analysis, as their treatment guidelines are different to patients without diabetes.

RECORD statement

The RECORD statement – checklist of items, extended from the STROBE statement, that should be reported in observational studies using routinely collected health data.

	Item No.	STROBE items	Location in manuscript where items are reported	RECORD items	Location in manuscript where items are reported
**Title and abstract**					
	1	(a) Indicate the study's design with a commonly used term in the title or the abstract (b) Provide in the abstract an informative and balanced summary of what was done and what was found	Page 3	RECORD 1.1: The type of data used should be specified in the title or abstract. When possible, the name of the databases used should be included.<br><br>RECORD 1.2: If applicable, the geographic region and timeframe within which the study took place should be reported in the title or abstract.<br><br>RECORD 1.3: If linkage between databases was conducted for the study, this should be clearly stated in the title or abstract.	Page 3<br><br>Page 3<br><br>Page 3
**Introduction**					
Background rationale	2	Explain the scientific background and rationale for the investigation being reported	Page 4		
Objectives	3	State specific objectives, including any prespecified hypotheses	Page 5-6		
**Methods**					
Study Design	4	Present key elements of study design early in the paper	Page 7		
Setting	5	Describe the setting, locations, and relevant dates, including periods of recruitment, exposure, follow-up, and data collection	Page 7		
Participants	6	*(a) Cohort study* - Give the eligibility criteria, and the sources and methods of selection of participants. Describe methods of follow-up<br>*Case-control study* - Give the eligibility criteria, and the sources and methods of case ascertainment and control selection. Give the rationale for the choice of cases and controls<br>*Cross-sectional study* - Give the eligibility criteria, and the sources and methods of selection of participants<br><br>*(b) Cohort study* - For matched studies, give matching criteria and number of exposed and unexposed<br>*Case-control study* - For matched studies, give matching criteria and the number of controls per case	Page 7	RECORD 6.1: The methods of study population selection (such as codes or algorithms used to identify subjects) should be listed in detail. If this is not possible, an explanation should be provided.<br><br>RECORD 6.2: Any validation studies of the codes or algorithms used to select the population should be referenced. If validation was conducted for this study and not published elsewhere, detailed methods and results should be provided.<br><br>RECORD 6.3: If the study involved linkage of databases, consider use of a flow diagram or other graphical display to demonstrate the data linkage process, including the number of individuals with linked data at each stage.	Page 7, Appendices
Variables	7	Clearly define all outcomes, exposures, predictors, potential confounders, and effect modifiers. Give diagnostic criteria, if applicable.	Page 8	RECORD 7.1: A complete list of codes and algorithms used to classify exposures, outcomes, confounders, and effect modifiers should be provided. If these cannot be reported, an explanation should be provided.	Appendices

Data sources/ measurement	8	For each variable of interest, give sources of data and details of methods of assessment (measurement).<br>Describe comparability of assessment methods if there is more than one group	Page 8, Table 1, appendices		
Bias	9	Describe any efforts to address potential sources of bias	Page 7, inclusion and exclusion criteria, Sensitivity analyses, Page 15 Discussion		
Study size	10	Explain how the study size was arrived at	Appendix Figure 1		
Quantitative variables	11	Explain how quantitative variables were handled in the analyses. If applicable, describe which groupings were chosen, and why	Page 9		
Statistical methods	12	(a) Describe all statistical methods, including those used to control for confounding<br>(b) Describe any methods used to examine subgroups and interactions<br>(c) Explain how missing data were addressed<br>(d) *Cohort study* - If applicable, explain how loss to follow-up was addressed<br>*Case-control study* - If applicable, explain how matching of cases and controls was addressed<br>*Cross-sectional study* - If applicable, describe analytical methods taking account of sampling strategy<br>(e) Describe any sensitivity analyses	Pages 8-9		
Data access and cleaning methods		..		RECORD 12.1: Authors should describe the extent to which the investigators had access to the database population used to create the study population.<br><br>RECORD 12.2: Authors should provide information on the data cleaning methods used in the study.	Page 22
Linkage		..		RECORD 12.3: State whether the study included person-level, institutional-level, or other data linkage across two or more databases. The methods of linkage and methods of linkage quality evaluation should be provided.	n/a
**Results**					
Participants	13	(a) Report the numbers of individuals at each stage of the study (*e.g.*, numbers potentially eligible, examined for eligibility, confirmed eligible, included in the study, completing follow-up, and analysed)<br>(b) Give reasons for non-participation at each stage.<br>(c) Consider use of a flow diagram		RECORD 13.1: Describe in detail the selection of the persons included in the study (*i.e.*, study population selection) including filtering based on data quality, data availability and linkage. The selection of included persons can be described in the text and/or by means of the study flow diagram.	Appendix Figure 1
Descriptive data	14	(a) Give characteristics of study participants (*e.g.*, demographic, clinical, social) and information on exposures and potential confounders<br>(b) Indicate the number of participants with missing data for each variable of interest<br>(c) *Cohort study* - summarise follow-up time (*e.g.*, average and total amount)	Table 2 and Appendix Table 1<br><br>Table 2 and Appendix Table 1<br><br>Results, Page 10		
Outcome data	15	*Cohort study* - Report numbers of outcome events or summary measures over time	Results Page 10		

		Case-control study - Report numbers in each exposure category, or summary measures of exposure<br>*Cross-sectional study* - Report numbers of outcome events or summary measures			
Main results	16	(a) Give unadjusted estimates and, if applicable, confounder-adjusted estimates and their precision (e.g., 95% confidence interval). Make clear which confounders were adjusted for and why they were included<br>(b) Report category boundaries when continuous variables were categorized<br>(c) If relevant, consider translating estimates of relative risk into absolute risk for a meaningful time period	Pages 10-12		
Other analyses	17	Report other analyses done—e.g., analyses of subgroups and interactions, and sensitivity analyses	Pages 12-13		
**Discussion**					
Key results	18	Summarise key results with reference to study objectives	Page 13		
Limitations	19	Discuss limitations of the study, taking into account sources of potential bias or imprecision. Discuss both direction and magnitude of any potential bias	Page 15-16	RECORD 19.1: Discuss the implications of using data that were not created or collected to answer the specific research question(s). Include discussion of misclassification bias, unmeasured confounding, missing data, and changing eligibility over time, as they pertain to the study being reported.	Page 15-16
Interpretation	20	Give a cautious overall interpretation of results considering objectives, limitations, multiplicity of analyses, results from similar studies, and other relevant evidence	Pages 17		
Generalisability	21	Discuss the generalisability (external validity) of the study results	Page 15		
**Other Information**					
Funding	22	Give the source of funding and the role of the funders for the present study and, if applicable, for the original study on which the present article is based	Page 21		
Accessibility of protocol, raw data, and programming code		..		RECORD 22.1: Authors should provide information on how to access any supplemental information such as the study protocol, raw data, or programming code.	Page 22

*Reference: Benchimol EI, Smeeth L, Guttmann A, Harron K, Moher D, Petersen I, Sørensen HT, von Elm E, Langan SM, the RECORD Working Committee. The REporting of studies Conducted using Observational Routinely-collected health Data (RECORD) Statement. *PLoS Medicine* 2015; in press.

*Checklist is protected under Creative Commons Attribution (CC BY) license.
